# Ligand Electronic Properties Dictate Composition of Ni-Based Nanocrystals

**DOI:** 10.1021/jacs.5c15844

**Published:** 2025-11-14

**Authors:** Ludovic Zaza, Coline Boulanger, Krishna Kumar, Mikhail Agrachev, Aurélien Bornet, Jari Leemans, Gunnar Jeschke, Raffaella Buonsanti

**Affiliations:** † Laboratory of Nanochemistry for Energy (LNCE), Department of Chemical Sciences and Engineering, 27218 École Polytechnique Fédérale de Lausanne, CH-1950 Sion, Switzerland; ‡ Institute for Molecular Physical Science, Department of Chemistry and Applied Biosciences, 27219 ETH Zurich, Vladimir-Prelog-Weg 2, Zurich 8093, Switzerland; § Nuclear Magnetic Resonance Platform (NMRP), Department of Chemical Sciences and Engineering, Ecole Polytechnique Fédérale de Lausanne, CH-1950 Sion, Switzerland

## Abstract

Colloidal nanocrystals (NCs) composed of transition metals are appealing for several applications, particularly for those related to their catalytic and magnetic properties. Yet, the chemical principles governing their synthesis remain underexplored compared to other classes of materials. In this study, we take inspiration from molecular inorganic chemistry and implement a systematic ligand screening strategy to elucidate ligand-induced effects in the synthesis of Ni-based NCs. Specifically, we investigate the impact of organo-pnictide ligands with varying steric and electronic properties (i.e., PR_3_, AsR_3_, SbR_3_) in the synthesis of nickel, nickel phosphide, nickel arsenide and nickel antimonide NCs. Using a multimodal characterization approach, we reveal that the electronic properties of the ligands critically determine the resulting NC composition: weak σ-donor ligands promote the formation of nickel pnictides NCs via Ni-E cluster intermediates (E = P, As, Sb), while strong σ-donor ligands favor the formation of metallic Ni NCs via a Ni­(I) complex intermediate. We explain the ligand-induced reaction pathways via the reducibility of the Ni center. In addition to fundamental chemical insight, this approach enables the synthesis of a diverse library of colloidal Ni-based NCs, including multiply twinned, flower-like, and cubic Ni NCs, as well as Ni_2_P, Ni_12_P_5_, Ni_5_P_2_, Ni_5_As_2_, and NiSb NCs. Notably, we synthesize four previously unreported Ni-based NCs: multiply twinned Ni NCs, flower-like Ni NCs, Ni_5_P_2_ NCs, and Ni_5_As_2_ NCs. This systematic ligand-based approach provides a robust framework for tailoring and understanding the synthesis of transition metal NCs and beyond.

## Graphical abstract

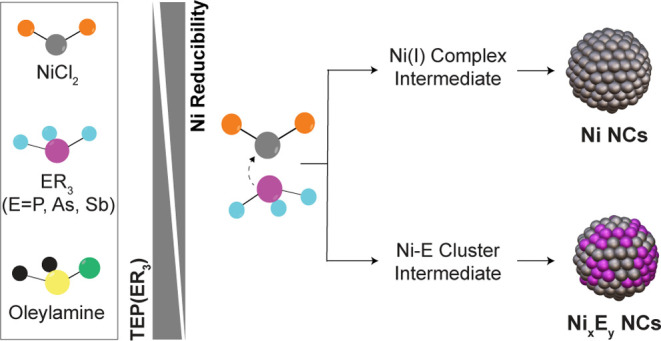

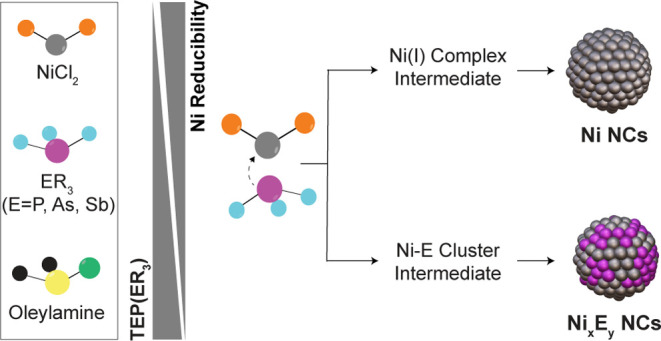

## Introduction

Synthesis design principles in materials chemistry lag behind those of molecular and organic chemistry. Colloidal chemistry for the synthesis of well-defined nanocrystals (NCs) is no exception. The progress made since the discovery of colloidal NCs has been huge and led to the 2023 Nobel Prize for quantum dots. Yet, the synthesis design of colloidal NCs with new or less explored chemical composition remains challenging and time demanding.^1^ The underlying chemistry involved in NC synthesis is complex as molecular complexes, intermediate clusters, coordination polymers, ligands, solvent and the growing NCs can all coexist and undergo chemical transformations.^2–11^ Thus, a multimodal characterization approach including different ex situ and/or in situ characterization techniques is needed.^3–12^ The only way to develop synthesis design principles to rationally tailor the NC output is to gain insights into the chemistry.^2–12^

Transition metal NCs attract interest for their composition- and facet-dependent catalytic properties.^13–30^ Metallic Ni shows facet-dependent activities in various reactions such as the hydrogenation of CO_2_ to methane, (dry) methane reforming, and ethane hydrogenolysis.^17–21^ Nickel phosphides are interesting catalysts for the CO_2_ reduction reaction, the hydrogen evolution reaction (HER) and the oxygen evolution reaction (OER).^22–28^ Nickel arsenides and antimonides are also promising OER and HER catalysts, respectively.^29,30^ Thus, understanding the chemistry involved during synthesis to tailor the composition and the shape of these NCs, which dictates the crystallographic facets that are exposed, is important.

The synthesis of colloidal transition metal-based NCs has been reported.^1,3,4,11,24,27,29–35^ However, their chemistry remains underexplored and poorly understood in comparison to noble metals.^1,36,37^ In addition, the available syntheses oftentimes involve different precursors, ligands and solvents. Considering the aforementioned chemical complexity, the key learnings are difficult to translate from one synthesis to another, and very little chemical insight is gained overall.

A common approach to gain mechanistic insight in molecular chemistry is the one factor at a time (OFAT) strategy.^38–40^ This approach relies on varying one reaction parameter such as the temperature, concentration, time or the screening of a library of one reactant (e.g., ligands with different steric and electronic properties^41,42^ ) while keeping the others constant. A few studies have demonstrated the successful application of the OFAT approach in colloidal NC synthesis.^43–47^ In one specific example, a thiourea precursor library was screened for PbS NCs synthesis.^43^ The precursors showed tunable reaction kinetics, which allowed to precisely control the NC size.^43^ In a second study, a similar approach allowed to synthesize various iron sulfide crystal phases by controlling the thiourea reactivity.^44^

Yet, these comprehensive studies remain scarce. While living in a time where data-driven chemistry and automatic experimental platforms are taking over, developing fundamental chemistry knowledge is still crucial to drive the planning of such experiments.

Inspired by the OFAT approach in molecular chemistry, we explore a library of ligands with different electronic and steric properties to determine their impact on the synthesis outcome of Ni-based NCs, including metallic nickel, nickel phosphide, nickel arsenide and nickel antimonide NCs. We use a simple system involving only three reactants: anhydrous nickel­(II) chloride salt as the metal precursor, oleylamine (OLAM) as a solvent/reducing agent and one organo-pnictide ligand (ER_3_ with E = P, As, Sb). We employ a multimodal characterization approach and determine that the Tolman electronic parameter (TEP) of the ligand dictates the composition of the final NCs. Strong σ-donor ligands lead to the formation of Ni NCs. Instead, weak σ-donor ligands react to form nickel phosphide, nickel arsenide or nickel antimonide NCs. We elucidate the reasoning behind the impact of the chemical properties of the ligands on the reaction outcome. In addition, we obtain four unreported colloidal Ni-based NCs including twinned Ni NCs, flower-like Ni NCs, Ni_5_P_2_ NCs and Ni_5_As_2_ NCs. These results offer new synthetic insights into transition metal NC formation and highlight the importance of molecular chemistry in colloidal NC synthesis.

## Results and Discussion

### The Synthetic Platform

First, we examined the very diverse synthesis routes for Ni-based NCs reported in the literature (Supplementary Note 1).^23,24,27,29,30,32,46,48–58^ Their complexity renders comparison difficult; thus, we developed a general synthesis scheme which includes only three reactants: anhydrous nickel­(II) chloride (NiCl_2_), as the nickel precursor, oleylamine (OLAM), as a solvent and reducing agent in excess amount, and the ligand of interest (Figure 1A). The NCs were collected after reactions at 250 °C for 20 min. These conditions were selected upon an initial screening. A color change of the solution from yellowish to black evidenced the formation of the NCs (Figure S1).

**Figure 1:**
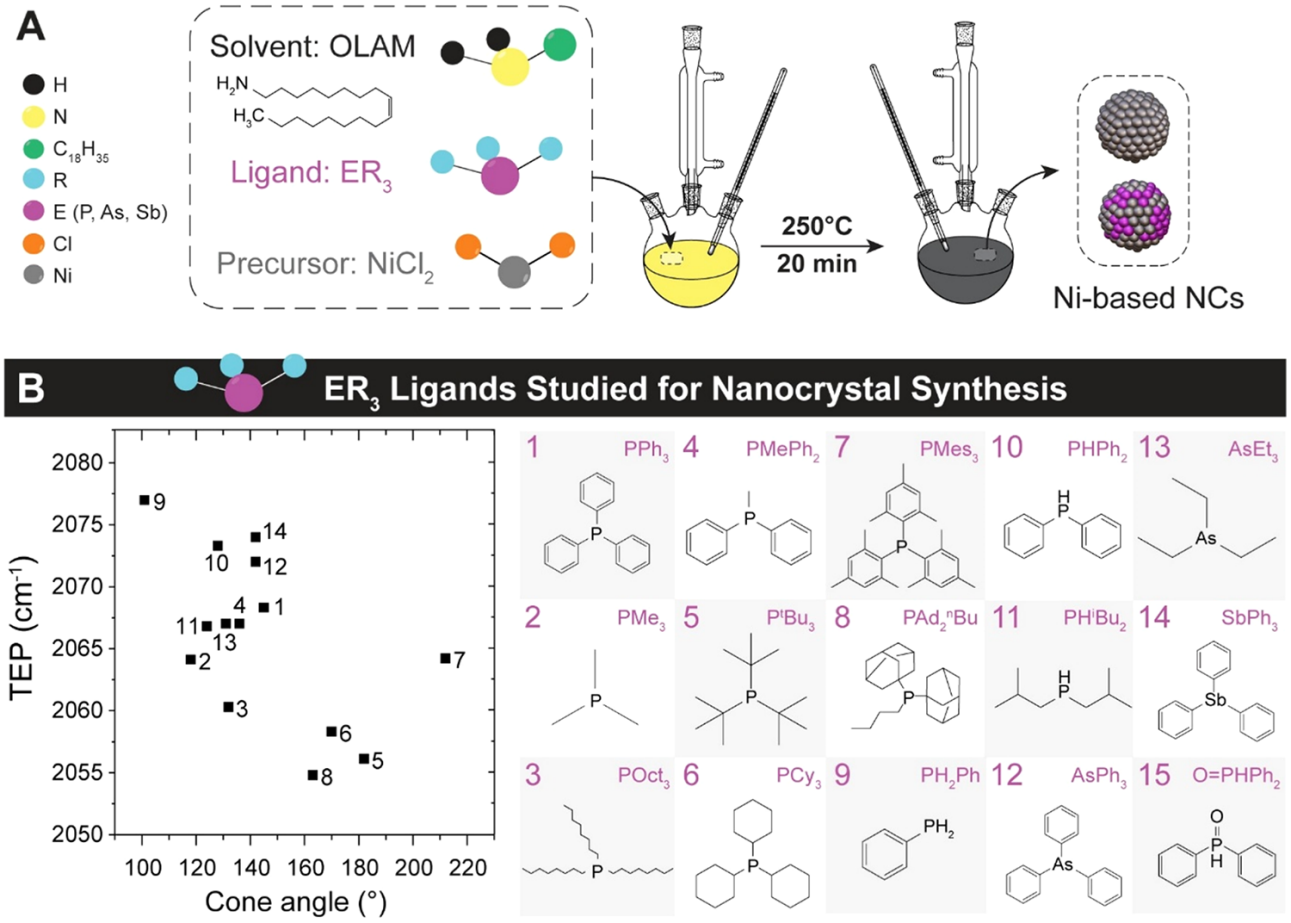
The synthetic platform for the Ni-based NCs. (A) Schematic of the reaction. (B) Ligand representation on a steric-electronic properties map given by the Tolman electronic parameter (TEP) vs the cone angle. Abbreviations: Ph = phenyl, Me = methyl, Oct = *n*-octyl, ^t^Bu = *t*-butyl, Cy = cyclohexyl, Mes = mesityl, Ad = 1-adamantyl, ^n^Bu = *n*-butyl, ^i^Bu = *i*-butyl, Et = ethyl.

We used organo-pnictide ligands (ER_3_), including phosphines (PR_3_) and their oxide (O=PR_3_), arsines (AsR_3_), and stibines (SbR_3_), in stoichiometric amounts with NiCl_2_ (2 equiv). We chose this versatile class of Lewis bases as ligands because of their ability to stabilize metals in multiple oxidation states, a property which is extensively exploited in molecular chemistry.^41,59–63^ Furthermore, their steric and electronic properties are easily tunable by systematically varying the substituents (Supplementary Note 2 and Figure S2).^41^ Finally, many of these ligands are commercially available thanks to the development in the pnictide chemistry over the past decades.^64,65^

The ligand library investigated in this study includes 15 commercially available ligands: 11 PR_3_, 1 O=PR_3_, 2 AsR_3_ and 1 SbR_3_ (Figure 1B). These ligands cover a wide range of the steric-electronic properties map given by the Tolman cone angle and the Tolman electronic parameter (TEP) (Figure 1B and Table S1).

### The library of Ni-based NCs

The NCs obtained from the synthetic screening can be divided into two main classes: Ni NCs (Figures 2 and S3–S5) and Ni_*x*_E_*y*_ NCs (E = P, As or Sb) (Figures 3 and S6–S12).

**Figure 2:**
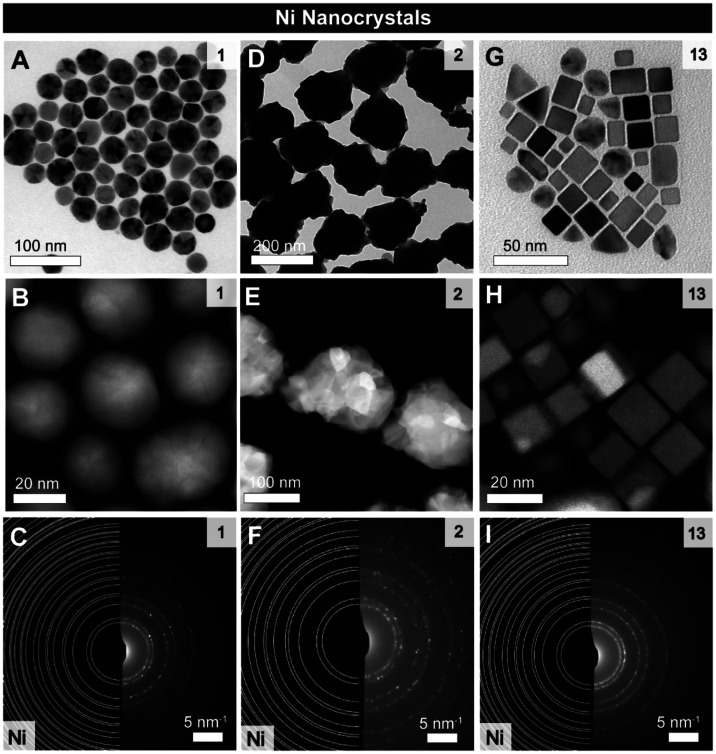
Overview of the Ni NCs obtained from the synthesis with different ligands. (A, D, G) Bright-field TEM images of the Ni NCs obtained respectively with PPh_3_ (1), PMe_3_ (2) and AsEt_3_ (13). (B, E, H) HAADF-STEM images and (C, F, I) SAED of the NCs reported in (A), (D) and (G), respectively.

**Figure 3:**
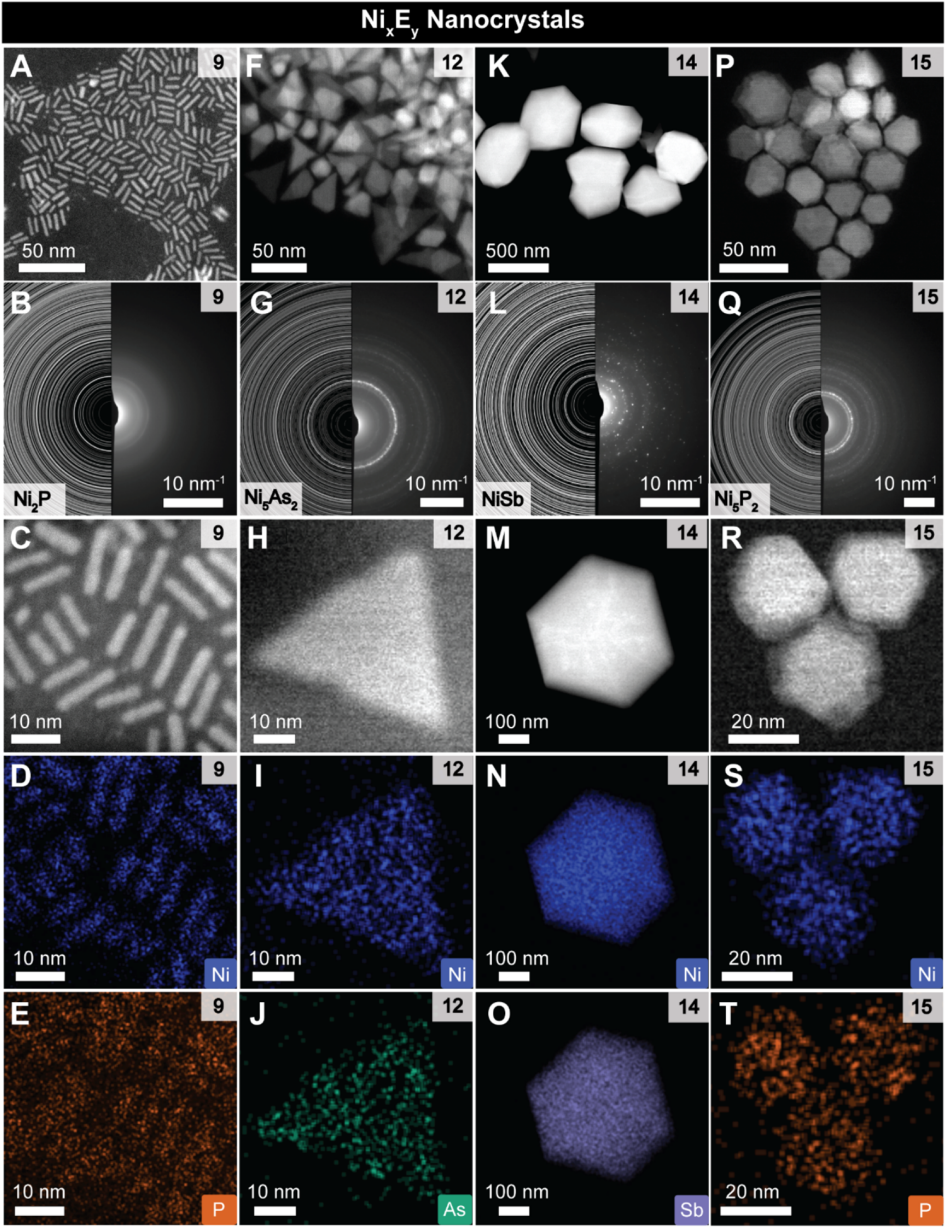
Overview of the Ni_x_E_y_ NCs obtained from the synthesis with different ligands. (A, F, K, P) Low magnification HAADF-STEM images of the Ni_*x*_E_*y*_ NCs obtained with PH_2_Ph (9), AsPh_3_ (12), SbPh_3_ (14) and O=PHPh^_2_^ (15), respectively. (B, G, L, Q) SAED of the NCs reported in (A), (F), (K) and (P) together with Ni_2_P, Ni_5_As_2_, NiSb and Ni_5_P_2_ references, respectively. (C–E), (H–J), (M–O) and (R–T) HAADF-STEM-EDX elemental mapping of the NCs reported in (A), (F), (K) and (P), respectively.

Twinned Ni NCs are the main products with PPh_3_ (**1**), POct_3_ (**3**), PMePh_2_ (**4**), P^t^Bu_3_ (**5**), PCy_3_ (**6**), PMes_3_ (**7**) and PAd_2_Bu (**8**) (Figures 2A–C and S3). Selected area electron diffraction (SAED) confirms the metallic nature of the NCs (Figure 2C), high-angle annular dark-field scanning transmission electron microscopy (HAADF-STEM) evidence twin planes (Figure 2B) and high resolution (HR)-TEM indicates the presence of both decahedral and bipyramidal NCs (Figure S4).

Ni NCs are also obtained with PMe_3_ (**2**) (Figure 2D-F). Low magnification transmission electron microscopy (TEM) shows that these NCs assemble into flower-like structures where the NCs are interconnected (Figure S5). HAADF-STEM reveals the polycrystalline nature of the NCs obtained with PMe_3_ (Figure 2E). Finally, the main product shifts to single-crystalline Ni cubes with AsEt_3_ (**13**) (Figure 2 **G-I**). This subset of ligands yielding metallic Ni NCs also show promise in directing the shape of the final product.

Among the Ni_*x*_E_*y*_ NCs, Ni_2_P NCs are obtained with PH_2_Ph (**9**) (Figures 3A–E and S6), amorphous Ni_*x*_P_*y*_ NCs with PHPh_2_ (**10**) (Figure S7), Ni_12_P_5_ NCs with PH^i^Bu_2_ (**11)** (Figure S8), Ni_5_As_2_ NCs with AsPh_3_ (**12**) (Figures 3F–J and S9), NiSb NCs with SbPh_3_ (**14**) (Figures 3K–O and S10) and Ni_5_P_2_ NCs with O=PHPh_2_ (**15**) (Figures 3P–T and S11–S12).

STEM energy-dispersive X-ray spectroscopy (STEM-EDX) quantitative elemental mapping of the Ni_2_P, Ni_12_P_5_, Ni_5_As_2_, NiSb and Ni_5_P_2_ NCs (Figures S6, S8–S11, respectively) are in good agreement with the crystalline phases assigned from SAED (Figure 3B,G,L,Q, respectively). The different compositions assigned to the NCs were verified by HR-TEM imaging (Figures S6–S8 and S12). Yet, the presence of minor impurity cannot be excluded, and phase purity is not claimed at this point due to the complexity of the Ni–P phase diagram in which many crystal phases possess very close composition (i.e., Ni_2_P, Ni_12_P_5_, Ni_5_P_2_ and Ni_3_P which are respectively composed of 33.3 at. %P, 29.4 at. %P, 28.6 at. %P and 25 at. %P).^66^

Altogether, the proposed synthetic platform showcases a great tunability of NC composition and shape under the same reaction conditions. Therefore, we proceeded to investigate the chemistry involved during the synthesis and understand the role of the ligands via a multimodal approach.

### The Electronic Properties of the Ligands Dictate the NC Composition (Ni vs Ni_*x*_E_*y*_)

The library of Ni-based NCs provides convincing evidence that the ligands play a central role in determining the final composition of the NCs.

Most of the reported syntheses of nickel phosphide NCs rely on the thermal decomposition (i.e., pyrolysis) of POct_3_ ligands above 300 °C.^24,27,53–58^ Herein, the reaction temperature is significantly lower (i.e., 250 °C) and the ligands forming nickel phosphide and arsenide NCs do not thermally decompose at this temperature. Indeed, ^31^P­{^1^H} NMR does not show any decomposition of these ligands when heated alone in OLAM without NiCl_2_ in the same reaction conditions (Figure S13). In addition, replacing NiCl_2_ with CuBr generates Cu NCs at 270 °C, which is well above our reaction temperature (Figure S14). If the utilized ligands were thermally decomposing, Cu phosphide and Cu arsenide NCs would have likely formed in these experiments instead of metallic Cu NCs. These experiments indicate that thermal decomposition of the ligands is very unlikely to be involved in the formation of the Ni_*x*_E_*y*_ NCs.

We note that a correlation exists between the electronic properties of the ER_3_ ligands and the tendency of a given ligand to form metallic Ni NCs or Ni_*x*_E_*y*_ NCs (Figure 4).

**Figure 4:**
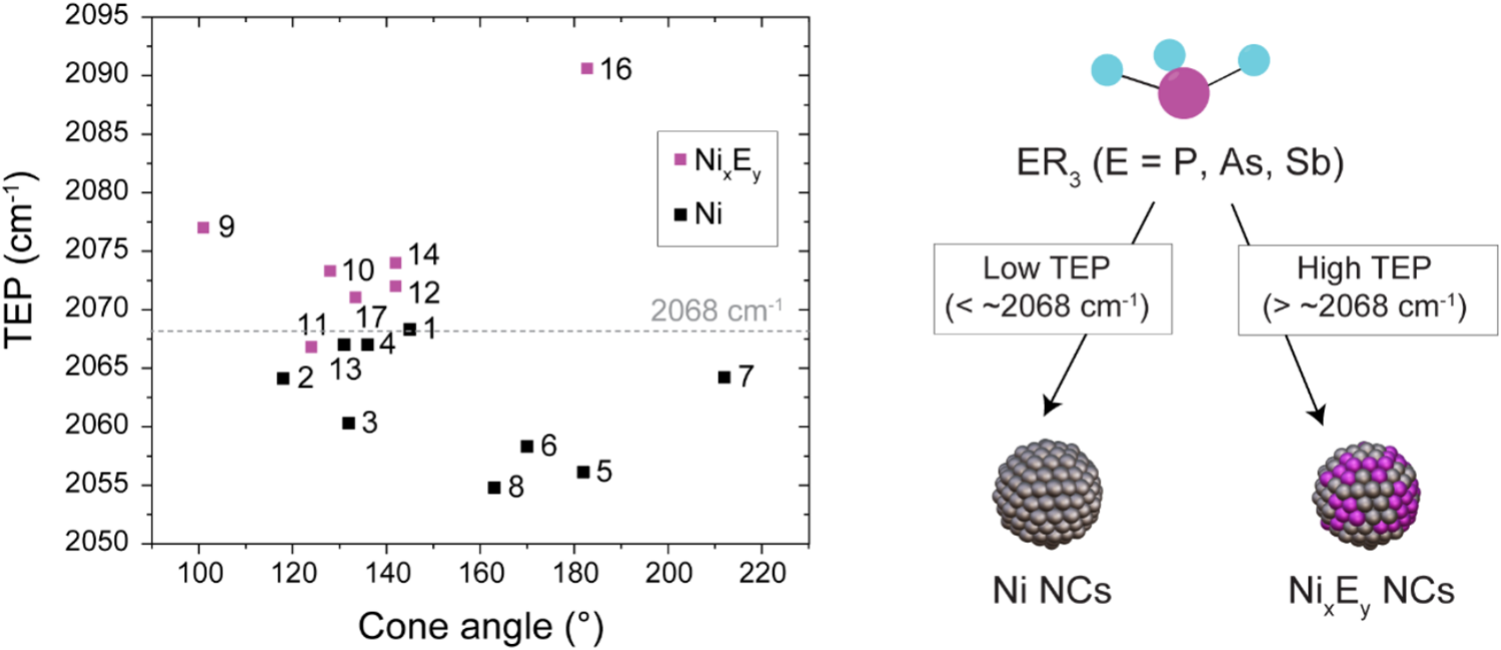
Classification of the ligands forming Ni and Ni_x_E_y_ NCs. TEP vs cone angle map of the tested ligands colored according to the NC composition outcome.

All the ligands forming metallic Ni NCs possess a TEP below 2068 cm^–1^. Instead, ligands with TEP above 2068 cm^–1^ yield phosphide, arsenide or antimonide NCs (Figure 4). This classification explains well the different results obtained with the ligands AsEt_3_ (**13**) and AsPh_3_ (**12**), which form Ni and Ni_5_As_2_ NCs (Figures 2 **G-I** and 3 **F-J**), respectively. Indeed, AsPh_3_ is a weaker σ-donor than AsEt_3_ because of the presence of alkyl substituents in the latter (TEP­(AsPh_3_) = 2072 cm^–1^ whereas TEP­(AsEt_3_) = 2067 cm^–1^). To further test this hypothesis, we performed one synthesis with P­(C_6_F_5_)_3_ (**16**) in which all of the hydrogen atoms of PPh_3_ (**1**) are replaced by electronegative fluorine atoms, rendering the P­(C_6_F_5_)_3_ a much weaker σ-donor than PPh_3_ (TEP­(PPh_3_) = 2068.9 cm^–1^, TEP­(P­(C_6_F_5_)_3_) = 2090.9 cm^–1^).^41^ In agreement with the hypothesis made, NCs including both nickel and phosphorus are obtained with P­(C_6_F_5_)_3_ (Figure S15). Finally, we also tested P­(OEt)­Ph_2_ (**17**) for which the substitution of one phenyl group of PPh_3_ by an ethoxy group leads to a significant increase of the TEP to 2071.6 cm^–1^.^41^ Experimental results confirm our expectations and indicate the formation of Ni_5_P_2_ NCs (Figure S16).

Steric properties do not seem to play a significant role in the final composition of the NCs (Figure 4). For example, Ni NCs are obtained with both PMe_3_ (**2**) (Figure 2D-F) and PMes_3_ (**7**) (Figure S3E), which are representative examples of a sterically unhindered and hindered phosphines with their respective cone angle of 118° and 212°.^41^ Similarly, ligands with the same cone angle lead to completely different products. For example, AsEt_3_ (**13**) with a cone angle of 131° forms Ni NCs (Figure 2G-I) but PHPh_2_ (**10**) with a cone angle of 128° forms nickel phosphide NCs (Figure S7). Overall, these observations suggest that the electronic properties of the ligands are important to control the NC composition.

### The Chemical Reactions Involved in the Synthesis of Ni NCs

Having excluded thermal decomposition and identified the importance of the ligand electronic properties, we proceeded to assess the chemical reactions involved in the synthesis of Ni NCs.

We collected aliquots at different temperatures during the heating ramp for the synthesis with PPh_3_ (**1**) and POct_3_ (**3**), as representative cases, and we investigated these aliquots by ^31^P­{^1^H} nuclear magnetic resonance (NMR), ^1^H NMR, electron paramagnetic resonance spectroscopy (EPR) and ultraviolet–visible spectroscopy (UV–vis) (Figures 5 and S17–S24).

**Figure 5:**
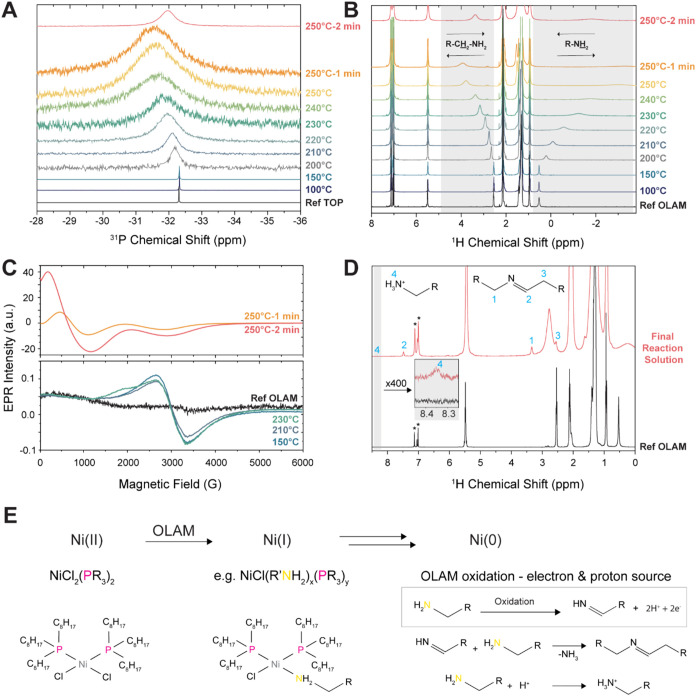
Chemical insights into the synthesis of Ni NCs. (A) ^31^P­{^1^H} NMR spectra of aliquots taken at different temperatures during the Ni NC synthesis with POct_3_ (3). (B) ^1^H NMR spectra of the aliquots. The aliquots were extracted at the indicated temperature and measured at room temperature with NMR. The proton resonances highlighted in gray are those shifting during the Ni NC synthesis. (C) EPR spectroscopy of the aliquots measured at 298 K. (D) ^1^H NMR spectra of the final reaction solution. New resonances which can be attributed to aldimine (1–3) and to ammonium (4) species appear. The resonances indicated by (*) originate from toluene. (E) Proposed chemical reactions and possible species present in solution during the synthesis of the Ni NCs. The information collected herein for POct_3_ (3) suggests that formation of Ni NCs proceeds via the formation of Ni­(I) complex intermediates via the reduction by OLAM.

^31^P­{^1^H} NMR shows a transient and gradual downfield shift and broadening of the phosphorus signal, which reaches a maximum after 1 min at 250 °C before shifting back upfield at a later stage (Figures 5A and S18). Similarly, ^1^H NMR shows broadening and downfield shifts for the OLAM R-CH_2_-NH_2_ and upfield shifts for the R-NH_2_ resonance (Figure 5B). These observations suggest the presence of paramagnetic Ni­(I) centers in the solution whose unpaired electron shifts the NMR resonances because of spin-electron interactions and broadens the signal by accelerating the relaxation of the nuclei.^67–69^ The shifts and broadenings of the ^31^P and ^1^H resonances are synchronized. These changes affect both POct_3_ and OLAM. The effects are limited to the vicinity of the pnictogen atoms, suggesting that OLAM and POct_3_ interact with the Ni­(I) centers through their respective N and P atoms.

To identify the presence of Ni­(I) species, we conducted EPR spectroscopy on the collected aliquots at 298 K (Figure 5C). While no signal is observed in the OLAM reference, an isotropic peak around g = 2.19 (B≈3060 G) with peak-to peak line width ≈1000 G appears in the 150 and 210 °C aliquots which can be attributed to a dissolved Ni­(I) complex.^70^ The same signal is observed in the 230 °C aliquot, with an additional shoulder at lower fields (*B* ≈ 2000 G) which likely originates from a different Ni­(I) species, which only forms at 230 °C. The aliquot collected after 1 min at 250 °C shows a very broad, strong and anisotropic signal, which is attributed to relatively large (ferro)­magnetic Ni clusters.^71–75^ A similar signal, with higher intensity and slightly increased line width, is observed for the 250 °C aliquot after 2 min, which indicates Ni NC growth. We note that the Ni­(I) complexes are stable in solution and could be stored for more than 3 weeks under a nitrogen atmosphere.

These Ni­(I) complex intermediates must form by reduction of Ni­(II). OLAM is a well-known reducing agent in NC synthesis.^76^ Thus, the most likely assumption is that the intermediates and the subsequent Ni NCs are formed by the direct reduction of Ni­(II) by OLAM. Several control experiments confirmed this assumption (Figures 5D and S19–S24). In particular, the NCs did not form from the reaction of the NiCl_2_ and the ligands in a nonreductive solvent (Figures S19–S22). Metallic Ni aggregates formed from the reaction between NiCl_2_ and OLAM in the absence of ER_3_ ligands (Figure S23). Importantly, new resonances consistent with the oxidation product of OLAM appear in the ^1^H NMR spectrum of the final reaction solution, which confirm the role of OLAM as a reducing agent in the reaction (Figures 5D and S24).^2,77,78^

In addition, the formation of NiCl_2_L_2_ complexes is plausible due to the use of two equivalents of the ligands (L) with respect to NiCl_2_,^79–83^ and the ease with which they form in an organic solvent.^84–90^ Indeed, UV–vis did evidence the formation of NiCl_2_(POct_3_)_2_ complexes at temperature ≥ 220 °C (Figure S17). Complementary experiments were not conclusive regarding the presence of these complexes in the initial stages of the synthesis when mixing NiCl_2_ and the ligands (Supplementary Note 3 and Figures S25–S29). However, presynthesized NiCl_2_(PPh_3_)_2_ and NiCl_2_(PMe_3_)_2_ complexes did lead to the formation of similar NCs which indicate their likely involvement in the reaction (Figure S30).

Based on all the above, a mechanistic picture shapes up for the formation of the Ni NCs (Figure 5E). We propose that NiCl_2_L_2_ complexes are reduced by OLAM into Ni­(I) intermediates. These Ni­(I) intermediates might contain both OLAM and the phosphine ligands, however their chemical nature is currently hypothetical. These Ni­(I) intermediates accumulate in solution and are then further reduced to Ni(0) via OLAM oxidation. The formation of the Ni NCs can occur either through reduction followed by bonding of Ni(0) atoms or formation of clusters containing Ni­(I) species followed by reduction with OLAM.^91^ Future studies combining in situ X-ray absorption spectroscopy (XAS) and small-angle X-ray scattering (SAXS) might elucidate this aspect.

### The Chemical Reactions Involved in the Synthesis of Ni_*x*_E_*y*_ NCs

Ligands PH_2_Ph (**9**), PHPh_2_ (**10**), PH^i^Bu_2_ (**11)**, AsPh_3_ (**12**), SbPh_3_ (**14**) and O=PHPh_2_ (**15**) yield to Ni_*x*_E_*y*_ NCs wherein a chemical bond forms between Ni and P, As or Sb.

To gain further insight into the reaction mechanism, we monitored the synthesis of Ni_*x*_E_*y*_ NCs with PHPh_2_ (**10**), as a representative example, by collecting aliquots at different temperatures during the heating ramp (Figure 6). First, we analyzed these aliquots by ^31^P­{^1^H} NMR (Figure 6A). In strong contrast to the ligands forming Ni NCs (Figures 5A and S18), ^31^P­{^1^H} NMR reveals resonances associated with the formation of new phosphorus-containing species. The multiplicity in the resonances at 7.5 ppm (doublet), 7.8 ppm (triplet), 67.5 ppm (doublet) and 69 ppm (triplet) indicates the formation of molecules in which multiple P nuclei are coupled together (Figure 6A, panels 1 and 4). The coupling constants between the different phosphorus atoms are in the range of |*J*_PP_| ≈ 20–25 Hz, which is expected for ^2^ *J*_PP_ rather than for ^1^ *J*_PP_ which are typically ≥100 Hz.^92–95^ The coupling constants indicate that the phosphorus atoms are separated by at least one nucleus in the molecular backbone, which is consistent with the formation of Ni_*x*_P_*y*_ species.^92–95^ ^1^H–^31^P heteronuclear multiple bond correlation (HMBC) evidences that the phosphorus nuclei in these molecules are coupled to aromatic protons in the 7–7.5 ppm region and to two proton signals in the 6.8–5.8 ppm region with a 1:1 integration ratio, which are expected for P–H coupling (Figure 6B). Compared to the PHPh_2_ reference (i.e., the phosphorus resonance at −40.8 ppm), the P–H protons are significantly downfield and the coupling constant increases from 213 to 310–330 Hz, which indicates a decrease of the electron density on the phosphorus and is consistent with the formation of a Ni–P bond.^92^ The large new resonance at −5 ppm is attributed to triphenylphosphine (PPh_3_) by comparison with PPh_3_ reference and by ^1^H–^31^P HMBC which reveals that only aromatic protons are coupled to the −5 ppm phosphorus signal (Figure 6A, panel 5 and Figure 6B). The phosphorus signal at −15 ppm is consistent with the expected resonance of tetraphenyldiphosphine (Ph_2_P-PPh_2_),^92,96^ which can be formed by the oxidative coupling of PHPh_2_ with Ni­(II) salts (Figure S31).^97^ We could not formally identify the three remaining phosphorus resonances at 6.8, 26.8, and 41.3 ppm. However, we discuss possible candidates in Supplementary Note 4.

**Figure 6:**
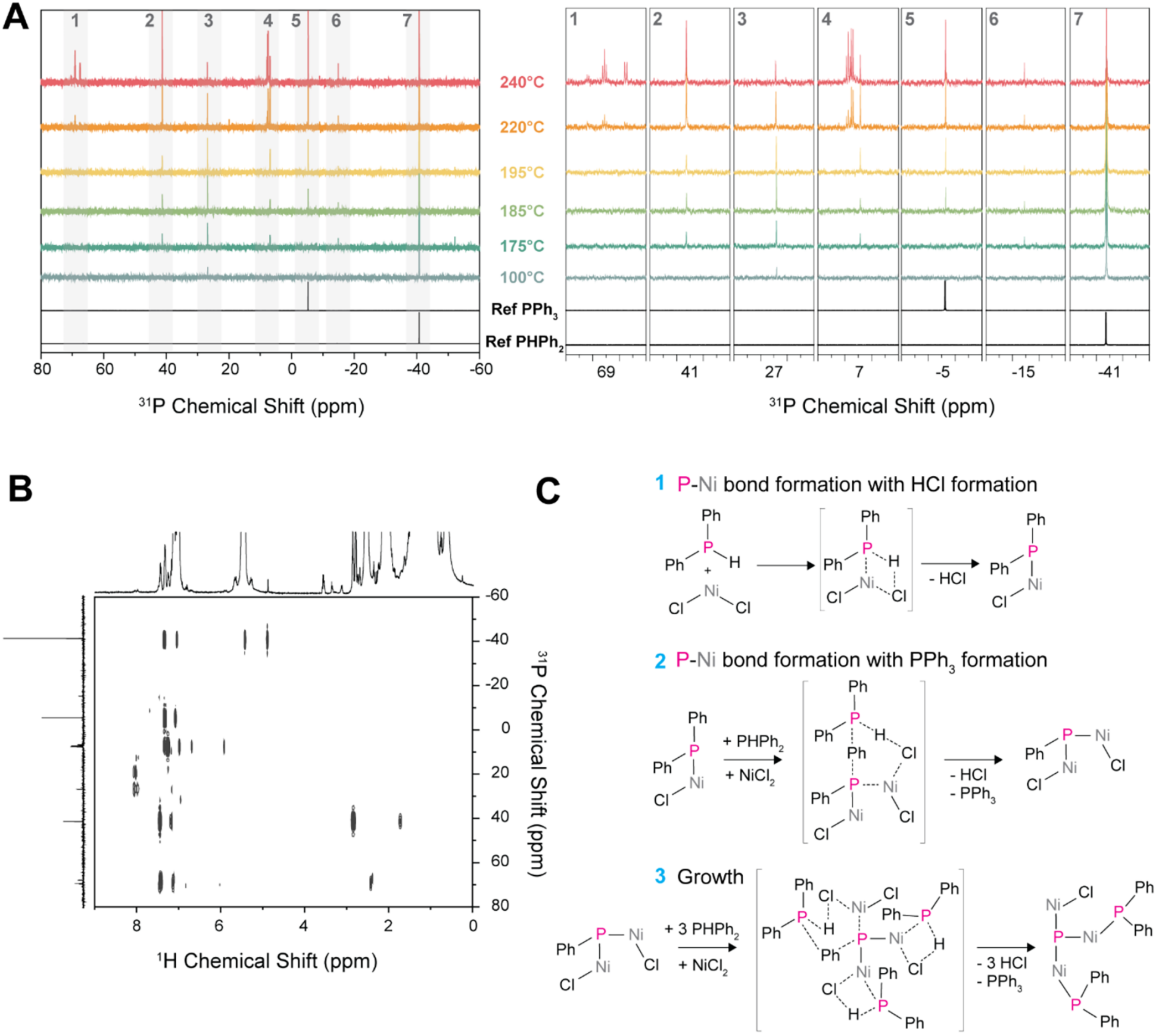
Chemical insights into the synthesis of Ni_x_E_y_ NCs. (A) ^31^P­{^1^H} NMR spectra of aliquots taken at different temperature and times during the Ni_*x*_E_*y*_ NC synthesis with PHPh_2_ (10). (B) ^1^H–^31^P HMBC of the aliquot taken at 240 °C from the same synthesis in (A). (C) Proposed chemical reactions leading to the formation of Ni–P bonds, HCl, PPh_3_ and NC growth. The aliquots were extracted at the indicated temperature and measured at room temperature with NMR. The information collected herein for PHPh_2_ (10) suggests that formation of Ni_*x*_E_*y*_ NCs proceeds via the formation of Ni-E cluster intermediates followed by a condensation reaction.

Based on these results, a mechanistic picture shapes up for the formation of the Ni_*x*_E_*y*_ NCs (Figure 6C). The formation of the Ni–P occurs through two different mechanisms. First, NiCl_2_ reacts with PHPh_2_ forming NiCl­(PPh_2_) with a covalent Ni–P bond with the release of HCl (Figure 6C, step 1). The formation of HCl is consistent with studies in which hydrogen halides form during the synthesis of phosphido complexes of group 10 metals (i.e., Ni, Pd and Pt) when MX_2_ salts react with secondary phosphines in the presence of a base.^98–102^ Here, OLAM can act as a base due to the presence of terminal -NH_2_ amino groups. In a second step, a NiCl_2_ molecule reacts with the NiCl­(PPh_2_) phosphido group assisted with another PHPh_2_ molecule to form HCl and PPh_3_ (Figure 6C, step 2). Through this process, the ligands (i.e., PHPh_2_) play a double role during the nucleation stage. They act as the phosphorus source and as the phenyl acceptor of other reacted ligands. This double role might explain the stoichiometry of the final NCs which are enriched in Ni (50–72 at% Ni and 50–28 at% P/As/Sb) compared to the initial reaction stichometry (33 at% Ni and 67 at% P/As/Sb). It is also interesting to note that only phenyl rings are accepted by other PHPh_2_ ligand. There is no evidence of the acceptance of Cl or H atoms which would lead to the formation of PClPh_2_ or PH_2_Ph respectively (Figure S32). Finally, as no pyrolysis of the ligands occurs at the temperature of the proposed synthetic scheme, we postulate that molecules containing multiple Ni and P atoms evolve into the final Ni_*x*_E_*y*_ NCs via condensation reactions with the loss of HCl and PPh_3_ (Figure 6C, step 3).^5,103,104^

We note that the detailed mechanism leading to the formation of the Ni–P cluster intermediates might change among the screened phosphines. The reaction with PHPh_2_ yields HCl and PPh_3_ (Figure 6), suggesting a general pathway for P–H containing phosphines. In contrast, (OR)-substituted ligands like P­(OEt)­Ph_2_ (**17**) might produce organic hypochlorites as suggested in the case of P­(OR)_3_ ligands.^46^ Finally, E-C bond cleavage with the generation of biaryl and chloroaryl product might even occur in the absence of E-H or E-O bonds for AsPh_3_ (**12**) or P­(C_6_F_5_)_3_ (**16**).^105^

Here, OLAM does not play a crucial role. Indeed, the reaction of NiCl_2_ with the ligands in ODE without OLAM yields to Ni_*x*_E_*y*_ NCs (Figures S33–S36). Furthermore, the capacity of the ligands to form a stable Ni-E bond also emerges from experiments where presynthesized Ni NCs reacted with the ligands under NC synthesis conditions (Figures S37–S43).

### Chemical Lesson Learned and Outlook

Figure 7 summarizes the overall insight into the Ni-based NC synthesis using a library of organo-pnictide ligands.

**Figure 7:**
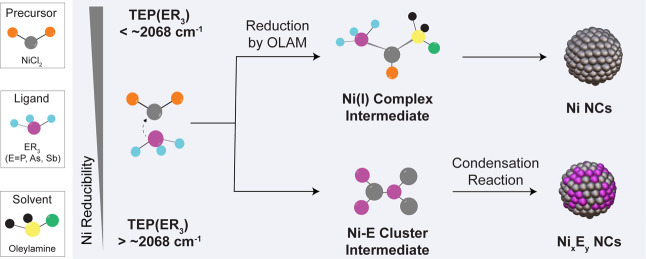
Schematic representation of the ligand chemistry behind the formation of Ni and Ni_x_E_y_ NCs. Ni NCs form via a reductive pathway of Ni­(I) complex intermediates from ligands with lower TEP. Ni_*x*_E_*y*_ NCs form via a condensation reaction involving Ni-E cluster intermediates from ligands with higher TEP. Key in determining the reaction mechanism is the ligand-dependent reducibility of the Ni center.

The formation of purely metallic Ni NCs relies on the reduction of Ni­(II) in the form of NiCl_2_L_2_ complexes to Ni­(I) intermediates and, eventually, to Ni(0) driven by OLAM oxidation. This mechanism takes place in the presence of ligands with a TEP smaller than ∼2068 cm^–1^.

The formation of Ni phosphide, arsenide or antimonide NCs relies on a condensation reaction of some sort of Ni-E cluster intermediates, similarly to what reported for the synthesis of some binary and doped NCs from single source precursors.^5,103,104^ This mechanism takes place in the presence of ligands with a TEP larger than ∼2068 cm^–1^.

We rationalize the two different reaction pathways based on ligand effect on the Ni reducibility by OLAM. Ligands with TEP values below ∼2068 cm^–1^ are stronger σ-donors and Lewis bases which will favor the formation of NiCl_2_L_2_ and will increase the reducibility of the Ni center by OLAM, thus favoring the reduction pathway toward the Ni­(I) intermediate. As the TEP increases above ∼2068 cm^–1^, the formation of NiCl_2_L_2_ becomes less favorable and the OLAM most likely is not a sufficiently strong reducing agent for the Ni­(II) at the start of the reaction, which eventually results in a different reaction pathway.

A series of six different organophosphite ligands including P­(OMe)_3_, P­(OEt)_3_, P­(O^n^Bu)_3_, P­(O–CH_2_–^t^Bu)_3_, P­(O^i^Pr)_3_ and P­(OPh)_3_ with TEP > 2075.9 cm^–1^ were previously reported to form Ni_*x*_P_*y*_ NCs, which is in line with our proposed criterion.^46^ However, a precedent exists for making NiP_2_ NCs with P­(NEt_2_)_3_ which has a TEP of 2061.9 cm^–1^.^200,106^ This deviation might result from a different mechanism taking place under the reaction conditions used by the authors. Alternatively, the different behaviour might suggest that additional ligand properties should be considered to account for a balance between the Ni-E and the E-N bond strength. We also note that higher reaction temperatures, when ligand pyrolysis occurs, will result in a different outcome. For example, the products shift from Ni to Ni_*x*_P_*y*_ NCs with PPh_3_ (**1**) and POct_3_ (**3**) at 300 °C.^24,27,53–58^

Interestingly, we obtain different nickel phosphide crystal phases such as Ni_2_P and Ni_12_P_5_ at lower temperatures compared to existing syntheses, which rely on ligand pyrolysis.^24,27,53–58^ In addition, this synthetic scheme allows for the synthesis of unreported Ni_5_P_2_ and Ni_5_As_2_ NCs. The different crystal phases obtained might be linked to different reaction kinetics during the NiCl_2_-ligand chemical reactions and during possible Ni(0)-ligand chemical reactions.

Ligands must play a role also in the different NC morphologies obtained in the synthesis, although this role remains speculative at present. Surface passivating effects and different reduction kinetics of the intermediate species with Ni­(I) or Ni(0) centers can both play a role in the different shapes of Ni NCs.^106–110^ In particular, steric and electronic effects of the utilized ligands might be important. For example, PMe_3_ (**2**) has the smallest cone angle among all the ligands leading to Ni NCs, which could account for the flower-like shape (Figure 2D–F). AsEt_3_ (**13**), which leads to the formation of cubical Ni NCs (Figure 2G–I), contains an As central atom instead of P.

Follow-up in situ measurements would be beneficial to study Ni speciation and to confirm the reactions pathway and kinetics for the different NC syntheses as well as the ligand role in shape control. This work provides motivation toward future studies by highlighting the significant chemical implications resulting from the use of different ligands in similar reaction conditions upon screening an unprecedented ligand library.

## Conclusion

In conclusion, we proposed a synthetic framework to obtain colloidal Ni-based NCs and gain fundamental chemistry insights by exploring a diverse ligand library which included phosphines, arsines and stibines. We established the crucial role of the ligand electronic properties in determining the synthesis pathway and outcome. In addition to this new chemical knowledge illustrating design rules for Ni-based NCs, we obtained unreported NCs, such as twinned Ni NCs, flower-like Ni NCs, Ni_5_P_2_ NCs and Ni_5_As_2_ NCs.

We believe that the implications of these findings go beyond Ni because of the high affinity of phosphine ligands toward many different metals in various oxidation states and the ease with which these ligands can be tuned. Even further, this work showcases how a molecular approach to NC synthesis helps to develop design rules for their synthesis beyond the current state of the art encouraging other researchers to pursue a similar approach.

## Experimental Section

### Chemicals

All chemicals were used as received, with no further purification. Oleylamine (OLAM, technical grade, 70%), 1-octadecene (ODE, technical grade, 90%), nitric acid (70%), toluene (anhydrous, 99.8%), toluene-d8 (99% atom D), diphenylphospine (PHPh_2_, 98%), triphenylphosphine (PPh_3_, ReagentPlus 99%), triphenylarsine (AsPh_3_, 97%), triphenylstibine (SbPh_3_, 99%), tri*tert*-butylphosphine (P^t^Bu_3_, 98%) and di­(1-adamantyl)-*n*-butylphosphine (cataCXium A, 95%) were purchased from Sigma-Aldrich. Hexane (anhydrous, 96%), methyldiphenylphosphine (PPh_2_Me, 97%) and diphenylphosphine oxide (O=PHPh_2_, 98%) were purchased from TCI. Triethylarsine (AsEt_3_, 99%) was purchased from Strem Chemicals. Tris­(pentafluorophenyl)­phosphine (P­(C_6_F_5_)_3_, 99%) was purchased from Apollo Scientific. Ethyl diphenylphosphinite (P­(OEt)­Ph_2_, 95%), and bis­(triphenylphosphine)­nickel­(II) dichloride (NiCl_2_(PPh_3_)_2_, 98%) were purchased from Fluorochem. Tri-n-octylphosphine (POct_3_, TOP, technical grade 90%), tris­(2,4,6-trimethylphenyl)­phosphine (P­(mesityl)_3_, 98%) and tricyclohexylphosphine (PCy_3_, 98%) were purchased from Thermo Scientific Chemicals Alfa Aesar. Chlorodiphenylphosphine (PClPh_2_, 98%) was purchased from Thermo Scientific Chemicals Acros. Nickel­(II) chloride (NiCl_2_, anhydrous 98%), phenylphosphine (PH_2_Ph, 99%), trimethylphosphine (PMe_3_, 99%) and di-iso-butylphosphine (PH^i^Bu_2_, 97%) were purchased from ABCR.

*Caution: Trimethylphosphine (2), tritert-butylphosphine (5), phenylphosphine (9), diphenylphosphine (10) and di-iso-butylphosphine (11) are pyrophoric materials and should only be used under N*_2_ *protective environment with proper personal protective equipment and training. Triphenylarsine (12), triethylarsine (13) and triphenylstibine (14) are toxic materials and should only be used with proper personal protective equipment and training.*

### Characterization

#### Transmission Electron Microscopy (TEM) and Selected Area Electron Diffraction (SAED)

Bright-field TEM and SAED images were acquired on a FEI Tecnai-Spirit at 120 kV, equipped with a Gatan Orius SC200D camera. As-synthesized NCs were drop-casted on a Cu TEM grid (Ted Pella, Inc., 01813-F) before imaging. The SAED patterns were analyzed with the software CrysTBox.^111^ The CIF files used for analysis were downloaded from the Materials Project.^112^

#### High-Resolution TEM (HR-TEM)

*HR-*TEM images were acquired on a probe-corrected Thermo Fisher Scientific Spectra 200 (S)­TEM operated at 200 kV. This microscope is equipped with a high brightness X-CFEG, Super-X EDX acquisition system and Velox acquisition software. The diffraction patterns of single NCs were analyzed with the software CrysTBox.^111^ The CIF files used for analysis were downloaded from the Materials Project.^112^

#### High-Angle Annular Dark Field Scanning Transmission Electron Microscopy (HAADF-STEM) and HAADF-STEM-Energy Dispersive X-ray Spectroscopy (EDX)

Most of the HAADF-STEM­(-EDX) images were acquired on a probe-corrected Thermo Fisher Scientific Spectra 200 (S)­TEM operated at 200 kV. This microscope is equipped with a high brightness X-CFEG, Super-X EDX acquisition system and Velox acquisition software. Few images were also acquired on a FEI Tecnai Osiris at an accelerating voltage of 200 kV. This microscope was equipped with a high brightness X-FEG (Field Emission Gun), silicon drift Super-X EDX detectors and Bruker Esprit acquisition software. As-synthesized NCs were drop-casted on an Au TEM grid (Ted Pella, Inc. 01814G-F) before imaging.

#### Inductively Coupled Plasma Optical Emission Spectroscopy (ICP-OES)

ICP-OES measurements were carried out using an Agilent 5110 inductively coupled plasma optical emission spectrometry system with a VistaChip II CCD detector to determine the concentration of Ni in stock solutions. Five standard solutions (0.1, 0.5, 1, 5, and 10 ppm) were prepared in 2% HNO_3_ to obtain calibration curves for Ni to determine the concentrations. The sample solution was prepared by digesting the nanocrystals in 70% high-purity HNO_3_ (Sigma-Aldrich, purified by redistillation, > 99.999% trace metals basis) and leaving it overnight to provide full material dissolution. Then, ultrapure water was added to dilute the acid concentration to 2% for analysis.

#### Nuclear Magnetic Resonance Spectroscopy (NMR)

Solution NMR measurements were recorded on a Bruker Avance III HD 400 MHz 9.4 T spectrometer equipped with a BBFO liquid probe. One-dimensional ^31^P spectra were acquired using a standard pulse sequence from the Bruker library. The deuterated solvent used for all NMR experiments is toluene-d8. All measurements were performed at room temperature (298 K).

#### Electron Paramagnetic Resonance Spectroscopy (EPR)

EPR data were collected by continuous wave electron paramagnetic resonance (CW-EPR) spectroscopy experiments at room temperature (298 K) on a Bruker Elexsys E500 spectrometer operating at X-band frequencies, using an ER4102ST microwave resonator. All CW-EPR spectra were acquired with the following spectrometer parameters: microwave frequency = 9.4 GHz, sweep width = 595 mT, center field = 300 mT, modulation frequency = 100 kHz, modulation amplitude = 3 G, microwave power = 1.993 mW, power attenuation = 20 dB, conversion time = 164 ms. All measured g-factors were offset-corrected against a known standard (i.e., free radical 1,1-diphenyl-2-picrylhydrazyl).

#### Ultraviolet–Visible Spectroscopy (UV–vis)

Absorbance spectra were collected on a PerkinElmer Lambda 950 spectrophotometer equipped with deuterium and tungsten halide light sources and a photomultiplier tube with Peltier-controlled PbS detector. The absorption of reaction solution aliquots was measured between 250 and 700 nm in a 0.5-mL quartz cuvette.

### Synthesis

#### General Considerations

All syntheses and manipulations of Ni-based NCs were done under a dry N_2_ atmosphere, using Schlenk-line techniques or a glovebox. Anhydrous organic solvents were used for the manipulation, analysis and storage of Ni-based NCs. All glassware was oven-dried prior to use. Concentrated nitric acid was used to remove any metallic residues from the reaction flask after each reaction and the flask was then washed thoroughly with ultrapure water prior to oven drying. A J-KEM Scientific Model 310 temperature controller was used with a heating mantle for reaction temperature control.

#### Synthesis of Ni-Based NCs

In a glovebox, nickel­(II) chloride powder (58 mg, 450 μmol) and the ligand (900 μmol) were added to a 3-neck flask. Afterward, a stirring magnet and 14 mL of predegassed OLAM were added. The flask was sealed and quickly connected to a Schlenk line under N_2_. The reaction solution was then degassed 5 min at 60 °C before being heated 20 min under N_2_ at 250 °C with a heating ramp of ∼25 °C/min. The solution, originally yellow, turned black at around 200 °C (in the range of 160–240 °C). The 20 min reaction was started once the temperature reached 250 °C. At the end of the reaction, the reaction mixture was cooled down to approximately 80 °C before being transferred to the glovebox. The reaction solution was transferred to one 50 mL centrifuge tube to which 15 mL of hexane were added. The tube was sonicated and centrifuged at 13,000 rpm for 10 min. After centrifugation, the supernatant was discarded. The solid precipitate was redispersed in 5 mL of hexane and 5 mL of ethanol. A second washing was performed following the same procedure. Finally, the final solid product was collected and dissolved in 2 mL toluene for storage and further analysis.

900 μmol of ligands corresponds to respectively 236 mg of PPh_3_ (**1**), 91 μL of PMe_3_ (**2**), 401 μL of POct_3_ (**3**), 167 μL of PPh_2_Me (**4**), 222 μL of P^t^Bu_3_ (**5**), 252 mg of PCy_3_ (**6**), 350 mg of P­(mesityl)_3_ (**7**), 323 mg of cataCXium A (**8**), 99 μL of PH_2_Ph (**9**), 157 μL of PHPh_2_ (**10**), 170 μL of PH^i^Bu_2_ (**11**), 276 mg of AsPh_3_ (**12**), 127 μL of AsEt_3_ (**13**), 317 mg of SbPh_3_ (**14**), 182 mg of O=PHPh_2_ (**15**), 479 mg of P­(C_6_F_5_)_3_ (**16**) and 195 μL of P­(OEt)­Ph_2_ (**17**).

#### Aliquot Collection

The aliquots for analysis were directly extracted from the reaction solution with a syringe at the designated temperature. The aliquots were instantaneously quenched in N_2_-inerted vials containing 400 μL of anhydrous toluene. The vials were then transferred to a glovebox for storage and analysis.

## Supplementary Material



## Data Availability

All data are available in the main text or the Supporting Information. Experimental data are openly available in Zenodo at: 10.5281/zenodo.15228190

## Notes

Cone angle/TEP table of the ligands used, equations, supplementary notes, photographs of the reaction solution colors, additional TEM images, SAED and HAADF-STEM-EDX images of NCs, HRTEM image of twinned Ni NCs, HAADF-STEM-EDX spectra with quantitative at% of NC composition, HRTEM and FFT analysis of the NCs, additional ^1^H and ^31^P­{^1^H} experiments, results of syntheses with additional ligands (tris­(pentafluorophenyl)­phosphine and ethyl diphenylphosphinite), results of synthesis without any ligand, results of syntheses using presynthesized NiCl_2_L_2_ complexes as precursors, results of syntheses using ODE as a solvent instead of OLAM, results of syntheses where presynthesized Ni NCs are heated with the ligands (PDF)
