# Supplementary material for: Ligand Electronic Properties Dictate Composition of Ni-Based Nanocrystals
Source: J Am Chem Soc. 2025 Nov 14;147(47):43902–14. doi: 10.1021/jacs.5c15844 (PMC12673580; doi:10.1021/jacs.5c15844)
Supplement: Supplementary file 1 [file ja5c15844_si_001.pdf]

Ligand Electronic Properties Dictate Composition of Ni-Based Nanocrystals

Ludovic Zaza[1], Coline Boulanger[1], Krishna Kumar[1], Mikhail Agrachev[2], Aurélien Bornet[3], Jari Leemans[1], Gunnar Jeschke[2], Raffaella Buonsanti[1]*

[1] Laboratory of Nanochemistry for Energy (LNCE), Department of Chemical Sciences and Engineering, École Polytechnique Fédérale de Lausanne, CH-1950 Sion, Switzerland.

[2] Institute for Molecular Physical Science, Department of Chemistry and Applied Biosciences, ETH Zurich, Vladimir-Prelog-Weg 2, Zurich, Switzerland.

[3] Nuclear Magnetic Resonance Platform (NMRP), Department of Chemical Sciences and Engineering, Ecole Polytechnique Fédérale de Lausanne, CH-1950 Sion, Switzerland.

* raffaella.buonsanti@epfl.ch

Table of contents

Supplementary Note 1 – Literature survey on the synthesis of colloidal Ni-based NCs 5

Supplementary Fig. 1 – Color of the reaction solution before and after the formation of NCs 7

Supplementary Note 2 – Ligand steric and electronic properties 8

Supplementary Fig. 2 – Schematic representation of the Tolman cone angle and Tolman electronic parameter 8

Supplementary Tab. 1 – Cone angle and Tolman electronic parameter of the phosphines tested 10

Supplementary Fig. 3 – TEM and SAED of the Ni NCs obtained with ligands 3-8 12

Supplementary Fig. 4 – HRTEM images of the twinned Ni NCs obtained with ligand 1 13

Supplementary Fig. 5 – Low magnification TEM and HAADF-STEM images of the Ni NCs obtained with ligand 2 14

Supplementary Fig. 6 – HAADF-STEM-EDX elemental quantification and HRTEM analysis of the NCs obtained with ligand 9 15

Supplementary Fig. 7 – TEM, SAED, HAADF-STEM-EDX elemental quantification and HRTEM analysis of the NCs obtained with ligand 10 17

Supplementary Fig. 8 – TEM, SAED, HAADF-STEM-EDX elemental quantification and HRTEM analysis of the NCs obtained with ligand 11 19

Supplementary Fig. 9 – HAADF-STEM-EDX elemental quantification of the NCs obtained with ligand 12 21

Supplementary Fig. 10 – HAADF-STEM-EDX elemental quantification of the NCs obtained with ligand 14 22

Supplementary Fig. 11 – HAADF-STEM-EDX elemental quantification of the NCs obtained with ligand 15 23

Supplementary Fig. 12 – HRTEM analysis of the NCs obtained with ligand 15 24

Supplementary Fig. 13 – $^{31}P\{^{1}H\}$ NMR of ligands 10, 11 and 15 when they are heated 20 minutes at 250°C in OLAM 25

Supplementary Fig. 14 – Cu NCs obtained with ligands 10, 11, 12 and 15 during a 20-minute reaction at 270°C when $NiCl_2$ is substituted by CuBr 26

Supplementary Fig. 15 – TEM and HAADF-STEM-EDX elemental quantification of the NCs obtained with ligand 16 27

Supplementary Fig. 16 – TEM, SAED and HAADF-STEM-EDX elemental quantification of the NCs obtained with ligand 17 28

Supplementary Fig. 17 – UV-Vis spectroscopy of aliquots taken at different temperatures during Ni NC synthesis with ligand 3 30

Supplementary Fig. 18 – $^{31}P\{^1H\}$ NMR of aliquots taken during the synthesis of Ni NCs with ligand 1 31

Supplementary Fig. 19 – Color of the reaction solution when $NiCl_2$ is heated with ligands 1, 3 and 6 in ODE instead of OLAM 32

Supplementary Fig. 20 – $^{31}P\{^1H\}$ NMR of $NiCl_2(TOP)_2$ 33

Supplementary Fig. 21 – Color of the reaction solution when ligand 1 is heated 20 minutes at 250°C in ODE and OLAM 34

Supplementary Fig. 22 – Color of the reaction solution when ligand 1 is heated 20 minutes at 250°C in ODE and 1 ml OLAM is added 34

Supplementary Fig. 23 – TEM, SAED and HAADF-STEM-EDX of the control experiment when $NiCl_2$ is heated alone in OLAM during 20 minutes at 250°C 35

Supplementary Fig. 24 – ^1H NMR showing the role of OLAM as a reductant during the synthesis of Ni NCs with ligand 1 36

Supplementary Note 3 – Possible role of the ligands in the monomer formation 38

Supplementary Fig. 25 – $^{31}P\{^1H\}$ NMR of ligands 1, 3, 9 and 10 when they are mixed with $NiCl_2$ and CuBr in OLAM 40

Supplementary Fig. 26 – $^{31}P\{^1H\}$ NMR of ligand 1 when it is heated with $NiCl_2$ at 60°C, 120°C and 170°C in OLAM 41

Supplementary Fig. 27 – $^{31}P\{^1H\}$ NMR of ligands 3 and 10 when they are heated with $NiCl_2$ at 100°C and 150°C in OLAM 42

Supplementary Fig. 28 – ^1H NMR of a solution containing $NiCl_2$ and stoichiometric amount of OLAM 43

Supplementary Fig. 29 – ^1H NMR of the initial synthesis solution containing $NiCl_2$ and stoichiometric amount of ligands 1 and 3 in OLAM 44

Supplementary Fig. 30 – TEM showing precursor effects in the Ni NC synthesis 45

Supplementary Fig. 31 – Possible side reaction leading to Ph_2P-PPh_2 during the synthesis of Ni_xE_y NCs with ligand 10 46

Supplementary Note 4 – Complementary discussion on $^{31}P\{^1H\}$ NMR and ^1H-^{31}P HMBC during the synthesis of Ni_xE_y NCs with ligand 10 47

Supplementary Fig. 32 – $^{31}P\{^1H\}$ NMR of the aliquot taken at 240°C during the synthesis of Ni_xE_y NCs with ligand 10 and comparison with PClPh2 and PH_2Ph references 48

Supplementary Fig. 33 – Color of the reaction solution when $NiCl_2$ is heated 20 minutes at 250°C with various ligands in ODE instead of OLAM 49

Supplementary Fig. 34 – TEM and HAADF-STEM-EDX of the NCs obtained when ligand 11 is heated 20 minutes at 250°C with $NiCl_2$ in ODE 50

Supplementary Fig. 35 – TEM and HAADF-STEM-EDX of the NCs obtained when ligand 17 is heated 20 minutes at 250°C with $NiCl_2$ in ODE 51

Supplementary Fig. 36 – TEM of the NCs obtained when ligands 9, 10, 12 and 15 are heated 20 minutes at 250°C with $NiCl_2$ in ODE 52

Supplementary Fig. 37 – TEM and HAADF-STEM-EDX of the NCs obtained when ligand 10 is heated 20 minutes at 250°C with Ni NCs in OLAM 53

Supplementary Fig. 38 – TEM and HAADF-STEM-EDX of the NCs obtained when ligand 11 is heated 20 minutes at 250°C with Ni NCs in OLAM 54

Supplementary Fig. 39 – TEM and HAADF-STEM-EDX of the NCs obtained when ligand 12 is heated 20 minutes at 250°C with Ni NCs in OLAM 55

Supplementary Fig. 40 – TEM and HAADF-STEM-EDX of the NCs obtained when ligand 15 is heated 20 minutes at 250°C with Ni NCs in OLAM 56

Supplementary Fig. 41 – TEM and HAADF-STEM-EDX of the NCs obtained when ligand 17 is heated 20 minutes at 250°C with Ni NCs in OLAM 57

Supplementary Fig. 42 – TEM and SAED of the NCs obtained when ligand 1 is heated 20 minutes at 250°C with Ni NCs in OLAM 58

Supplementary Fig. 43 – TEM and SAED of the NCs obtained when ligand 2 is heated 20 minutes at 250°C with Ni NCs in OLAM 58

References 59

Supplementary Note 1: Literature survey on the synthesis of colloidal Ni-based NCs

Metallic Ni NCs. Ni nanorods are obtained through the decomposition of $Ni(COD)_2$ (COD = 1,5-cyclooctadiene) at 70°C in THF under an H_2 atmosphere with the addition of hexadecylamine (HDA).[1] Instead, Ni 2D nanoplates are obtained at 60°C in similar conditions with $(n\text{-}C_8H_{17})_4N^+(HOCH_2CO_2)^-$,[2] when nickel(II)acetylacetonate ($Ni(acac)_2$) is heated with $W(CO)_6$ at 160°C in oleylamine (OLAM),[3] or when $Ni(COOH)_2$ is heated with $Fe(CO)_5$, OLAM and oleic acid at 160°C in octadecene (ODE).[4] The product shifts to spherical Ni NCs by heating $Ni(acac)_2$ with trioctylphosphine (TOP) and OLAM in ODE at 220°C,[5] and Ni cubes are obtained by heating $Ni(acac)_2$ with TOP and HDA at 140°C in mesitylene under H_2 atmosphere.[6]

Ni phosphide NCs. Ni_2P nanowire NCs are obtained by heating $Ni(acac)_2$, oleic acid and TOP in trioctylamine at 300-320°C during 2-3h.[7,8] Spherical solid and hollow Ni_2P and $Ni_{12}P_5$ NCs are obtained by heating $Ni(acac)_2$ with TOP, triphenylphosphine or tributylphosphine in a mixture of OLAM/ODE or OLAM/octyl ether at 300-350°C for 1-3h.[9–11] Ni, Ni_2P or $Ni_{12}P_5$ NCs are obtained by heating $NiCl_2{\cdot}6H_2O$ or nickel(II) acetate ($Ni(OAc)_2$) 30 minutes with OLAM in ODE at 275°C with various organophosphite ligands ($P(OR)_3$) including $P(OMe)_3$, $P(OEt)_3$, $P(O^nBu)_3$, $P(OCH_2Bu)_3$, $P(O^iPr)_3$, $P(OPh)_3$, and $P(O\text{-}2,4\text{-}^tBu_2C_6H_4)_3$.[12] Ni_5P_4 NCs are synthesized by heating $Ni(acac)_2$ with TOP in OLAM at 350°C or in a mix of OLAM/ODE at 320°C for 1-2h.[13–15] NiP_2 NCs are obtained by heating $Ni(acac)_2$ with a large excess of TOP at 385°C in octyl ether with low amounts of OLAM during 24h.[16] Finally, amorphous Ni_xP_y NCs are also synthesized when the P:Ni(II) molar ratio is large. In particular, they are obtained when $Ni(acac)_2$ reacts with TOP in a mixture of OLAM/ODE at 215-240°C.[11,14] To the best of our knowledge, we are not aware of any colloidal synthesis of Ni_5P_2 NCs. Systematic variations of the synthetic parameters have revealed that the final nickel phosphide crystal phase depends on the Ni:P molar ratio, reaction time, reaction temperature and the amount of OLAM.[10,11,13,15]

Furthermore, studies have shown that transitions between different crystal phases may occur during the reaction.[10,12]

Ni arsenide NCs. To the best of our knowledge, there is only a single report on nickel arsenide NCs in which NiAs nanoplatelets NCs are synthesized by heating $NiCl_2$ with $As(NMe_2)_3$ in a mixture of ODE and TOP at 250°C.[17]

Ni antimonide NCs. To the best of our knowledge, there are only two reports on nickel antimonide NCs in which NiSb nanoparticles are obtained by heating $Ni(acac)_2$ and $SbPh_3$ in OLAM by using borane tert-butylamine complex as a reducing agent at 160°C.[18,19]

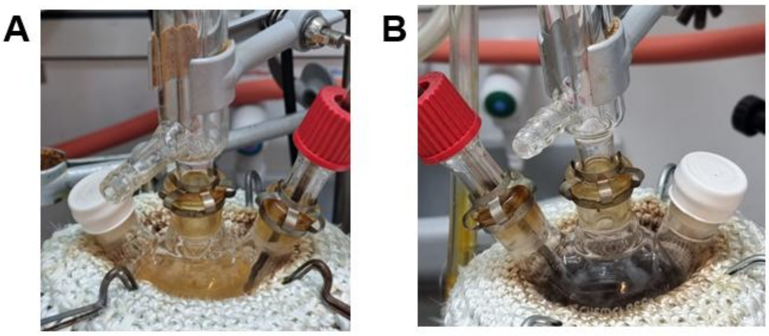

Figure S1. Typical color of the reaction solution (A) before and (B) after the formation of NCs.

Supplementary Note 2: Ligand steric and electronic properties

Phosphines (PR_3), arsines (AsR_3) and stibines (SbR_3) are neutral L-type ligands which donate 2 electrons to the metal center. They act both as σ-donors and π-acceptors with these properties largely depending on their electronic and steric properties.

The simplest and often used parameters to describe the size of these ligands is the cone angle which was introduced by Tolman in 1970.[20] The Tolman cone angle is defined as the "apex angle of a cylindrical cone, centered 2.28 Å away from the center of the P atom, which just touches the van der Waals radii of the outermost atoms of the model".[21] The 2.28 Å distance was chosen to represent the center distance between P and Ni atoms in NiL_4 complexes for which this descriptor was originally developed (**Figure S2A**).

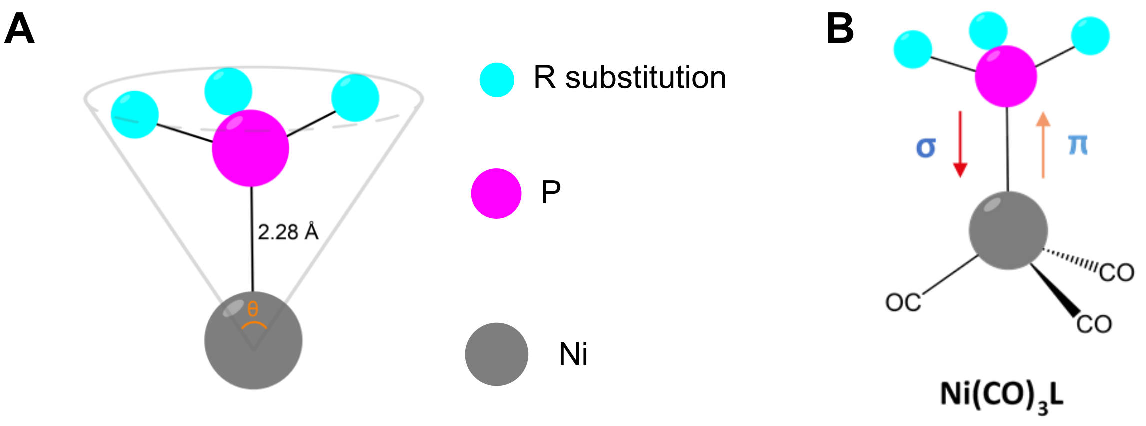

Figure S2. (A) Schematic representation of the Tolman cone angle. (B) Schematic representation of Tolman electronic parameter (TEP).

The electronic properties of these ligands have been extensively studied by using transition metal carbonyl complexes such as $Ni(CO)_3L$, $Cr(CO)_5L$, $Mo(CO)_5L$ or $W(CO)_5L$.[22] In these complexes, the position of the CO frequency mode in the IR-spectrum, ν(CO), depends on the metal electronic density.[22] The ligand acts as a σ-donor with its lone pair and increases the

electronic density of the metal, which can then be transferred back to the CO and ligand by π backbonding.[22] If the ligand is a poor π-acceptor, the density of π electrons available on the metal will increase for the CO ligands, resulting in a weakening of the C-O bonds and a lower ν(CO).[22] Instead, if the ligand is a good π-acceptor, ν(CO) will increase because the density of π electrons available on the metal for CO will decrease.[22] Historically, the classification was first developed on the $Ni(CO)_3L$ system by Tolman and is often referred to as the Tolman electronic parameter (TEP) (**Figure S2B**). More specifically, the TEP measures the frequency of the A_1 carbonyl mode of $Ni(CO)_3L$ in CH_2Cl_2.[21]

In homogeneous catalysis, the steric and electronic properties are primordial to understand the properties of many catalysts containing this type of ligands.[21,23,24] Subtle changes can greatly influence the reaction rate or the product distribution. As an illustrative example, changing the phosphine in Wilkinson's catalyst, $[RhCl(PPh_3)_3]$, greatly affects the reaction rate in alkene hydrogenation reactions. It was observed that for the hydrogenation of cyclohexene by $[RhCl(PR_3)_3]$, the rate increases in the order: R = p-C_6H_4F << R = Ph < R = p-$C_6H_4(OCH_3)$ indicating a clear electron density effects of the aryl substituent of the phosphine.[25] In particular, the electron rich phosphines favor the oxidative addition of H_2 in the cycle.[21] However, replacing the aryl group by an alkyl group with greater electron donating properties results in a decreasing hydrogenation rate following the order $PPh_3 > PEtPh_2 > PEt_2Ph > PEt_3$ because the dissociation rate of phosphine ligands from the $[H_2RhCl(PR_3)_3]$ complex is slower.[21,25]

Number	Name	Formula	Cone Angle (°)	TEP (cm^{-1})
1	Tri(phenyl)phosphine	PPh_3	145	2068.9
2	Tri(methyl)phosphine	PMe_3	118	2064.1
3	Tri(octyl)phosphine	$P(Oct)_3$	132[2]	2060.3[2]
4	Methyldiphenylphosphine	$PMePh_2$	136	2067
5	Tri(tertbutyl)phosphine	$P(^tBut)_3$	182	2056.1
6	Tri(cyclohexyl)phosphine	PCy_3	170	2058.3
7	Trimesitylphosphine	$P(Mes)_3$	212	2064.2[3]
8	Di(1-adamantyl)-n-butylphosphine (cataCXium A)	$P(Ad)_2Bu$	163[1,4]	2054.8[3,4]
9	Phenylphosphine	PH_2Ph	101	2077
10	Di(phenyl)phosphine	$PHPh_2$	128	2073.3
11	Di(isobutyl)phosphine	$PH(^iBut)_2$	124[1]	2066.8[3]
12	Triphenylarsine	$AsPh_3$	142[5]	2072[6]
13	Triethylarsine	$AsEt_3$	131[5]	2067[6]
14	Triphenylstibine	$SbPh_3$	142[5]	2074[6]
15	Diphenylphosphine oxide	$O{=}PHPh_2$	-	-

Table S1. Cone angle and TEP of the ligands tested in this study for the synthesis of NCs. Cone angle and TEP values are reported from ref [21]. [1]Estimated with Equation S1. [2]Approximated with P^nBu_3 value. [3]Estimated with Equation S2. [4]Data for PAd_3 from ref [26]. [5]Data from refs [27,28]. [6]Data from ref [29]. Schematic representations of the different ligands can be found in Figure 1A.

In the case of an asymmetric $PR_1R_2R_3$ phosphine where the substituents are different, the cone angle model uses the sum of half-angles. In this case, the cone angle can be estimated by taking 2/3 of the sum of the half cone angle of each substituent according to the following equation.

$$\theta = \frac{2}{3}\sum_{i=1}^{3}\frac{\theta_i}{2} \qquad (S1)$$

where θ_i is the cone angle of the ith phosphine in the tetrahedral triphosphine.

For example, for di(isobutyl)phosphine $PH(^iBu)_2$, the cone angle of the phosphine can be estimated as:

$$\theta = \frac{2}{3}\left(\frac{143}{2}+\frac{143}{2}+\frac{87}{2}\right) = 124°$$

where 143° is the cone angle for $P(^iBu)_3$ and 87° is the cone angle for PH_3.[21]

Similarly, the TEP of asymmetric phosphines can be estimated with the additivity of the substituent contributions χ_i according to the following equation.

$$v = 2056.1 + \sum_{i=1}^{3}\chi_i \qquad (S2)$$

For example, for di(isobutyl)phosphine $PH(^iBu)_2$, the TEP of the phosphine can be estimated as:

$$v = 2056.1 + 1.2 + 1.2 + 8.3 = 2066.8\ (cm^{-1})$$

where 1.2 is the substituent contribution for ^iBu and 8.3 is the substituent contribution for H.[21]

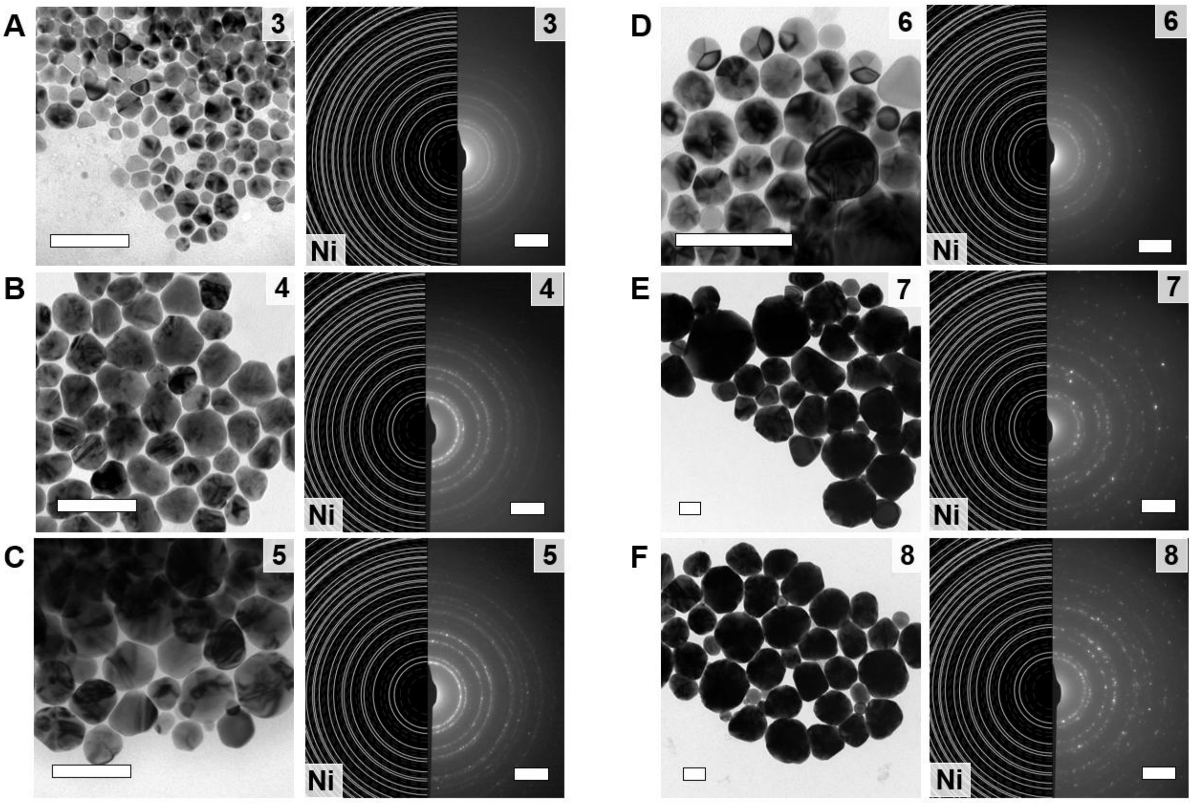

Figure S3. (A-F) Bright-field TEM image and SAED pattern of Ni NCs obtained with ligands 3-8. Scale bars are 100 nm for the TEM and 5 nm^{-1} for the SAED.

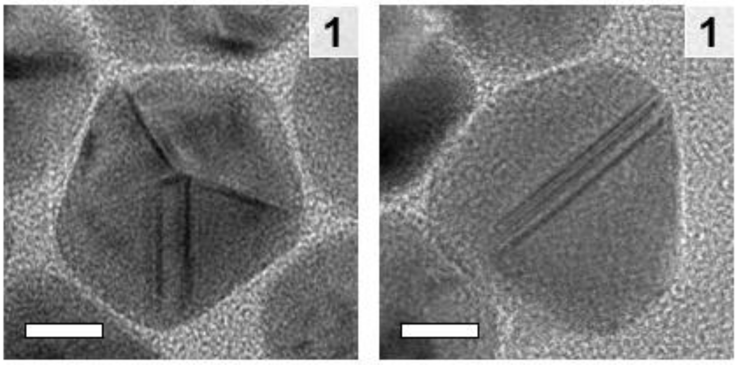

Figure S4. HR-TEM images of the Ni NCs obtained with ligand 1: on the left, a multiply twinned Ni decahedron and, on the right, a singly twinned Ni right bipyramid. The scale bars are 10 nm.

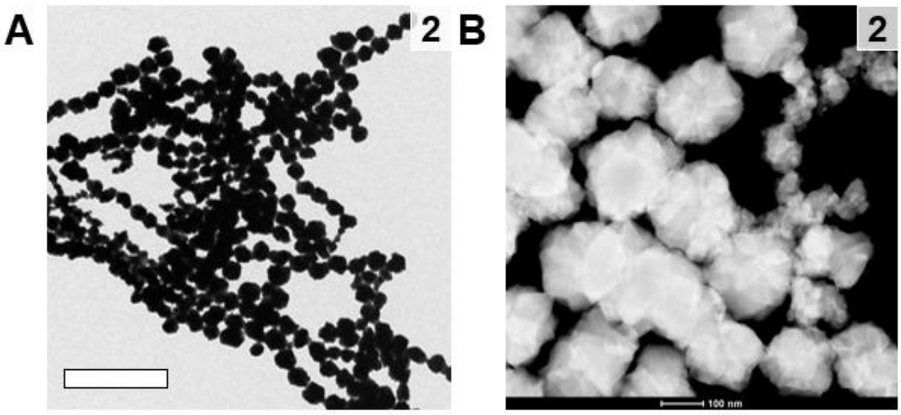

Figure S5. Low magnification bright-field TEM (A) and HAADF-STEM (B) images of the Ni NCs obtained with ligand 2 showing the ribbon-like structure adopted by the NCs. The scale bar is 500 nm for the TEM image.

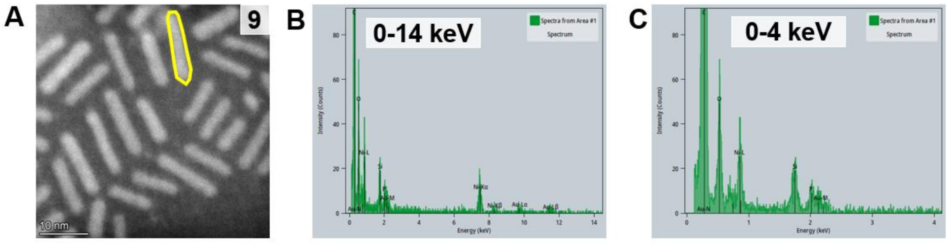

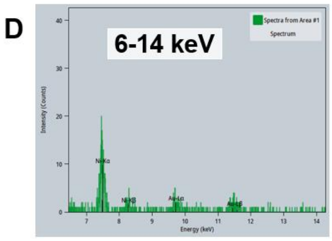

E

Element	Line	Atomic fraction (%)	Atomic error (%)	Mass fraction (%)	Mass error (%)	Fit error (%)
P	K	36.44	3.57	23.22	2.75	4.11
Ni	K	63.56	3.57	76.78	2.75	2.04

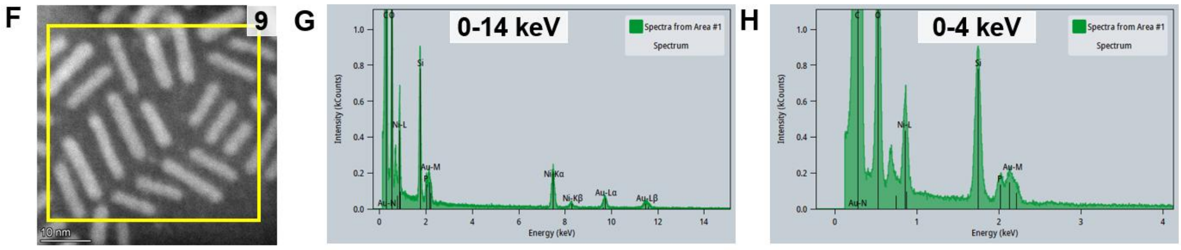

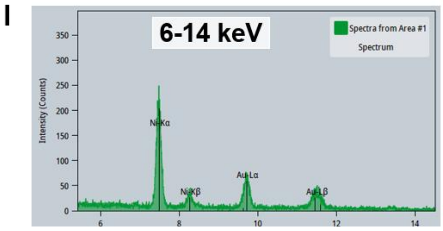

J

Element	Line	Atomic fraction (%)	Atomic error (%)	Mass fraction (%)	Mass error (%)	Fit error (%)
P	K	34.58	3.33	21.82	2.51	0.54
Ni	K	65.42	3.33	78.19	2.51	0.22

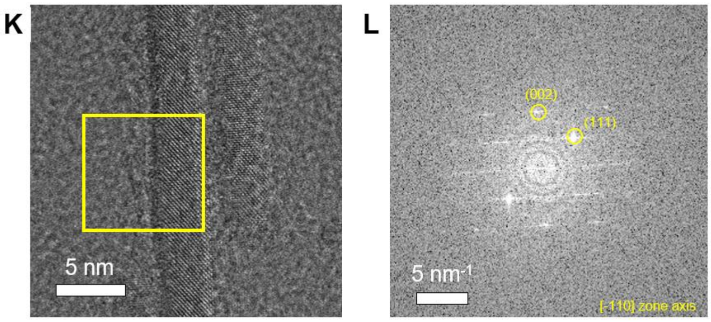

Figure S6. (A) HAADF-STEM image of Ni_2P NCs (same as Figure 3C) with the yellow line delimiting a single particle for STEM-EDX spectrum in B-D. (B-D) STEM-EDX spectrum of (A) ranging from 0 to 14 keV (B) with zooms in the 0-4 keV region (C) and in the 6-14 keV region (D). The gold signals come from the grid. (E) Quantitative Ni and P composition of the

NC using the Brown-Powell model as the ionization cross-section model. (F) HAADF-STEM image of Ni_2P NCs (same as in A and in Figure 3C) with the yellow line delimiting the region of interest for the STEM-EDX spectrum in G-I. (G-I) STEM-EDX spectrum of (F) ranging from 0 to 14 keV (G) with zooms in the 0-4 keV region (H) and in the 6-14 keV region (I). The gold signals come from the grid. (J) Quantitative Ni and P composition of the NC using the Brown-Powell model as the ionization cross-section model. (K) HR-TEM image of the NCs in (A) and (L) corresponding FFT of the NC highlighted in yellow in (K).

The SAED rings position and intensity suggest the composition of the NCs to be Ni_2P (**Figure 3B**). STEM-EDX indicates that one single NC is constituted of 63.6 at% of Ni and 36.4 at% of P (**Figure S6E**) while the composition averaged over few NCs indicates 65.4 at% of Ni and 34.6 at% of P (**Figure S6J**), which is consistent with the Ni_2P phase assigned from SAED. Indeed, the expected atomic composition for the Ni_2P phase is 66.7 at% Ni and 33.3 at% P. There are eight different intermetallic compounds that exist between Ni and P: Ni_3P, Ni_5P_2, $Ni_{12}P_5$, Ni_2P, NiP, NiP_2 and NiP_3.[30] Among these compounds, Ni_5P_2 (71.4 at% Ni and 28.6 at% P), $Ni_{12}P_5$ (70.6 at% Ni and 29.4 at% P) and Ni_2P (66.7 at% Ni and 33.3 at% P) all possess a close composition to the one found by STEM-EDX. To confirm the Ni_2P phase assigned to the NCs, we conducted HRTEM (**Figure S6K,L**). The diffraction patterns of a single NC (**Figure S6L**) perfectly match the expected Ni_2P phase.

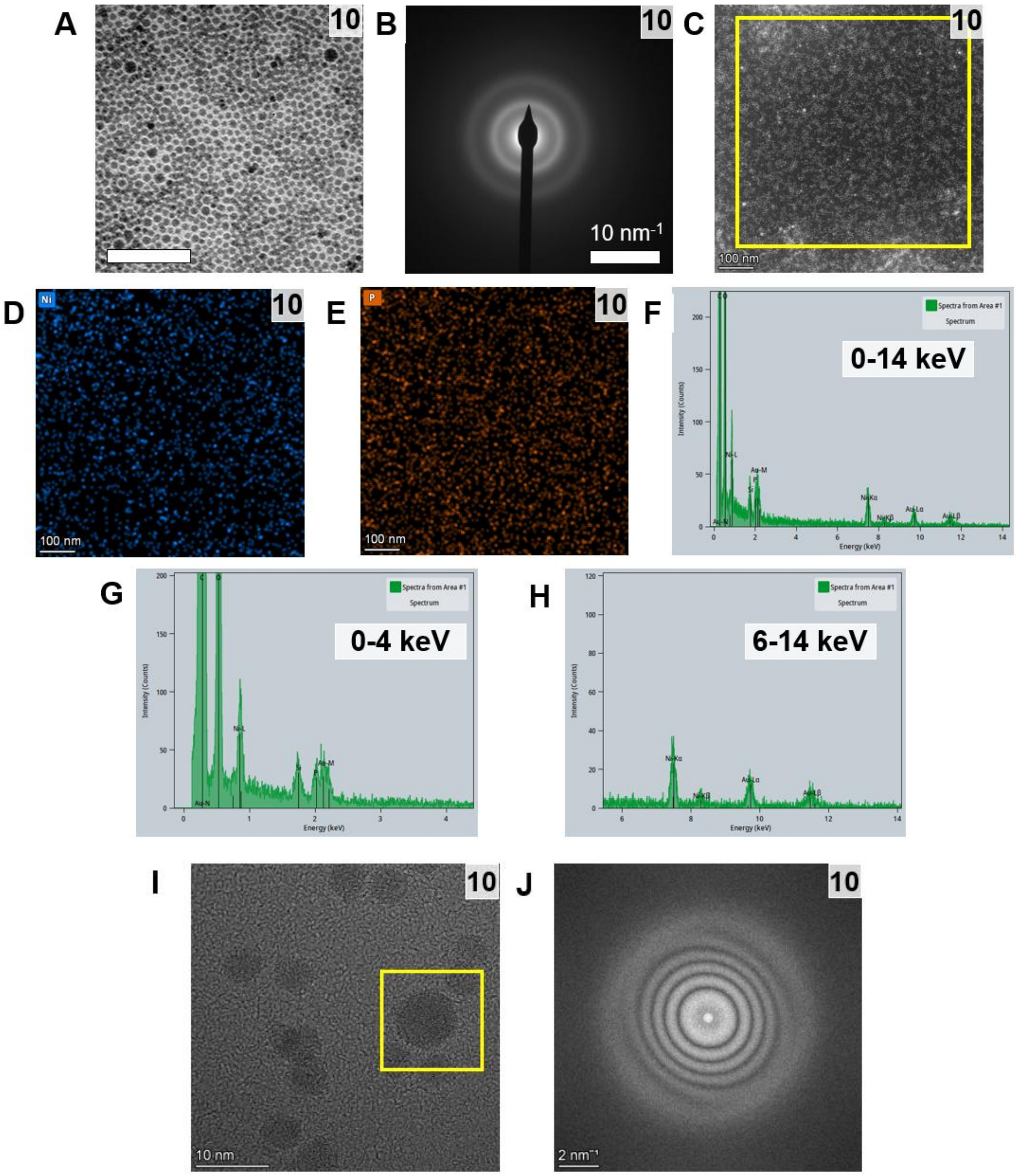

Figure S7. (A) Bright-field TEM image of the NCs obtained with ligand 10. Scale bar: 50 nm. (B) SAED of the NCs in (A). (C) HAADF-STEM image of the NCs with the yellow line delimiting the region of interest for the STEM-EDX spectrum. (D-E) STEM-EDX map of Ni

(D) and P (E). (F-H) STEM-EDX spectrum of (C) ranging from 0 to 14 keV (F) with zooms in the 0-4 keV region (G) and in the 6-14 keV region (H). The gold signals come from the grid. (I) HR-TEM image of the NCs in (A) and (J) corresponding FFT of the NC highlighted in yellow in (I).

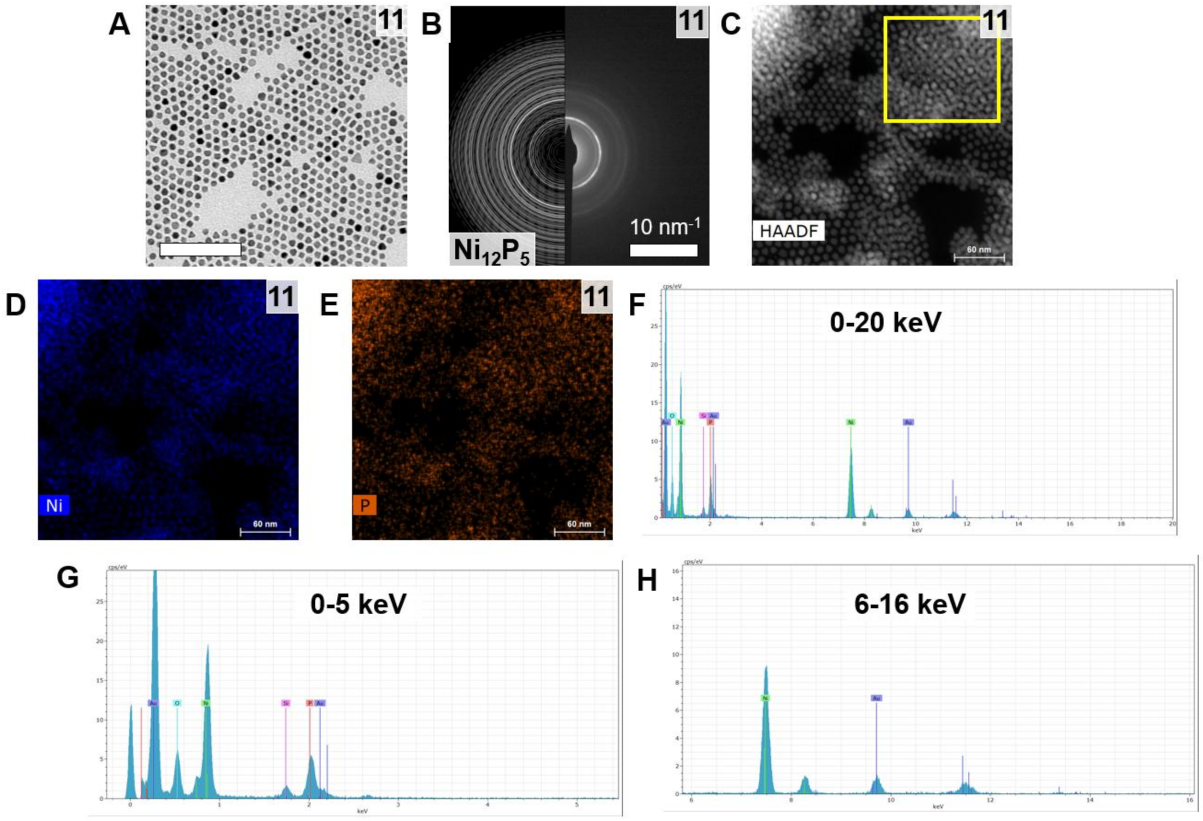

I

Element	Line	Atomic fraction (%)	Mass fraction (%)	Mass error (%)
P	K	29.79	18.29	0.69
Ni	K	70.21	81.71	2.66

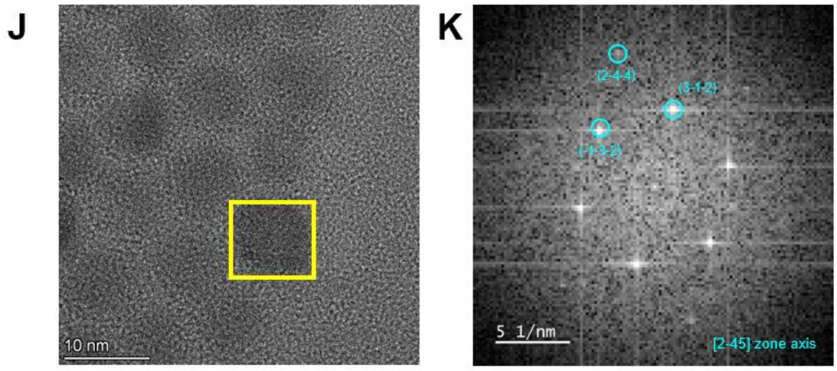

Figure S8. (A) Bright-field TEM image of the NCs obtained with ligand 11. Scale bar: 100 nm. (B) SAED of the NCs in (A) with $Ni_{12}P_5$ reference. (C) HAADF-STEM image of the NCs

with the yellow line delimiting the region of interest for the STEM-EDX spectrum. (D-E) STEM-EDX map of Ni (D) and P (E). (F-H) STEM-EDX spectrum of (C) ranging from 0 to 20 keV (F) with zooms in the 0-5 keV region (G) and in the 6-16 keV region (H). The gold signals come from the grid. (I). Quantitative Ni and P composition of the NC using the Cliff-Lorimer method. (J) HR-TEM image and corresponding FFT (K) of a single $Ni_{12}P_5$ NC.

The SAED rings position and intensity suggest the composition of the NCs to be $Ni_{12}P_5$ (**Figure S8B**). STEM-EDX indicates that the NCs are constituted of 70.2 at% of Ni and 29.8 at% of P (**Figure S8I**), which is consistent with the $Ni_{12}P_5$ phase assigned from SAED. Indeed, the expected atomic composition for the $Ni_{12}P_5$ phase is 70.6 at% Ni and 29.4 at% P. There are eight different intermetallic compounds that exist between Ni and P: Ni_3P, Ni_5P_2, $Ni_{12}P_5$, Ni_2P, NiP, NiP_2 and NiP_3.[30] Among these compounds, Ni_5P_2 (71.4 at% Ni and 28.6 at% P), $Ni_{12}P_5$ (70.6 at% Ni and 29.4 at% P) and Ni_2P (66.7 at% Ni and 33.3 at% P) all possess a close composition to the one found by STEM-EDX. To confirm the $Ni_{12}P_5$ phase assigned to the NCs, we conducted HRTEM (**Figure S8J**). The diffraction patterns of a single NC (**Figure S8K**) perfectly match the expected $Ni_{12}P_5$ phase.

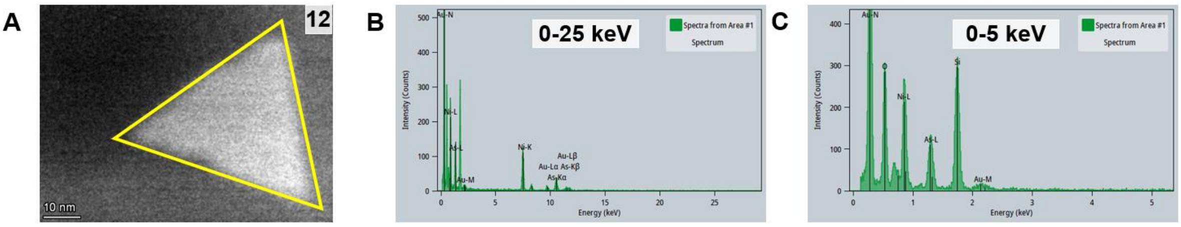

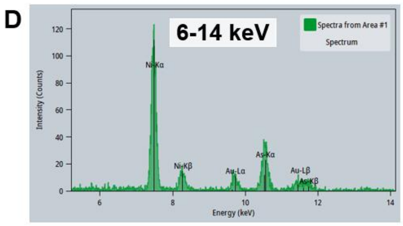

E

Element	Line	Atomic fraction (%)	Atomic error (%)	Mass fraction (%)	Mass error (%)	Fit error (%)
As	K	30.56	2.95	35.97	3.20	1.17
Ni	K	69.44	2.95	64.03	3.20	0.49

Figure S9. (A) HAADF-STEM image of an individual Ni_5As_2 NC (same as Figure 3H) with the yellow line delimiting the region of interest for STEM-EDX quantification. (B-D) STEM-EDX spectrum of (A) ranging from 0 to 25 keV (B) with zooms in the 0-5 keV region (C) and in the 6-14 keV region (D). The gold signals come from the grid. (E) Quantitative Ni and As composition of the NC using the Brown-Powell model as the ionization cross-section model.

The SAED rings position and intensity suggest the composition of the NCs to be Ni_5As_2 (**Figure 3G**). STEM-EDX indicates that one single NC is constituted of 69.4 at% of Ni and 30.6 at% of As (**Figure S9E**), which is consistent with the Ni_5As_2 phase assigned from SAED. Indeed, the expected atomic composition for the Ni_5As_2 phase is 71.4 at% Ni and 28.6 at% As. Furthermore, there only exist four intermetallic compounds between Ni and As: Ni_5As_2, $N_{11}As_8$, NiAs and $NiAs_2$.[31] Among these compounds, only Ni_5As_2 is consistent with the STEM-EDX quantification.

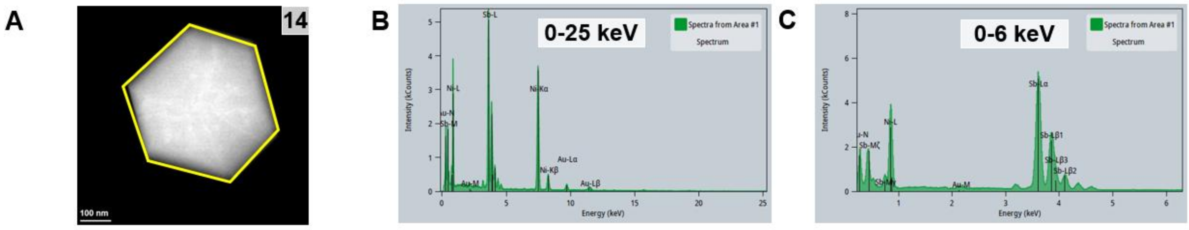

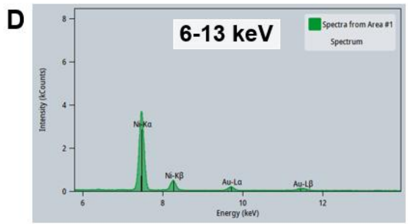

E

Element	Line	Atomic fraction (%)	Atomic error (%)	Mass fraction (%)	Mass error (%)	Fit error (%)
Sb	L	49.20	2.93	66.77	2.60	0.14
Ni	K	50.80	2.93	33.23	2.60	0.09

Figure S10. (A) HAADF-STEM image of an individual NiSb NC (same as Figure 3M) with the yellow line delimiting the region of interest for STEM-EDX quantification. (B-D) STEM-EDX spectrum of (A) ranging from 0 to 25 keV (B) with zooms in the 0-6 keV region (C) and in the 6-13 keV region (D). The gold signals come from the grid. (E) Quantitative Ni and Sb composition of the NC using the Brown-Powell model as the ionization cross-section model.

The SAED rings position and intensity suggest the composition of the NCs to be NiSb (**Figure 3L**). STEM-EDX indicates that one single NC is constituted of 50.8 at% of Ni and 49.2 at% of Sb (**Figure S10E**), which is consistent with the NiSb phase assigned from SAED. Indeed, the expected atomic composition for the NiSb phase is 50.0 at% Ni and 50.0 at% Sb. Furthermore, there only exist five intermetallic compounds between Ni and Sb: Ni_3Sb, Ni_5Sb_2, Ni_7Sb_3, NiSb and $NiSb_2$.[32] Among these compounds, only NiSb is consistent with the STEM-EDX quantification.

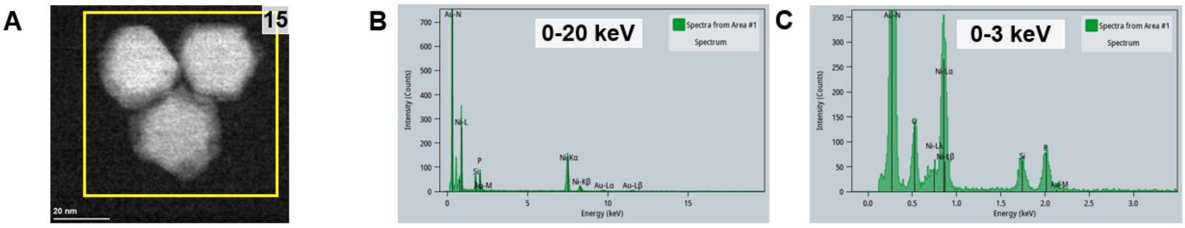

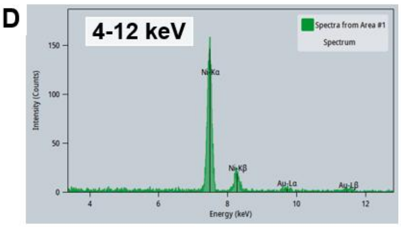

E

Element	Line	Atomic fraction (%)	Atomic error (%)	Mass fraction (%)	Mass error (%)	Fit error (%)
P	K	29.28	3.08	17.93	2.19	2.28
Ni	K	70.72	3.08	82.07	2.19	0.34

Figure S11. (A) HAADF-STEM image of three Ni_5P_2 NCs (same as Figure 3P) with the yellow line delimiting the region of interest for STEM-EDX quantification. (B-D) STEM-EDX spectrum of (A) ranging from 0 to 20 keV (B) with zooms in the 0-3 keV region (C) and in the 4-12 keV region (D). The gold signals come from the grid. (E) Quantitative Ni and P composition of the NC using the Brown-Powell model as the ionization cross-section model.

The SAED rings position and intensity suggest the composition of the NCs to be Ni_5P_2 (**Figure 3Q**). STEM-EDX indicates that one single NC is constituted of 70.7 at% of Ni and 29.3 at% of P (**Figure S11E**), which is consistent with the Ni_5P_2 phase assigned from SAED. Indeed, the expected atomic composition for the Ni_5P_2 phase is 71.4 at% Ni and 28.6 at% P. There are eight different intermetallic compounds that exist between Ni and P: Ni_3P, Ni_5P_2, $Ni_{12}P_5$, Ni_2P, NiP, NiP_2 and NiP_3.[30] Among these compounds, Ni_5P_2 (71.4 at% Ni and 28.6 at% P), $Ni_{12}P_5$ (70.6 at% Ni and 29.4 at% P) and Ni_2P (66.7 at% Ni and 33.3 at% P) all possess a close composition to the one found by STEM-EDX. To confirm the Ni_5P_2 phase assigned to the NCs, we conducted HRTEM (**Figure S12**).

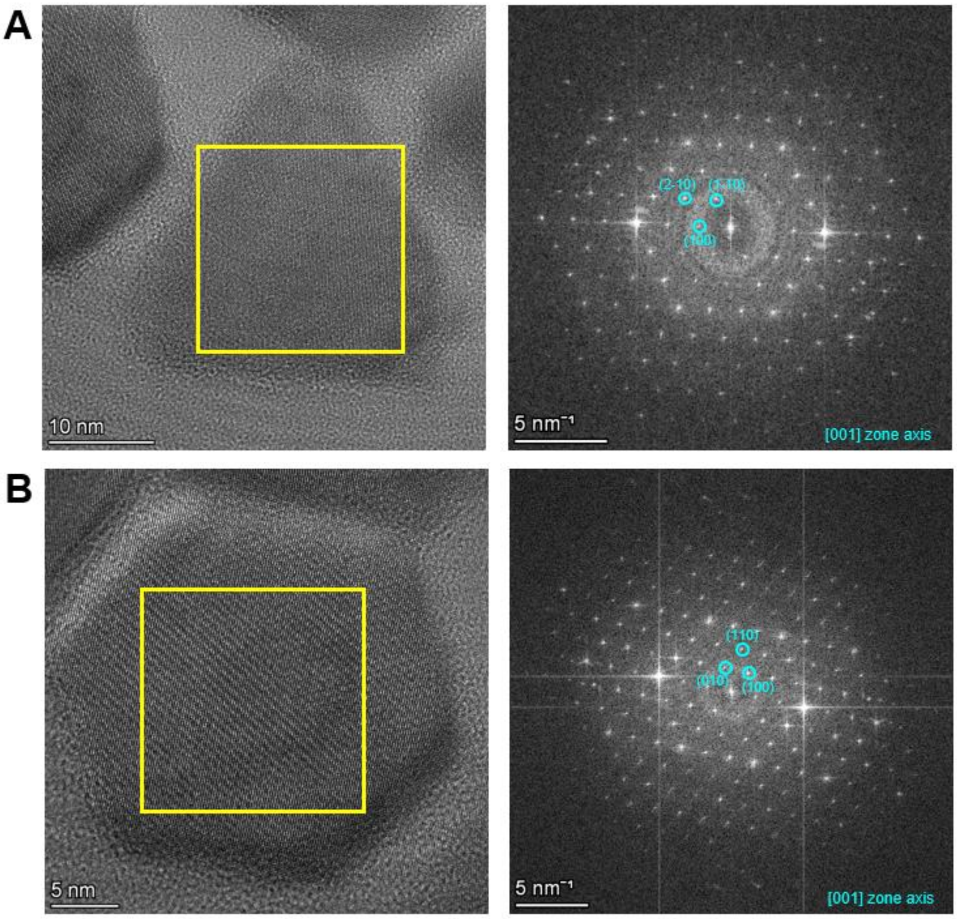

Figure S12. HR-TEM images and corresponding FFT of a single triangular NC (A) and of a single hexagonal NC (B) from Ni_5P_2 NCs (same as Figure 3P).

The diffraction patterns of a single triangular and hexagonal NC (respectively **Figure S12A** and **Figure S12B**) perfectly match the expected Ni_5P_2 phase.

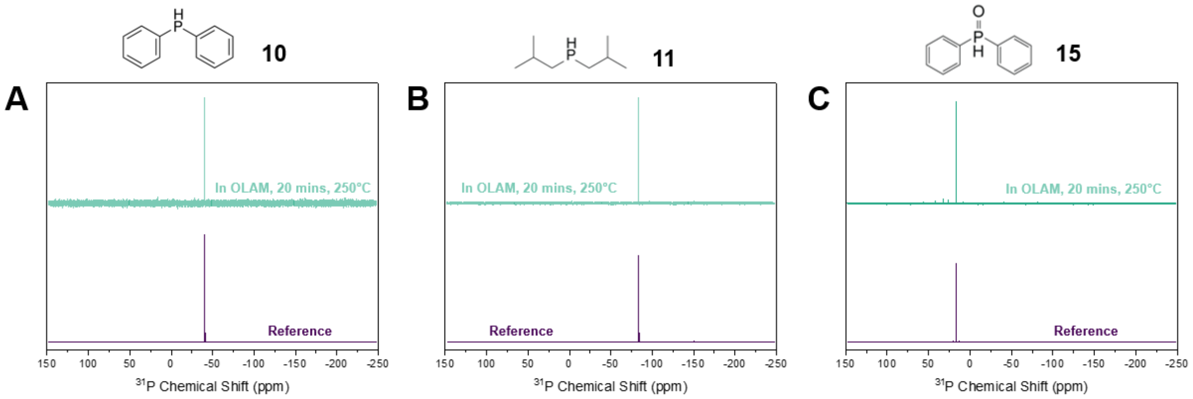

Figure S13. $^{31}P\{^{1}H\}$ NMR spectrum of ligands 10 (A), 11 (B) and 15 (C) when they are heated in OLAM without $NiCl_2$ in the same conditions than the NC synthesis (i.e. during 20 minutes at 250°C) with the phosphine reference. Solvent is toluene-d8 in every experiment.

These ligands form Ni phosphide NCs with $NiCl_2$ (**Figure 3** and **Figures S7, S8, S11, S12**). Here, ^{31}P NMR indicates that these ligands do not undergo significant thermal degradation when they are heated in OLAM for 20 minutes at 250°C, which is the synthesis temperature. This result rules out the possibility that the ligands form nickel phosphide through thermal decomposition.

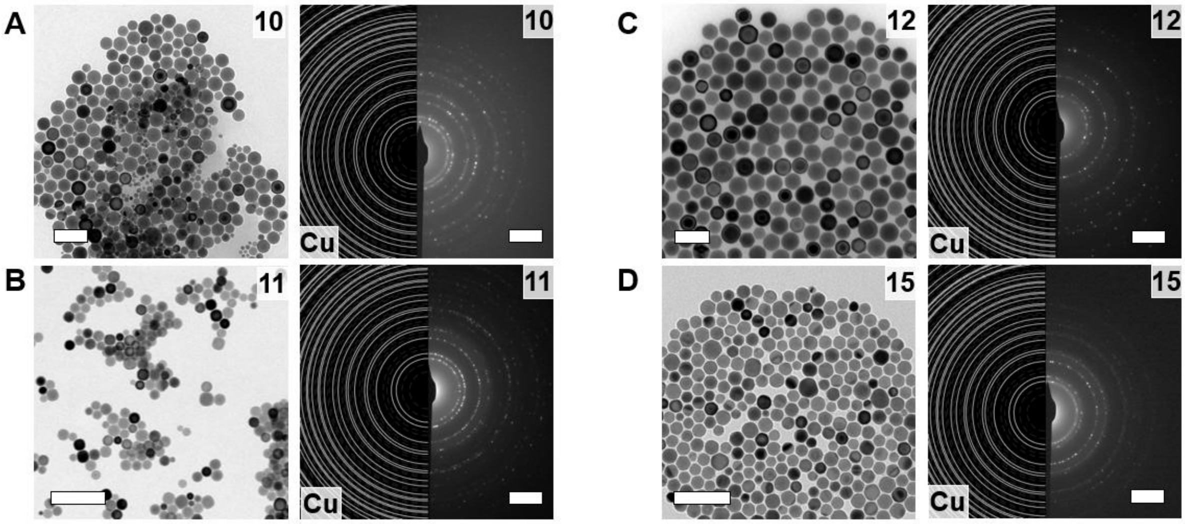

Figure S14. (A-D) Bright-field TEM image and SAED of the Cu NCs obtained with various ligands leading to the formation of nickel phosphide and arsenide in our study. The Cu precursor is CuBr, the reaction temperature is 270°C and the reaction time is 20 minutes like in our study. Scale bars are 100 nm for the TEM and 5 nm^{-1} for the SAED.

A

Number	Name	Formula	Cone Angle (°)	TEP (cm-1)
1	Tri(phenyl)phosphine	PPh_3	145	2068.9
16	Tris(pentafluorophenyl)phosphine	$P(C_6F5)_3$	184	2090.9

B

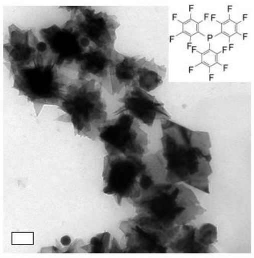

C D E

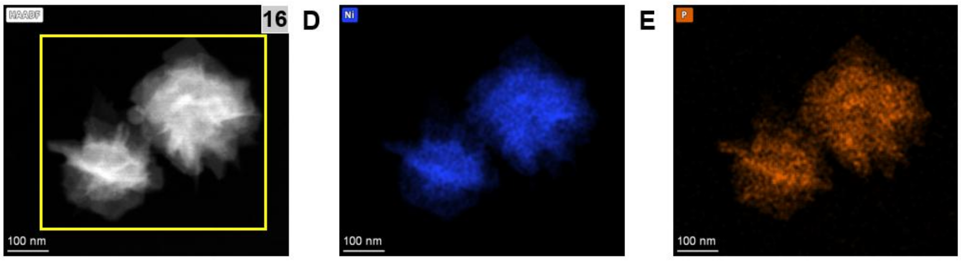

F G H

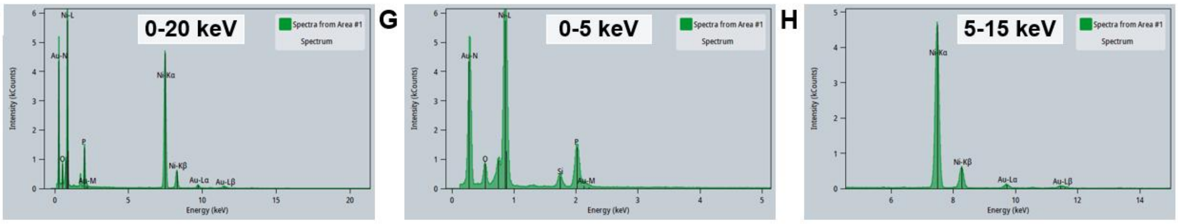

I

Element	Line	Atomic fraction (%)	Atomic error (%)	Mass fraction (%)	Mass error (%)	Fit error (%)
P	K	19.46	2.30	11.31	1.47	0.07
Ni	K	80.54	2.30	88.69	1.47	0.04

Figure S15. (A) Comparison of the steric and electronic properties of $P(C_6H_5)_3$ and $P(C_6F_5)_3$. Cone angle and TEP values are reported from ref [21]. (B) Bright-field TEM image of the NCs obtained with $P(C_6F_5)_3$ as a ligand. The scale bar is 100 nm. In the top right corner: schematic representation of the $P(C_6F_5)_3$ ligand. (C) HAADF-STEM image of two NCs from (B) with the yellow line delimiting the region of interest for the STEM-EDX spectrum. (D-E) STEM-EDX map of Ni (D) and P (E). (F-H) STEM-EDX spectrum of (C) ranging from 0 to 20 keV (F) with zooms in the 0-5 keV region (G) and in the 5-15 keV region (H). The gold signals come from the grid. (I) Quantitative Ni and P composition of the NC using the Brown-Powell model as the ionization cross-section model.

A

Number	Name	Formula	Cone Angle (°)	TEP (cm^{-1})
1	Tri(phenyl)phosphine	PPh_3	145	2068.9
17	Ethyl diphenylphosphinite	$P(OEt)Ph_2$	133	2071.6

B

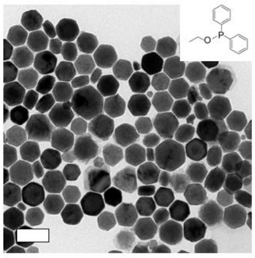

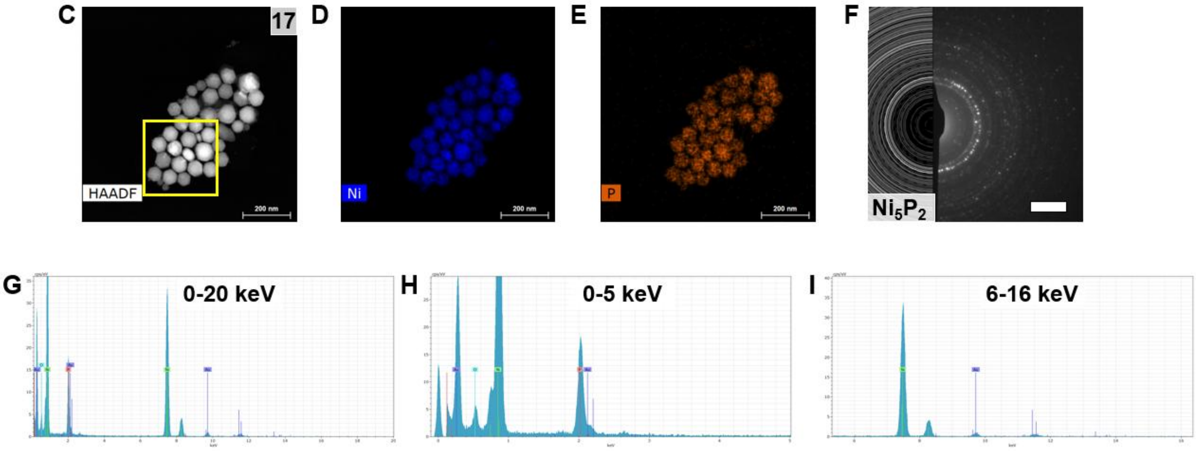

J

Element	Line	Atomic fraction (%)	Mass fraction (%)	Mass error (%)
P	K	26.24	15.81	0.56
Ni	K	73.76	84.19	2.65

Figure S16. (A) Comparison of the steric and electronic properties of PPh_3 and $P(OEt)Ph_2$. Cone angle and TEP values are reported from ref [21]. (B) Bright-field TEM image of the NCs obtained with $P(OEt)Ph_2$ as a ligand. The scale bar is 100 nm. In the top right corner: schematic representation of the $P(OEt)Ph_2$ ligand. (C) HAADF-STEM image of NCs from (B) with the yellow line delimiting the region of interest for the STEM-EDX spectrum. (D-E) STEM-EDX map of Ni (D) and P (E). (F) SAED of the NCs from (B) with Ni_5P_2 reference. Scale bar is 10 nm^{-1}. (G-I) STEM-EDX spectrum of (C) ranging from 0 to 20 keV (G) with zooms in the 0-5 keV region (H) and in the 6-16 keV region (I). The gold signals come from the grid. (J). Quantitative Ni and P composition of the NC using the Cliff-Lorimer method.

The SAED rings position and intensity suggest the composition of the NCs to be Ni_5P_2 (**Figure S16F**). STEM-EDX indicates that the NCs are constituted of 73.8 at% of Ni and 26.2 at% of P (**Figure S16J**), which is consistent with the Ni_5P_2 phase assigned from SAED. Indeed, the expected atomic composition for the Ni_5P_2 phase is 71.4 at% Ni and 28.4 at% P and is the closest phase with respect to the at% from STEM-EDX.

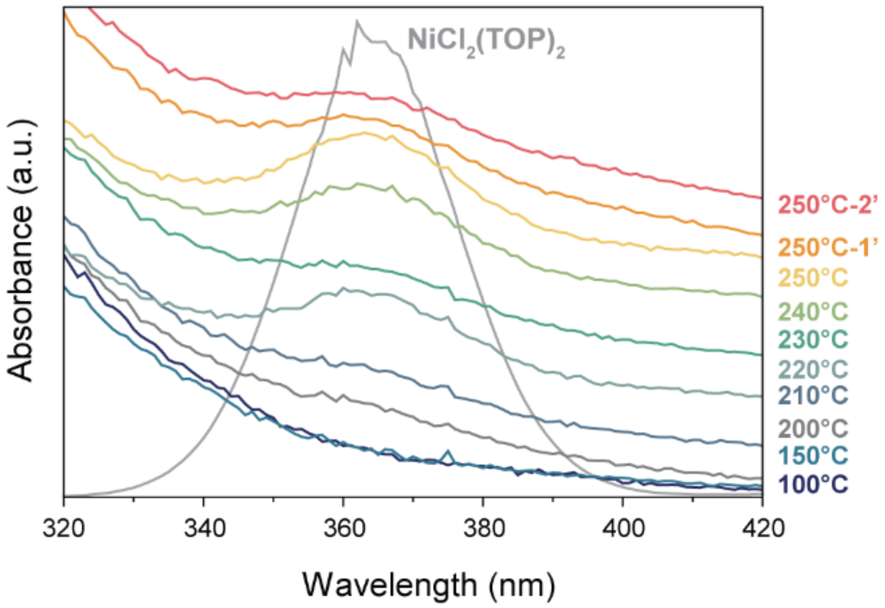

Figure S17. UV-Vis spectroscopy of the aliquots taken during the synthesis of Ni NCs with TOP (3) and comparison with the $NiCl_2(TOP)_2$ reference.

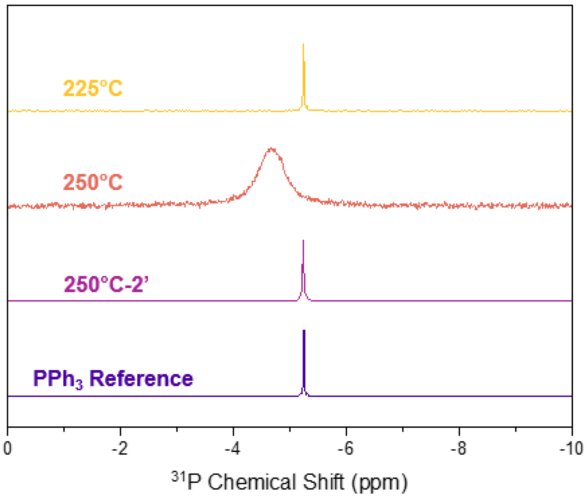

Figure S18. $^{31}P\{^{1}H\}$ NMR spectrum of aliquots taken during the synthesis of Ni NCs with PPh_3 (ligand 1) at 225°C, 250°C and after 2 minutes at 250°C. There is a significant shift and broadening of the ^{31}P resonance at 250°C.

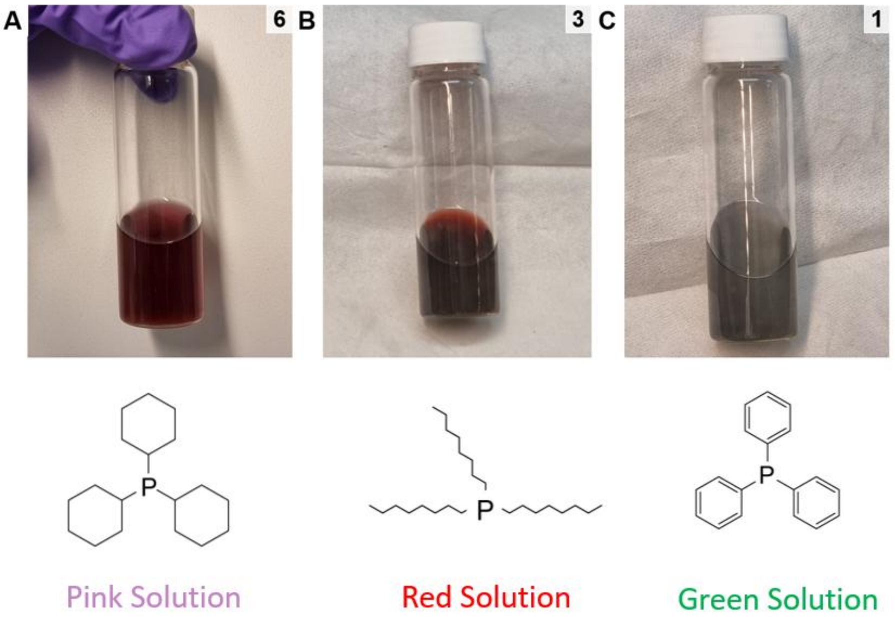

Figure S19. Color of the solutions obtained when $NiCl_2$ is heated at 250°C during 20 minutes in ODE with (A) ligand 6 (PCy_3) and (B) ligand 3 (TOP). (C) Color of the solution obtained with ligand 1 (TPP) when the reaction time is increased to 120 minutes.

The different colors perfectly match those reported for $NiCl_2L_2$ molecular complexes, i.e. pink for $NiCl_2(PCy_3)_2$, red for $NiCl_2(P^nBu_3)_2$ and green for $NiCl_2(PPh_3)_2$.[33,34] The formation of the complexes (i.e. the color change of the solution) occurs around 215°C for ligand PCy_3 and TOP and after 30 minutes at 250°C for PPh_3 (**Figure S19**). This observation can be explained because PPh_3 is a weaker σ-donor than PCy_3 and TOP due to the aryl substitution. As a comparison, the TEP of PPh_3 is 2068.3 cm^{-1} whereas it is 2060.3 cm^{-1} and 2058.3 cm^{-1} for TOP and PCy_3 respectively (**Table S1**).[21]

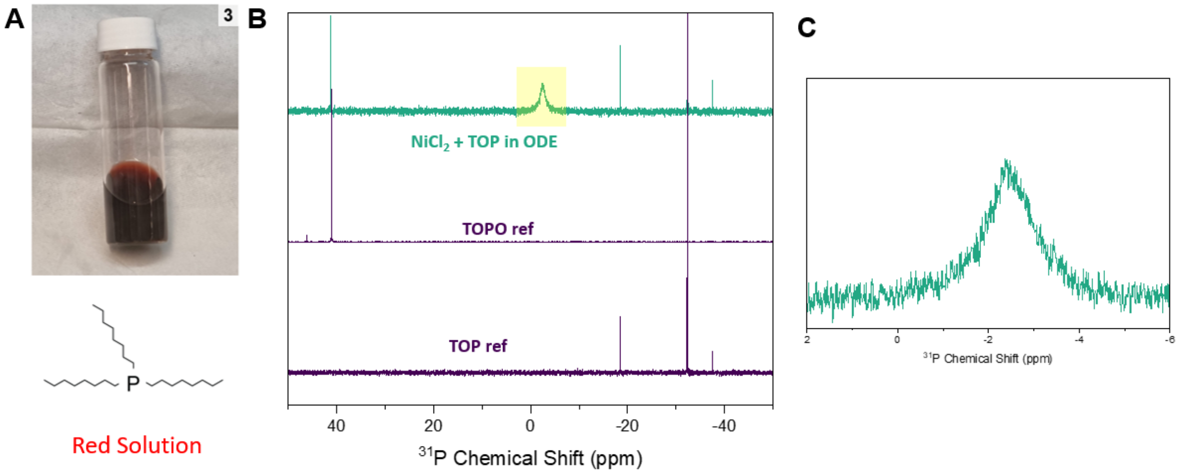

Figure S20. $^{31}P\{^1H\}$ NMR spectrum of the $NiCl_2(TOP)_2$ solution obtained by heating TOP with $NiCl_2$ at 250°C during 20 minutes in ODE (B in Figure S19). (A) Picture of the solution (same as Figure S19B). (B) $^{31}P\{^1H\}$ NMR spectrum of the solution (top, in green), with reference for TOPO (middle, purple) and TOP (bottom, purple). (C) Magnified spectrum of the region around -2 ppm highlighted in yellow in (B).

The $^{31}P\{^1H\}$ NMR spectrum shows resonances at 40 ppm and -2 ppm in addition to the TOP reference at -32 ppm (**Figure S20B**, top). The peak at 40 ppm can be attributed to TOPO, the oxidation product of TOP. This oxidation is unintended. The new resonance at -2 ppm is displayed in **Figure S20C**. The broadening of the peak and downfield shift compared to the free TOP is consistent with the formation of a $NiCl_2(TOP)_2$ complex.

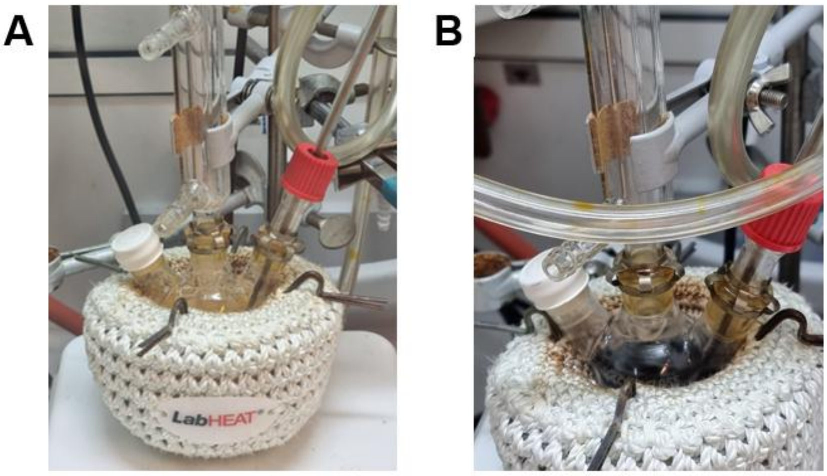

Figure S21. (A) Color of the solution after a 20-minute reaction at 250°C with $NiCl_2$ and PPh_3 in ODE. (B) Color of the solution in the same reaction conditions when ODE is substituted with OLAM.

The yellow color of the solution obtained in ODE (**Figure S21A**) indicates that no NC forms, thus highlighting the central role of OLAM for obtaining Ni NCs. Furthermore, the solution instantaneously becomes dark when 1 ml of OLAM is added (**Figure S22**), thus highlighting the critical role of OLAM.

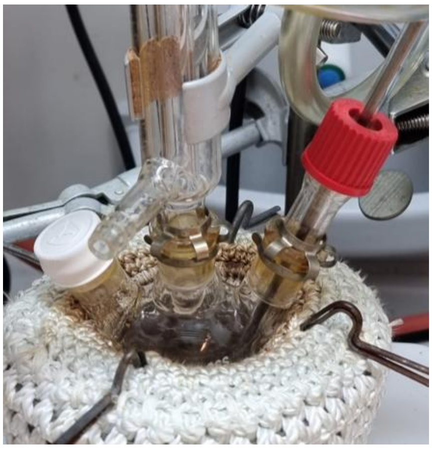

Figure S22. Color of the solution with $NiCl_2$ and PPh_3 in ODE (same as in Figure S21A) after the quick addition of 1 ml of OLAM.

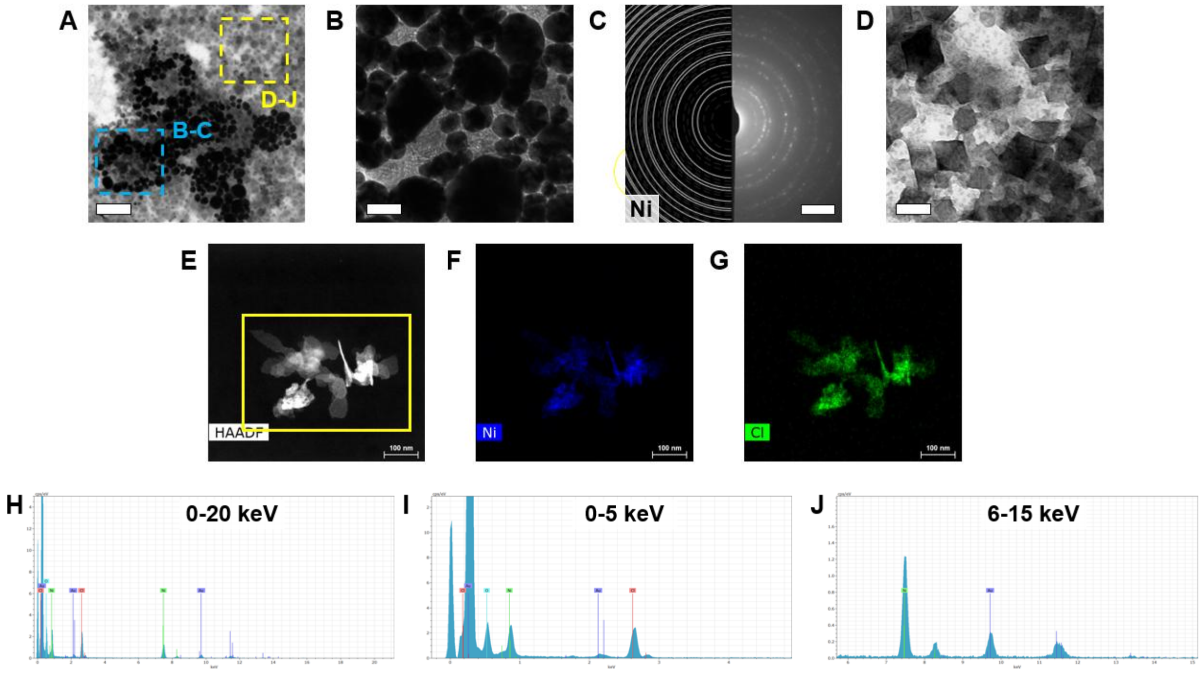

Figure S23. (A) Bight field TEM image of the product of the reaction when $NiCl_2$ is heated at 250°C for 20 minutes without any ligand. Scale bar: 500 nm. (B) TEM image of Ni NCs aggregates formed (highlighted in blue in (A)). Scale bar: 100 nm. (C) SAED of the Ni aggregates from (B) with Ni reference. Scale bar: 10 nm^{-1}. (D) TEM of unreacted $NiCl_2$ (highlighted in yellow in (A)). (E) HAADF-STEM image of unreacted $NiCl_2$ from (D) with the yellow line delimiting the region of interest for the STEM-EDX spectrum. (F-G) STEM-EDX map of Ni (F) and P (G). (H-J) STEM-EDX spectrum of (E) ranging from 0 to 20 keV (H) with zooms in the 0-5 keV region (I) and in the 5-15 keV region (J). The gold signals come from the grid.

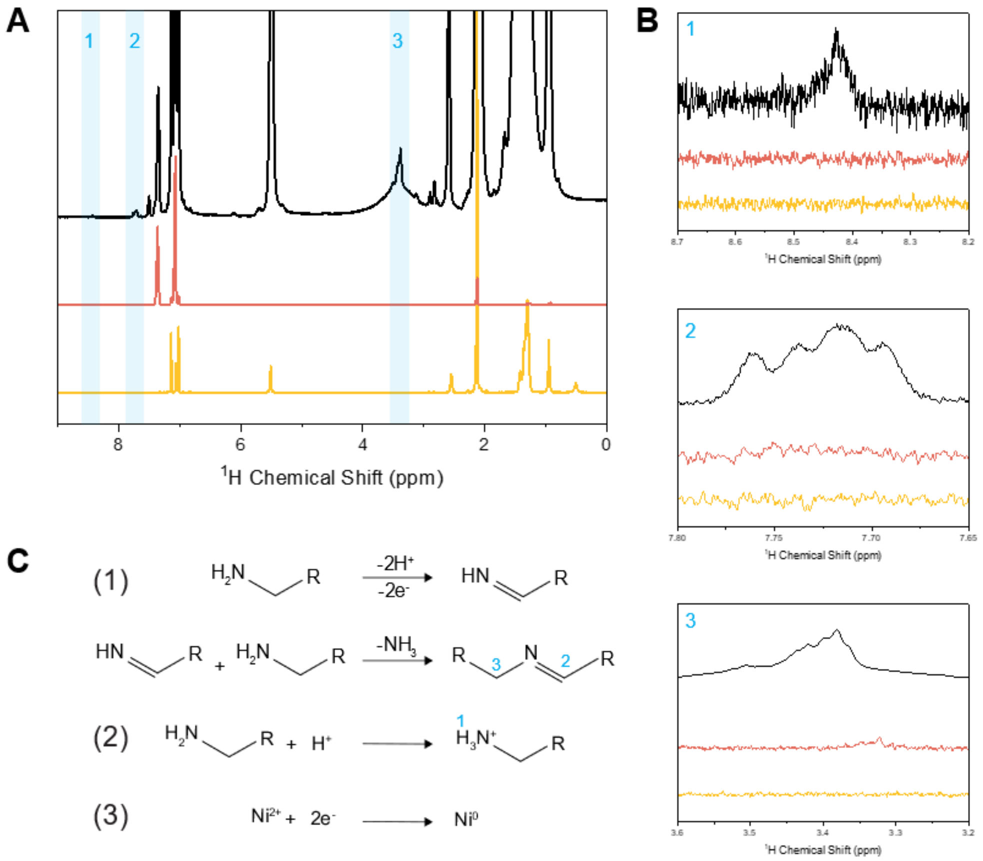

Figure S24. The role of OLAM as a reductant in the syntheses leading to Ni NCs**.** (A) ^{1}H NMR spectra of the reaction solution after the synthesis of twinned Ni NCs with PPh_3 (ligand 1) (black line) with reference spectra for PPh_3 (red line) and OLAM (orange line). Solvent is toluene-d8 in every experiment. (B) Zoom in regions 1, 2 and 3 in (A). Protons 1 can be assigned to oleylammonium[35] while protons 2 and 3 can be assigned to the aldimine formed from OLAM (see (C)).[36–38] (C) Chemical reaction leading to the formation of (1) an aldimine and highlighting the role of OLAM as a reducing agent, (2) oleylammonium and (3) metallic nickel. The protons 1, 2 and 3 are highlighted in the corresponding molecules.

^{1}H NMR reveals new resonances at 3.4 ppm, 7.7 ppm and 8.4 ppm with PPh_3 (**Figure S24A,B**) and at 2.6 ppm, 3.5 ppm, 7.5 ppm and 8.3 ppm with TOP (**Figure 5D**). The resonances at 3.4 ppm and 7.7 ppm are consistent with the formation of a secondary aldimine, which results from

the reaction of the imine formed after the oxidation of OLAM with another OLAM molecule (**Figure S24C**).[36–38] We assign the new broad resonance at 8.4 ppm to oleylammonium protons,[35] which are consistent with the formation of H^+ during the oxidation of OLAM into an imine in the first reaction step (**Figure S24C**).

Supplementary Note 3: Possible role of the ligands in the monomer formation

$^{31}P\{^{1}H\}$ NMR does not provide direct evidence for the formation of $NiCl_2L_2$ molecular complexes in the reaction solution prior to NC formation (**Figures S25-27**). The phosphorus signals perfectly match the free uncoordinated reference for aliquots collected at low temperatures (i.e. 60°C, **Figure S25A**) and at higher temperatures closer to NC formation (i.e. 120-170°C, **Figure S26** and **Figure S27**), which were measured at room temperature.

The formation of these complexes usually requires heating and time because the soft Lewis bases ER_3 ligands have a limited affinity towards the borderline Lewis acid Ni(II) centers. In strong contrast, the ligand coordination is almost instantaneous at low temperature when $NiCl_2$ is substituted by a softer Lewis acid metallic center, such as Cu(I) in CuBr (**Figure S25B**). These results suggest that the ligands only coordinate with the $NiCl_2$ centers to form $NiCl_2L_2$ complexes at elevated temperatures.

When we used pre-synthesized $NiCl_2L_2$ complexes as precursors, we obtained the same final NC product as in our reactions (**Figure S30**). Similar Ni NCs are obtained when $NiCl_2(PPh_3)_2$ is heated in OLAM and when $NiCl_2$ and PPh_3 are heated separately in OLAM (**Figure S30A**). The result is similar for $NiCl_2(PMe_3)_2$ and $NiCl_2$ and PMe_3 (**Figure S30B**).

In addition to the ligands, OLAM is present in a large excess in the reaction environment and contains amino groups which are harder Lewis bases than the ER_3 ligands. Thus, the formation of $NiCl_2(OLAM)_x$ complexes must be considered. Such complexes have been reported with x = 1, 2 or 6 with NH_3.[41] In fact, ^{1}H NMR spectroscopy suggests the possible formation of a coordinating complex when OLAM is mixed with $NiCl_2$ in stoichiometric amount at low temperatures (**Figure S28**). Unfortunately, the presence of these complexes in the starting solution is difficult to assess due to the large excess of OLAM with respect to $NiCl_2$ (**Figure S29**). In addition, $NiCl_2$ exhibits relatively poor solubility in OLAM. This observation implies

that, if $NiCl_2(OLAM)_x$ species are indeed formed, their concentration in solution is likely to be low.

Overall, these observations highlight that OLAM does not play a major role in the initial stages of the synthesis with respect to monomer formation and that $NiCl_2L_2$ may only form at elevated temperatures. **Figure S30** shows that $NiCl_2L_2$ molecular precursors lead to the same Ni NCs as in our synthesis, indicating their likely involvement in the reaction.

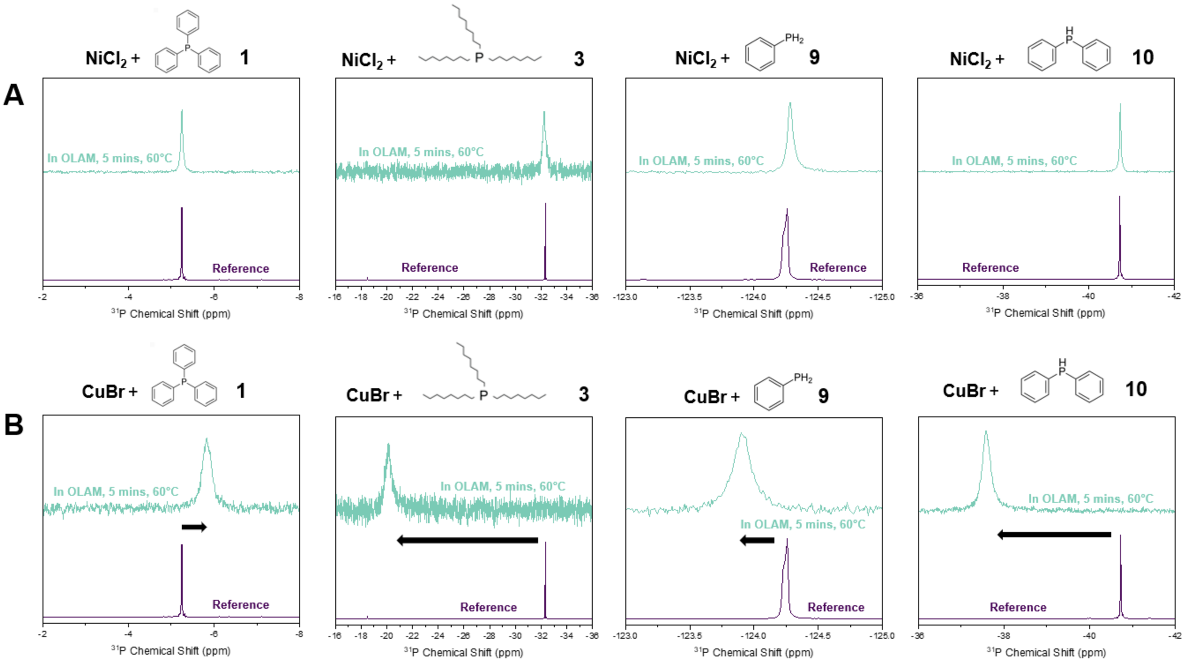

Figure S25. ${}^{31}P\{{}^{1}H\}$ NMR spectrum of ligands 1, 3, 9 and 10 in OLAM mixed during 5 minutes at 60°C with $NiCl_2$ (A), and CuBr (B). Solvent is toluene-d8 in every experiment.

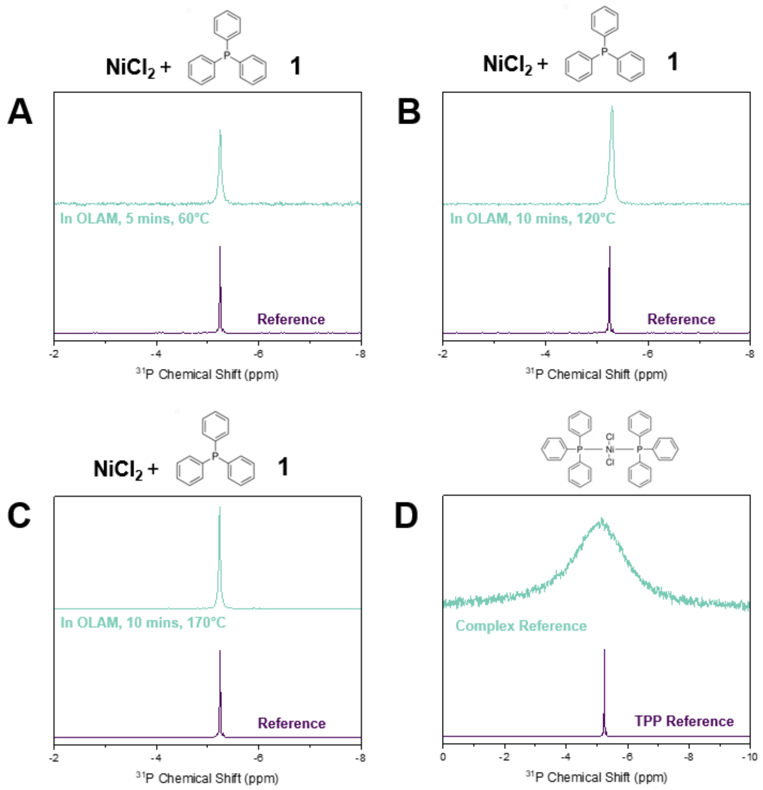

Figure S26. $^{31}P\{^{1}H\}$ NMR spectrum when ligand 1 is heated in OLAM with $NiCl_2$ during 5 minutes at 60°C (same as in Figure S17) (A), during 10 minutes at 120°C (B), and during 10 minutes at 170°C (C). (D) $^{31}P\{^{1}H\}$ NMR spectrum reference of the preformed commercial $NiCl_2(PPh_3)_2$ complex. Solvent is toluene-d8 in every experiment.

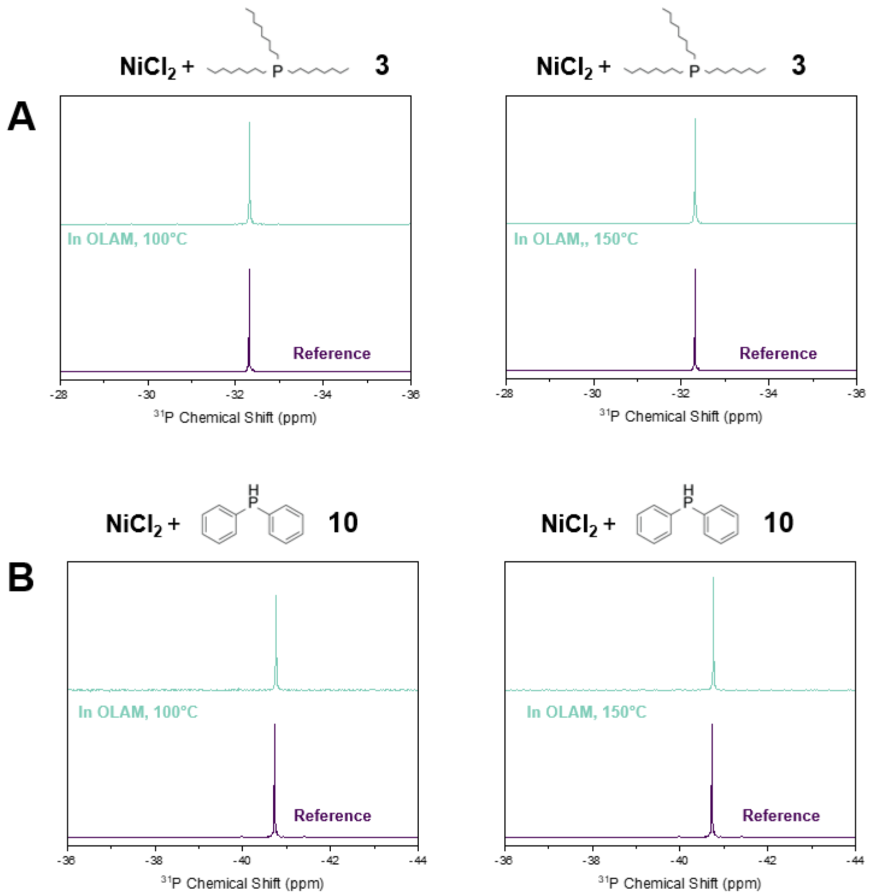

Figure S27. $^{31}P\{^{1}H\}$ NMR spectrum of aliquots taken during NC synthesis when ligands 3 (in A) and 10 (in B) are heated in OLAM with $NiCl_2$ at 100°C and 150°C. Solvent is toluene-d8 in every experiment.

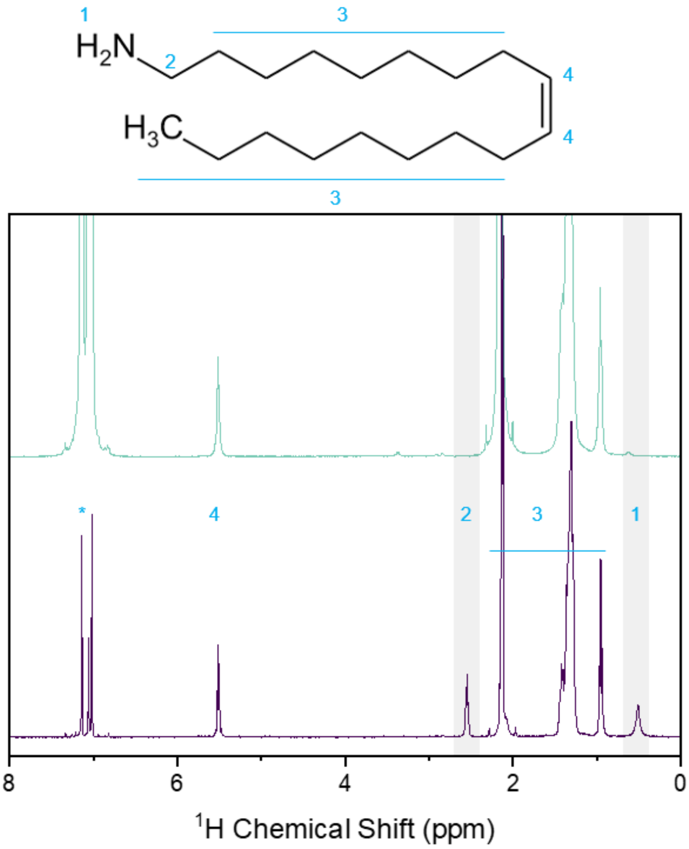

Figure S28. ^{1}H NMR spectrum of a solution containing $NiCl_2$ and two equivalents of OLAM mixed in toluene for 5 minutes at 60°C (top, in green) with OLAM reference (bottom, in purple). Solvent is toluene-d8. The OLAM protons are assigned with numbers. The protons highlighted with (*) come from toluene.

To investigate the possible formation of a complex between $NiCl_2$ and OLAM, we mixed OLAM with $NiCl_2$ in stoichiometric amounts (with 2:1 OLAM:$NiCl_2$) in toluene for 5 minutes at 60°C. In the presence of $NiCl_2$, the $C\underline{H_2}NH_2$ resonance (at 2.5 ppm, protons 2) and the $N\underline{H_2}$ resonance (at 0.5 ppm, protons 1) of OLAM disappear. The disappearance of the resonances is most likely due to broadening of the signals, caused by the interaction between OLAM and $NiCl_2$, thus indicating the formation of a molecular complex.

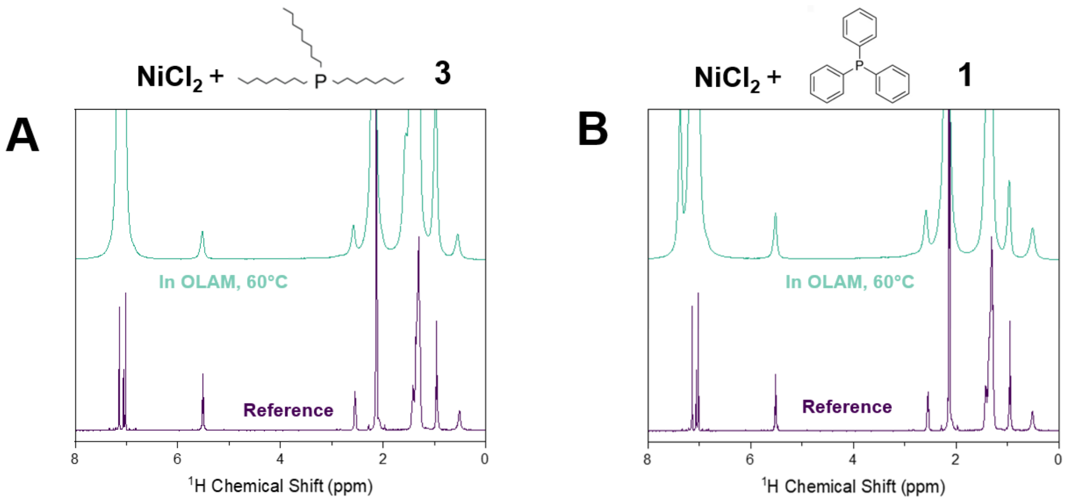

Figure S29. ^{1}H NMR spectrum of the synthesis starting solution heated at 60°C followed by dilution in toluene. The spectra show the solutions containing $NiCl_2$, two equivalents of the ligand and OLAM diluted in toluene (top, in green) and OLAM reference (bottom, in purple). Solvent is toluene-d8.

In contrast to **Figure S28** where OLAM was mixed to $NiCl_2$ in stoichiometric amounts, the ^{1}H NMR of the starting solutions shown in **Figure S29** do not feature the disappearance of the OLAM resonances. In those solutions, OLAM is present in much larger excess (with ~100:1 OLAM:$NiCl_2$ vs. 2:1 OLAM:$NiCl_2$ in **Figure S28**) resulting in the presence of free uncoordinated OLAM for which we observe the C$\underline{H_2}$$NH_2$ resonances at 2.5 ppm and the N$\underline{H_2}$ resonance at 0.5 ppm.

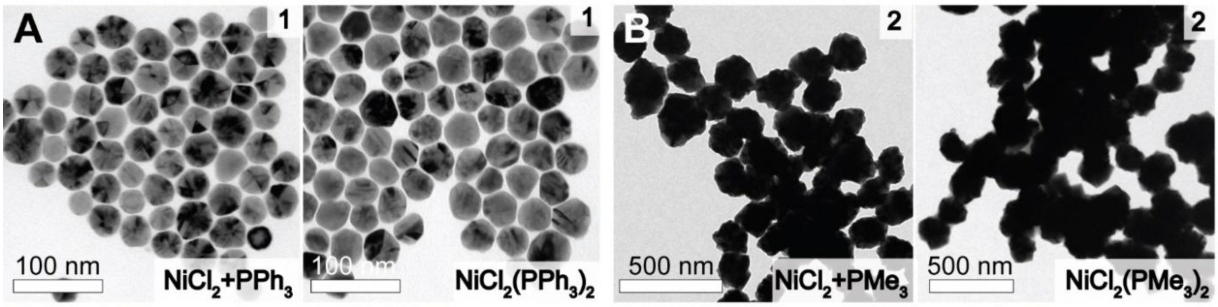

Figure S30. Bright field TEM images of the Ni NCs obtained using (A) $NiCl_2$ and PPh_3 (left) and the pre-synthesized $NiCl_2(PPh_3)_2$ complex (right) and (B) $NiCl_2$ and PMe_3 (left) and the pre-synthesized $NiCl_2(PMe_3)_2$ complex (right).

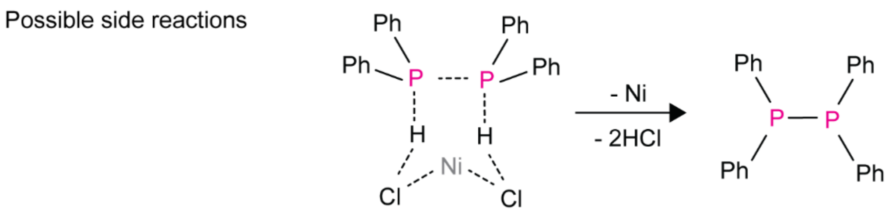

Figure S31. Possible side reaction leading to the formation of Ph_2P-PPh_2 by oxidative coupling of $PHPh_2$ with $NiCl_2$.

Supplementary Note 4: Complementary discussion on $^{31}P\{^{1}H\}$ NMR and ^{1}H-^{31}P HMBC during the synthesis of Ni_xE_y NCs with $PHPh_2$

^{1}H-^{31}P HMBC indicates that the phosphorus signal at 26.8 ppm is coupled to highly deshielded protons at 8 ppm (**Figure 6A**, panel 3 and **Figure 6B**) and that the signal at 41.3 ppm is coupled to aromatic protons and to shielded protons at 2.8 ppm and 1.7 ppm (**Figure 6A**, panel 2 and **Figure 6B**). In both cases, the ^{31}P signals are not coupled to any doublet in the 4.5 - 6.5 ppm region of the ^{1}H spectrum, suggesting that these molecules do not contain a P-H bond anymore, for which coupling between the hydrogen and the phosphorus is expected. The presence of upfield proton for the 41.3 ppm resonance suggests the presence of an alkyl substitution, possibly in a molecule of the type $PPh_2(NH\text{-}C_{18}H_{35})$ where one OLAM molecule has reacted with $PHPh_2$. The phosphorus resonance at 26.8 ppm might come from some tetraphenyl phosphonium species ($^{+}PPh_4$) for which ^{31}P resonances are expected around 25 ppm.[42,43] They also typically possess deshielded aromatic protons in the range of 7.7-7.9 ppm.[42] The formation of these species is possible by Ni salts catalysis.[44]

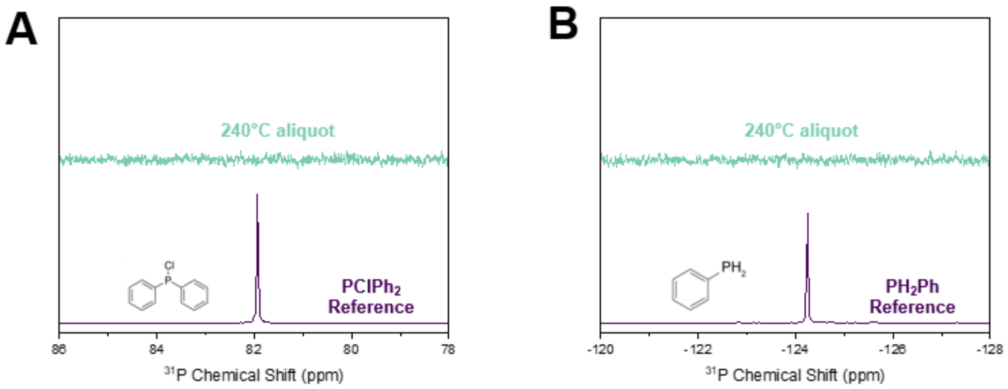

Figure S32. $^{31}P\{^1H\}$ NMR spectrum of the synthesis aliquot taken at 240°C during the synthesis of nickel phosphide NCs with $PHPh_2$ (ligand 10) and comparison with $PClPh_2$ reference (A) and PH_2Ph reference (B). Solvent is toluene-d8 in every experiment.

The two spectra in **Figure S32** clearly show that neither $PClPh_2$ nor PH_2Ph are created during the reaction, thus highlighting that extra Cl and H atoms are not accepted by the ligand.

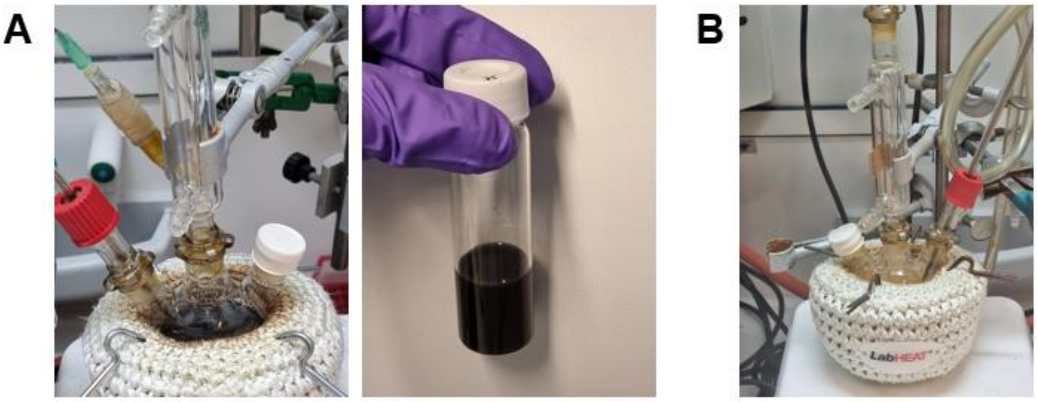

Figure S33. (A) Color of the solution obtained when ligands in Figures S34-36 react with $NiCl_2$ in ODE for 20 minutes at 250°C (here: with ligand 11). (B) Color of the solution when $NiCl_2$ is heated alone in ODE for 20 minutes at 250°C.

The reaction in ODE between $NiCl_2$ and the different ligands shown in **Figures S34-36** is evidenced visually by the formation of a dark reaction solution when the temperature reaches ~250°C (**Figure S33A**). Instead, no color change is observed when $NiCl_2$ is heated alone in the same conditions (**Figure S33B**). In comparison, the ligands yielding Ni NCs form colored solutions of $NiCl_2L_2$ complexes in the same conditions (**Figure S19**). We note that different reaction times are required for the solution to become dark depending on which ligand is used. For example, the solution instantaneously becomes dark with ligand 17, but such color change requires 3-4 minutes with ligand 15. Finally, the solution is only slightly darker after 20 minutes with ligand 12. This observation indicates that the ligands react at different rates with the $NiCl_2$ precursor.

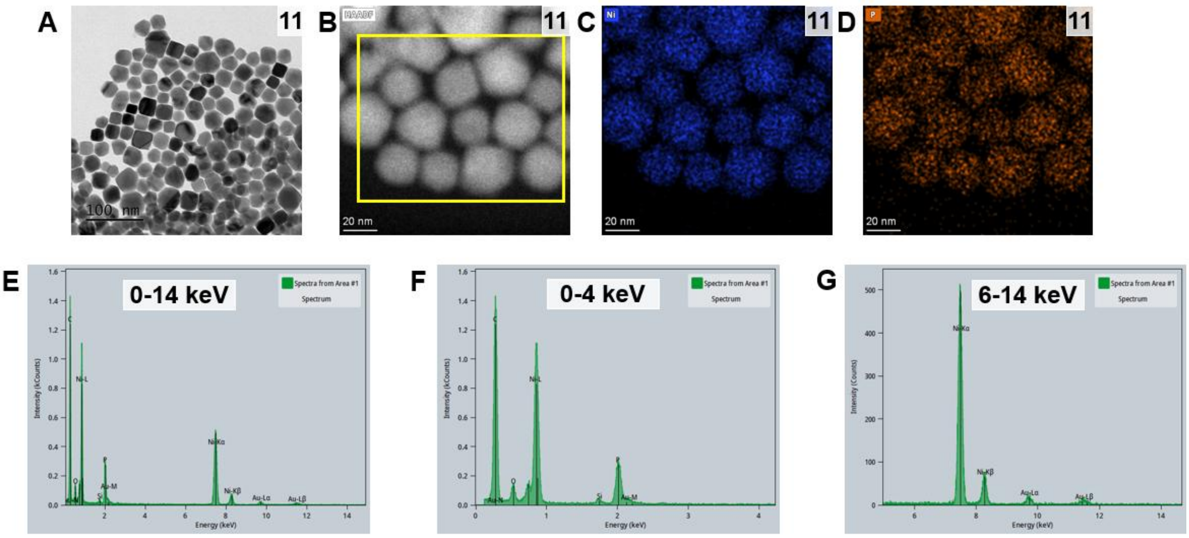

Figure S34. (A) Bright-field TEM image of the NCs obtained when ligand 11 reacts with the $NiCl_2$ precursor in octadecene (ODE) during a 20-minute reaction at 250°C. (B) HAADF-STEM image of NCs from (A) with the yellow line delimiting the region of interest for the STEM-EDX spectrum. (C-D) STEM-EDX map of Ni (C) and P (D). (E-G) STEM-EDX spectrum of (B) ranging from 0 to 14 keV (E) with zooms in the 0-4 keV region (F) and in the 6-14 keV region (G). The gold signals come from the grid.

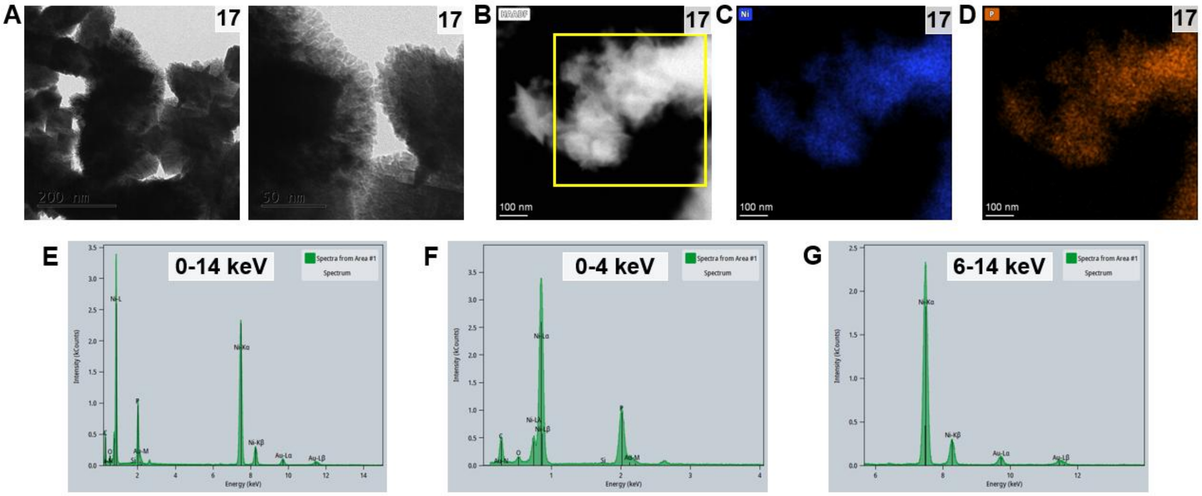

Figure S35. (A) Bright-field TEM images of the NCs obtained when ligand 17 reacts with the $NiCl_2$ precursor in ODE during a 20-minute reaction at 250°C. (B) HAADF-STEM image of NCs from (A) with the yellow line delimiting the region of interest for the STEM-EDX spectrum. (C-D) STEM-EDX map of Ni (C) and P (D). (E-G) STEM-EDX spectrum of (B) ranging from 0 to 14 keV (E) with zooms in the 0-4 keV region (F) and in the 6-14 keV region (G). The gold signals come from the grid.

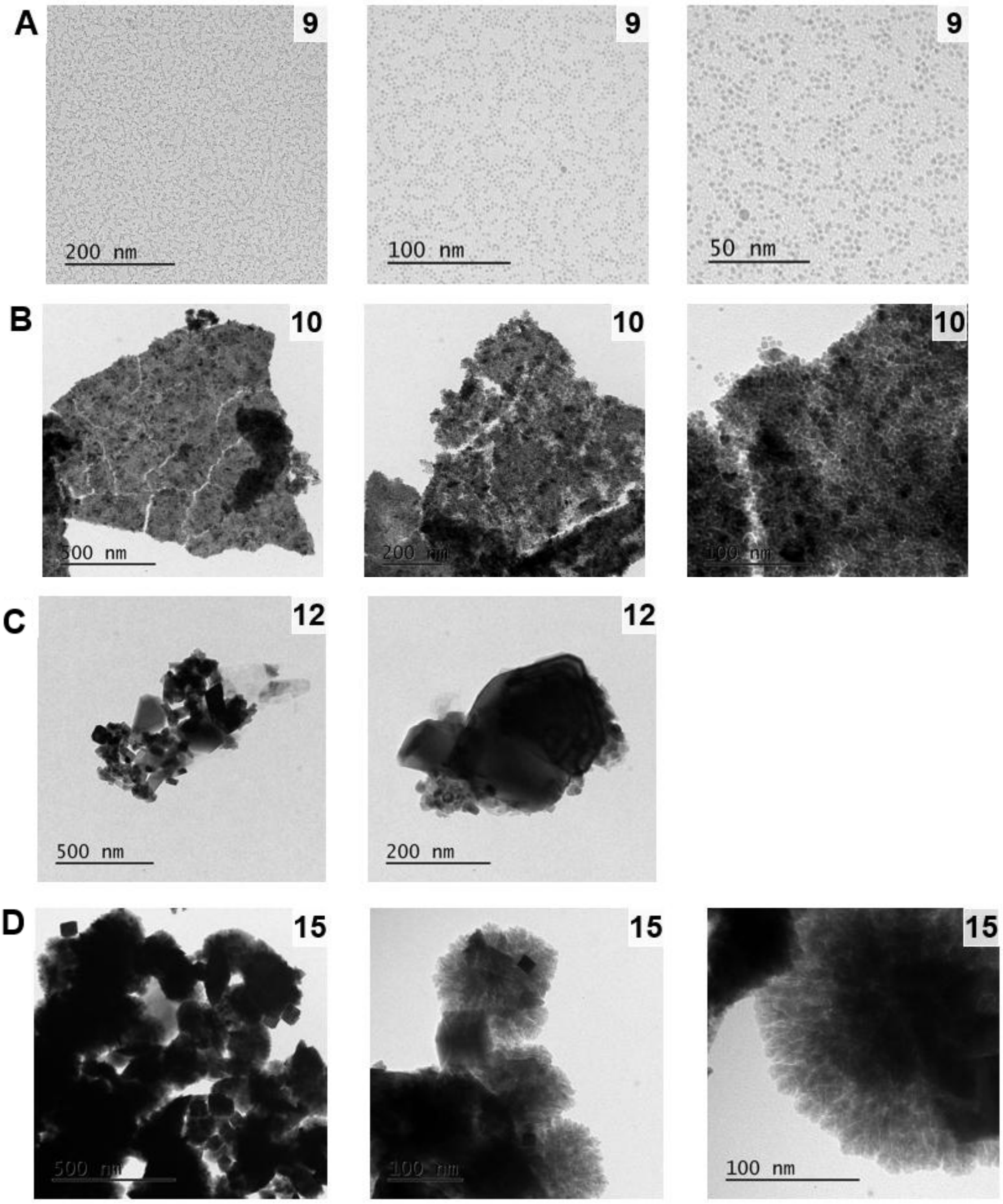

Figure S36. Bright-field TEM images at different magnifications of the NCs obtained when $NiCl_2$ reacts with different ligands in ODE during a 20-minute reaction at 250°C; with ligand 9 in (A), with ligand 10 in (B), with ligand 12 in (C) and with ligand 15 in (D).

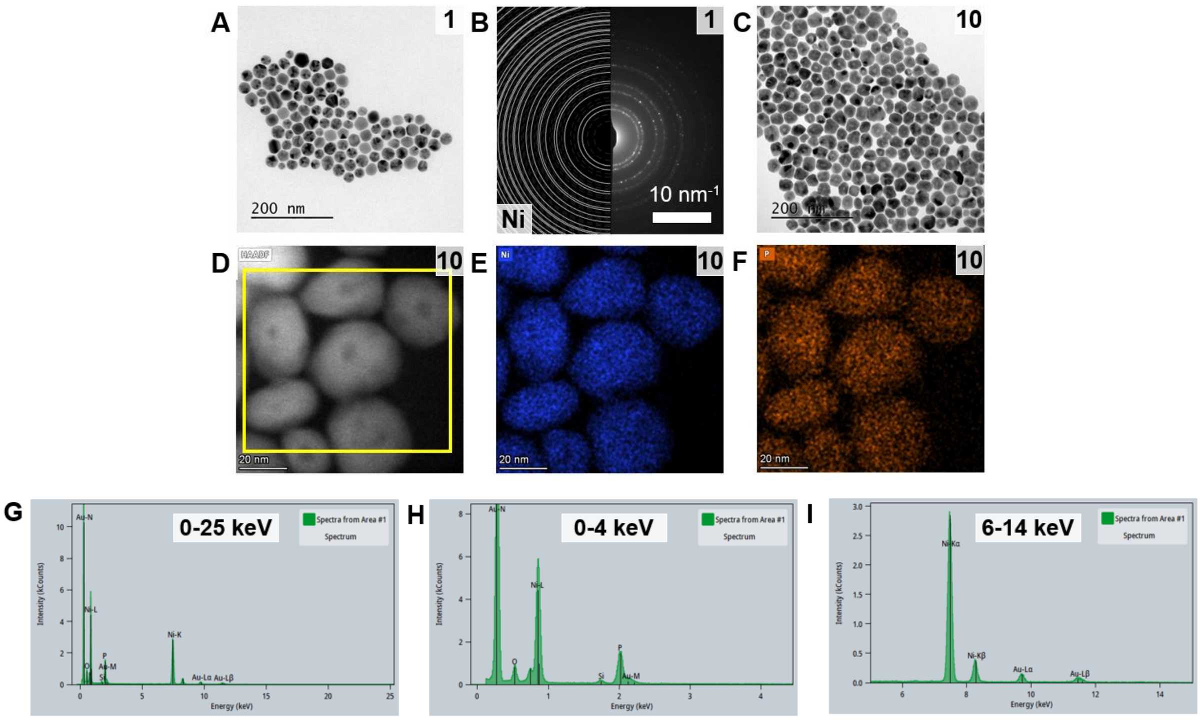

Figure S37. Bright-field TEM image (A) and SAED (B) of the initial Ni NCs synthesized with ligand 1. (C) Bright-field TEM image of the NCs obtained after heating the NCs from (A) with ligand 10 at 250°C for 20 minutes in OLAM. (D) HAADF-STEM image of NCs from (C) with the yellow line delimiting the region of interest for the STEM-EDX spectrum. (E-G) STEM-EDX map of Ni (E) and P (F). (G-I) STEM-EDX spectrum of (D) ranging from 0 to 25 keV (G) with zooms in the 0-4 keV region (H) and in the 6-14 keV region (I). The gold signals come from the grid.

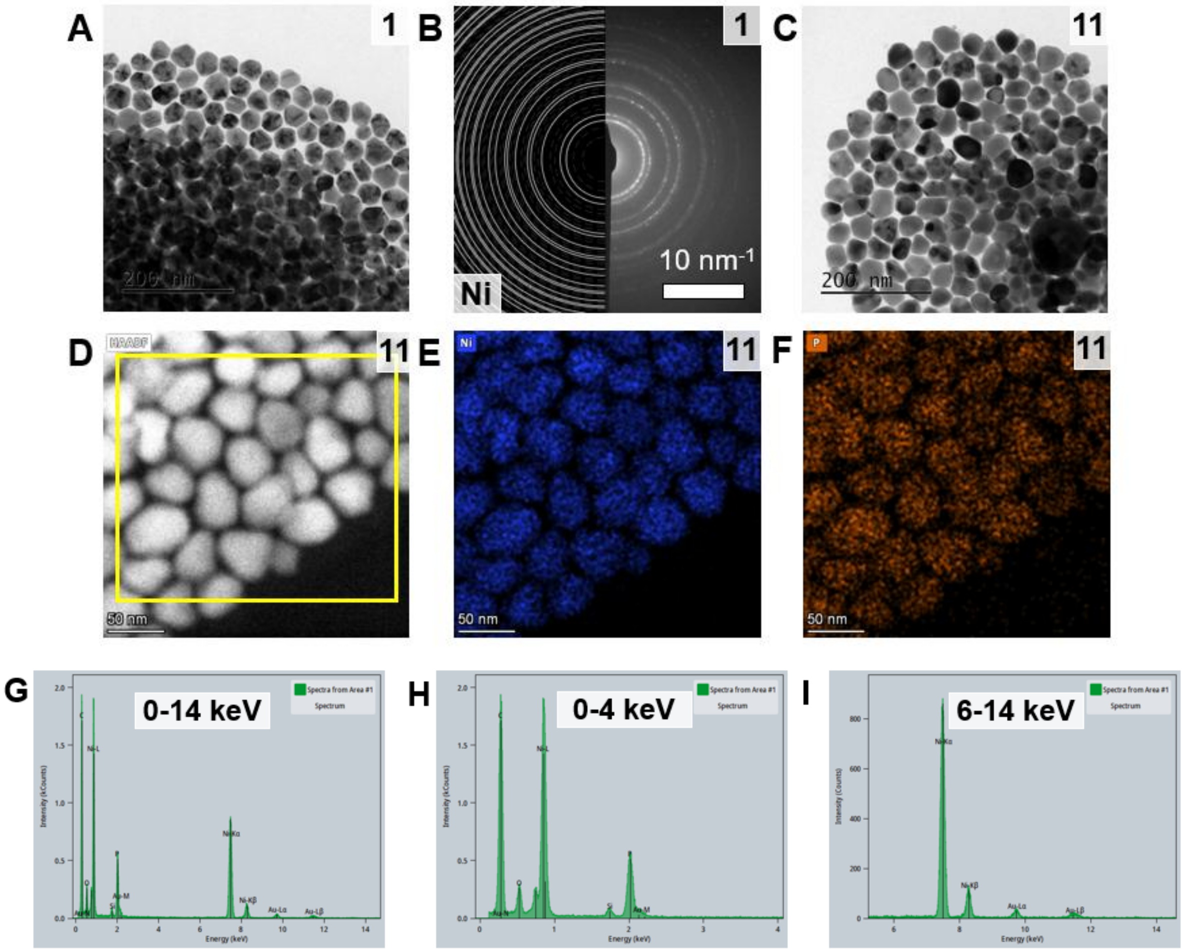

Figure S38. Bright-field TEM image (A) and SAED (B) of the initial Ni NCs synthesized with ligand 1. (C) Bright-field TEM image of the NCs obtained after heating the NCs from (A) with ligand 11 at 250°C for 20 minutes in OLAM. (D) HAADF-STEM image of NCs from (C) with the yellow line delimiting the region of interest for the STEM-EDX spectrum. (E-G) STEM-EDX map of Ni (E) and P (F). (G-I) STEM-EDX spectrum of (D) ranging from 0 to 14 keV (G) with zooms in the 0-4 keV region (H) and in the 6-14 keV region (I). The gold signals come from the grid.

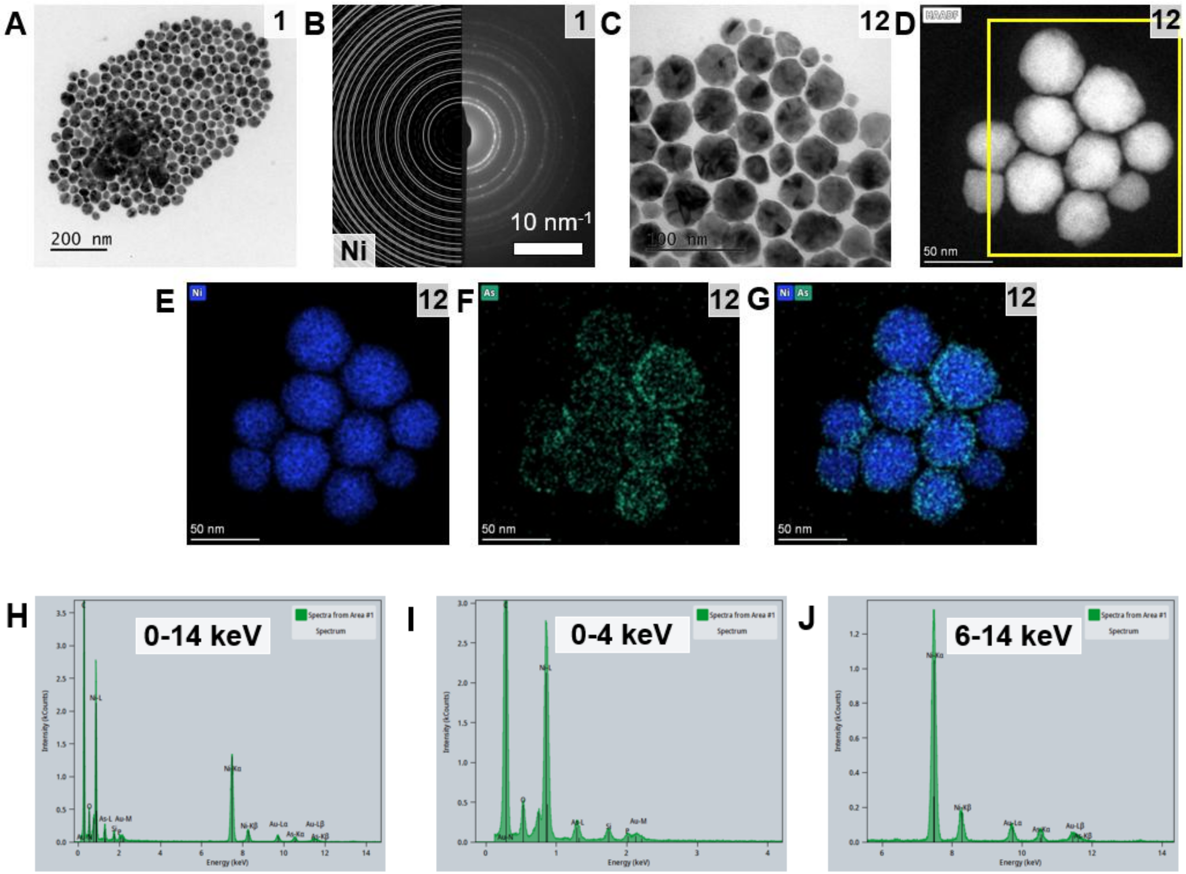

Figure S39. Bright-field TEM image (A) and SAED (B) of the initial Ni NCs synthesized with ligand 1. (C) Bright-field TEM image of the NCs obtained after heating the NCs from (A) with ligand 12 at 250°C for 20 minutes in OLAM. (D) HAADF-STEM image of NCs from (C) with the yellow line delimiting the region of interest for the STEM-EDX spectrum. (E-G) STEM-EDX map of Ni (E), As (F) and both Ni and As (G). (H-J) STEM-EDX spectrum of (D) ranging from 0 to 14 keV (H) with zooms in the 0-4 keV region (I) and in the 6-14 keV region (J). The gold signals come from the grid.

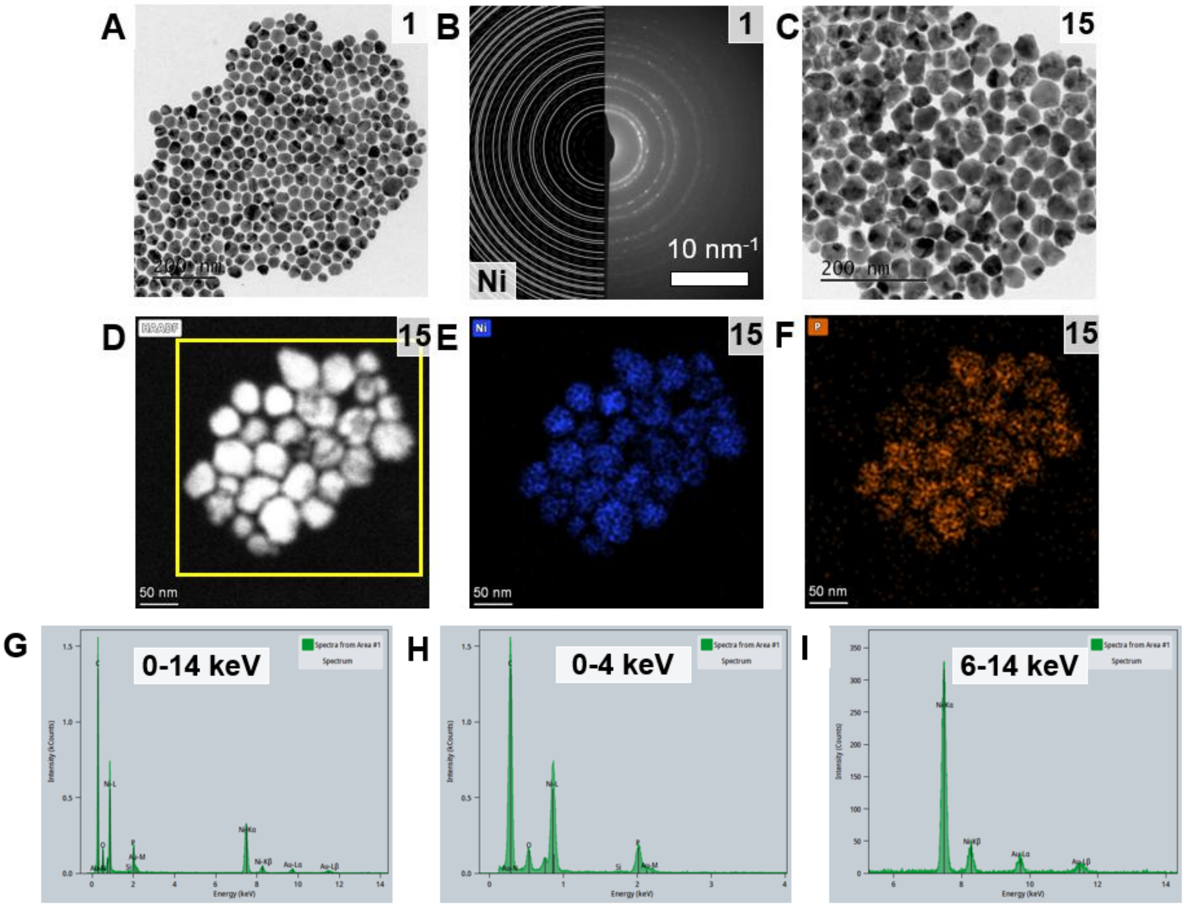

Figure S40. Bright-field TEM image (A) and SAED (B) of the initial Ni NCs synthesized with ligand 1. (C) Bright-field TEM image of the NCs obtained after heating the NCs from (A) with ligand 15 at 250°C for 20 minutes in OLAM. (D) HAADF-STEM image of NCs from (C) with the yellow line delimiting the region of interest for the STEM-EDX spectrum. (E-G) STEM-EDX map of Ni (E) and P (F). (G-I) STEM-EDX spectrum of (D) ranging from 0 to 14 keV (G) with zooms in the 0-4 keV region (H) and in the 6-14 keV region (I). The gold signals come from the grid.

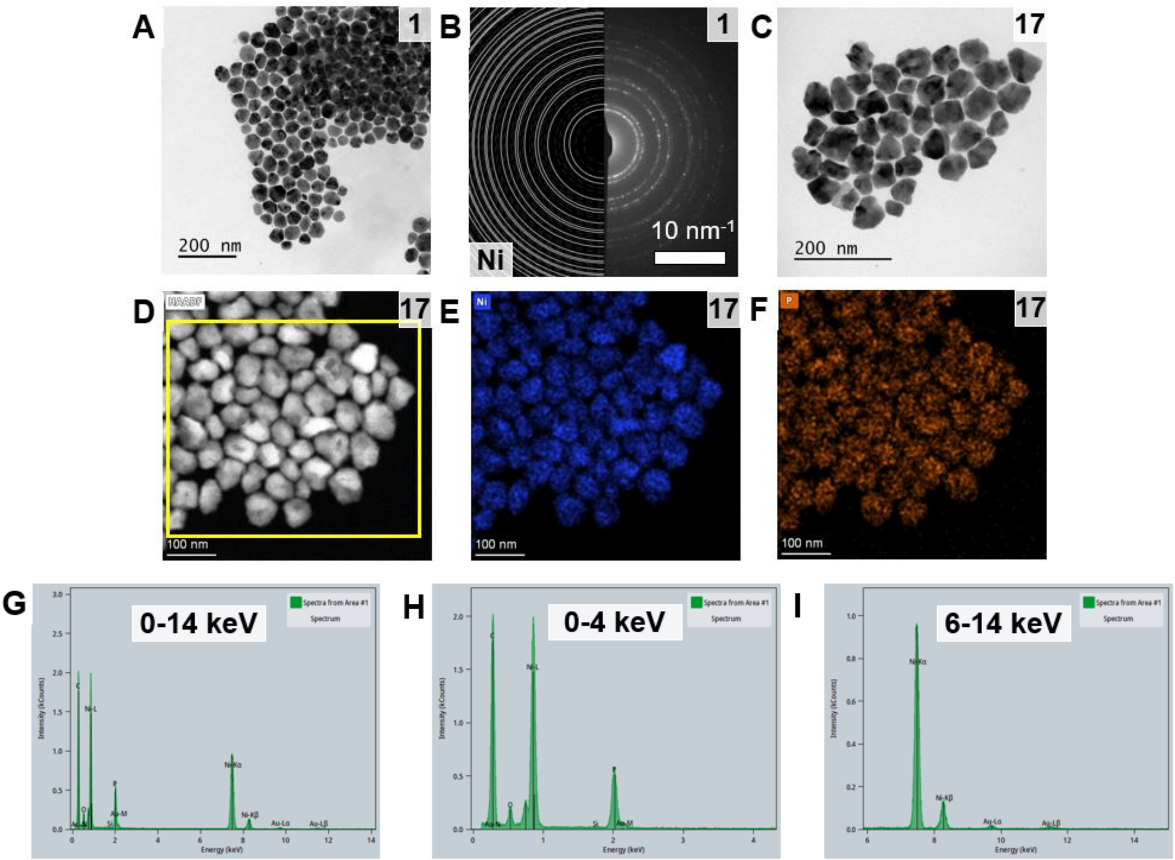

Figure S41. Bright-field TEM image (A) and SAED (B) of the initial Ni NCs synthesized with ligand 1. (C) Bright-field TEM image of the NCs obtained after heating the NCs from (A) with ligand 17 at 250°C for 20 minutes in OLAM. (D) HAADF-STEM image of NCs from (C) with the yellow line delimiting the region of interest for the STEM-EDX spectrum. (E-G) STEM-EDX map of Ni (E) and P (F). (G-I) STEM-EDX spectrum of (D) ranging from 0 to 14 keV (G) with zooms in the 0-4 keV region (H) and in the 6-14 keV region (I). The gold signals come from the grid.

The ligands forming Ni_xE_y NCs also form Ni_xE_y NCs by reacting with pre-synthesized Ni NCs (**Figures S37-S41**).

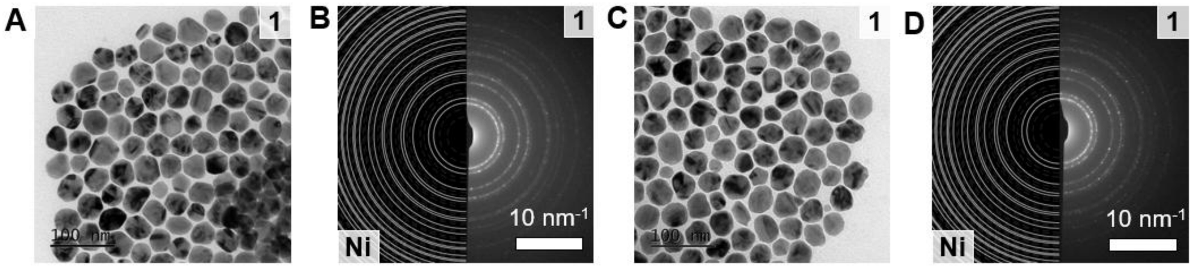

Figure S42. Bright-field TEM image (A) and SAED (B) of the initial Ni NCs synthesized with ligand 1. Bright-field TEM image (C) and SAED (D) of the NCs obtained after heating the NCs from (A) with ligand 1 at 250°C for 20 minutes in OLAM.

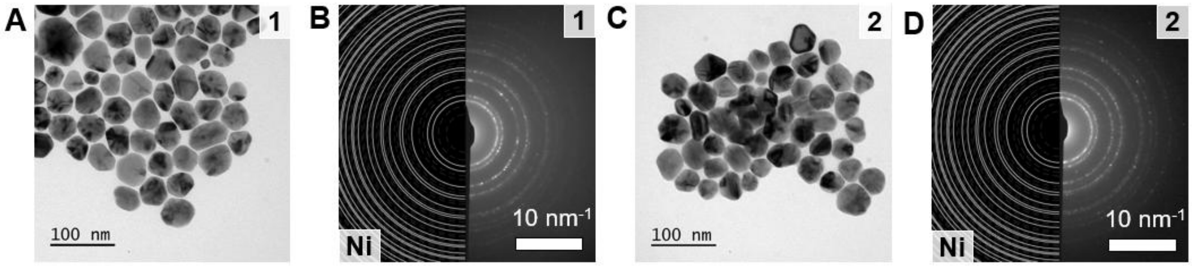

Figure S43. Bright-field TEM image (A) and SAED (B) of the initial Ni NCs synthesized with ligand 1. Bright-field TEM image (C) and SAED (D) of the NCs obtained after heating the NCs from (A) with ligand 2 at 250°C for 20 minutes in OLAM.

In contrast to **Figures S37-S41**, the ligands forming Ni NCs do not react with pre-synthesized Ni NCs and the composition remains Ni(0) (**Figures S42-S43**).

Reference

(1) Cordente, N.; Respaud, M.; Senocq, F.; Casanove, M. J.; Amiens, C.; Chaudret, B. Synthesis and Magnetic Properties of Nickel Nanorods. *Nano Letters* **2001**, *1* (10), 565–568. https://doi.org/10.1021/nl0100522.

(2) Bradley, J. S.; Tesche, B.; Busser, W.; Maase, M.; Reetz, M. T. Surface Spectroscopic Study of the Stabilization Mechanism for Shape- Selectively Synthesized Nanostructured Transition Metal Colloids. *Journal of the American Chemical Society* **2000**, *122* (19), 4631–4636. https://doi.org/10.1021/ja992409y.

(3) Wang, Z.; Chen, Y.; Zeng, D.; Zhang, Q.; Peng, D. L. Solution Synthesis of Triangular and Hexagonal Nickel Nanosheets with the Aid of Tungsten Hexacarbonyl. *CrystEngComm* **2016**, *18* (8), 1295–1301. https://doi.org/10.1039/C5CE02187A.

(4) Leng, Y.; Li, Y.; Li, X.; Takahashi, S. Improved Magnetic Anisotropy of Monodispersed Triangular Nickel Nanoplates. *Journal of Physical Chemistry C* **2007**, *111* (18), 6630–6633. https://doi.org/10.1021/jp0676686.

(5) Carenco, S.; Boissière, C.; Nicole, L.; Sanchez, C.; Le Floch, P.; Mézailles, N. Controlled Design of Size-Tunable Monodisperse Nickel Nanoparticles. *Chemistry of Materials* **2010**, *22* (4), 1340–1349. https://doi.org/10.1021/cm902007g.

(6) Lagrow, A. P.; Ingham, B.; Cheong, S.; Williams, G. V. M.; Dotzler, C.; Toney, M. F.; Jefferson, D. A.; Corbos, E. C.; Bishop, P. T.; Cookson, J.; Tilley, R. D. Synthesis, Alignment, and Magnetic Properties of Monodisperse Nickel Nanocubes. *Journal of the American Chemical Society* **2012**, *134* (2), 855–858. https://doi.org/10.1021/ja210209r.

(7) Stern, L.-A.; Feng, L.; Song, F.; Hu, X. Ni2P as a Janus Catalyst for Water Splitting: The Oxygen Evolution Activity of Ni2P Nanoparticles. *Energy & Environmental Science* **2015**, *8* (8), 2347–2351. https://doi.org/10.1039/C5EE01155H.

(8) Chen, Y.; She, H.; Luo, X.; Yue, G.-H.; Peng, D.-L. Solution-Phase Synthesis of Nickel Phosphide Single-Crystalline Nanowires. *Journal of Crystal Growth* **2009**, *311* (4), 1229–1233. https://doi.org/10.1016/j.jcrysgro.2008.11.094.

(9) Popczun, E. J.; McKone, J. R.; Read, C. G.; Biacchi, A. J.; Wiltrout, A. M.; Lewis, N. S.; Schaak, R. E. Nanostructured Nickel Phosphide as an Electrocatalyst for the Hydrogen Evolution Reaction. *Journal of the American Chemical Society* **2013**, *135* (25), 9267–9270. https://doi.org/10.1021/ja403440e.

(10) Muthuswamy, E.; Savithra, G. H. L.; Brock, S. L. Synthetic Levers Enabling Independent Control of Phase, Size, and Morphology in Nickel Phosphide Nanoparticles. *ACS Nano* **2011**, *5* (3), 2402–2411. https://doi.org/10.1021/nn1033357.

(11) Wang, J.; Johnston-Peck, A. C.; Tracy, J. B. Nickel Phosphide Nanoparticles with Hollow, Solid, and Amorphous Structures. *Chemistry of Materials* **2009**, *21* (19), 4462–4467. https://doi.org/10.1021/cm901073k.

(12) Andaraarachchi, H. P.; Thompson, M. J.; White, M. A.; Fan, H.-J.; Vela, J. Phase-Programmed Nanofabrication: Effect of Organophosphite Precursor Reactivity on the Evolution of Nickel and Nickel Phosphide Nanocrystals. *Chemistry of Materials* **2015**, *27* (23), 8021–8031. https://doi.org/10.1021/acs.chemmater.5b03506.

(13) Li, H.; Lu, S.; Sun, J.; Pei, J.; Liu, D.; Xue, Y.; Mao, J.; Zhu, W.; Zhuang, Z. Phase-Controlled Synthesis of Nickel Phosphide Nanocrystals and Their Electrocatalytic Performance for the Hydrogen Evolution Reaction. *Chemistry – A European Journal* **2018**, *24* (45), 11748–11754. https://doi.org/10.1002/chem.201801964.

(14) Thompson, D.; Hoffman, A. S.; Mansley, Z. R.; York, S.; Wang, F.; Zhu, Y.; Bare, S. R.; Chen, J. Synthesis of Amorphous and Various Phase-Pure Nanoparticles of Nickel

Phosphide with Uniform Sizes via a Trioctylphosphine-Mediated Pathway. *Inorganic Chemistry* **2024**, *63* (40), 18981–18991. https://doi.org/10.1021/acs.inorgchem.4c03334.
(15) Pan, Y.; Liu, Y.; Zhao, J.; Yang, K.; Liang, J.; Liu, D.; Hu, W.; Liu, D.; Liu, Y.; Liu, C. Monodispersed Nickel Phosphide Nanocrystals with Different Phases: Synthesis, Characterization and Electrocatalytic Properties for Hydrogen Evolution. *Journal of Materials Chemistry A* **2015**, *3* (4), 1656–1665. https://doi.org/10.1039/C4TA04867A.
(16) Li, D.; Senevirathne, K.; Aquilina, L.; Brock, S. L. Effect of Synthetic Levers on Nickel Phosphide Nanoparticle Formation: Ni5P4 and NiP2. *Inorganic Chemistry* **2015**, *54* (16), 7968–7975. https://doi.org/10.1021/acs.inorgchem.5b01125.
(17) Bellato, F.; Ferri, M.; Annamalai, A.; Prato, M.; Leoncino, L.; Brescia, R.; De Trizio, L.; Manna, L. Colloidal Synthesis of Nickel Arsenide Nanocrystals for Electrochemical Water Splitting. *ACS Applied Energy Materials* **2023**, *6* (1), 151–159. https://doi.org/10.1021/acsaem.2c02698.
(18) Qian, Y.; Yang, J.; Li, H.; Xing, S.; Yang, Q. Solution-Based Synthesis of NiSb Nanoparticles for Electrochemical Activity in Hydrogen Evolution Reaction. *Chinese Journal of Chemical Physics* **2019**, *32* (3), 373–378. https://doi.org/10.1063/1674-0068/cjcp1805113.
(19) Cai, C.; Lee, H.; Shi, W.; Liu, Y.; Zhang, B.; Sun, L.; Wang, T. Nickel–Antimony Electrocatalyst for Durable Acidic Hydrogen Evolution Reaction in Proton Exchange Membrane Electrolyzers. *ACS Energy Letters* **2025**, 1483–1490. https://doi.org/10.1021/acsenergylett.5c00233.
(20) Tolman, C. A. Phosphorus Ligand Exchange Equilibriums on Zerovalent Nickel. Dominant Role for Steric Effects. *Journal of the American Chemical Society* **1970**, *92* (10), 2956–2965. https://doi.org/10.1021/ja00713a007.
(21) Tolman, C. A. Steric Effects of Phosphorus Ligands in Organometallic Chemistry and Homogeneous Catalysis. *Chemical Reviews* **1977**, *77* (3), 313–348. https://doi.org/10.1021/cr60307a002.
(22) Kühl, O. Phosphorus-31 NMR Spectroscopy: A Concise Introduction for the Synthetic Organic and Organometallic Chemist. *Phosphorus-31 NMR Spectroscopy: A Concise Introduction for the Synthetic Organic and Organometallic Chemist* **2009**, 1–131. https://doi.org/10.1007/978-3-540-79118-8.
(23) Clarke, M. L.; Frew, J. J. R. Ligand Electronic Effects in Homogeneous Catalysis Using Transition Metal Complexes of Phosphine Ligands. In *Organometallic Chemistry*; Royal Society of Chemistry: Cambridge; pp 19–46. https://doi.org/10.1039/b801377m.
(24) Pignolet, L. M. *Homogeneous Catalysis with Metal Phosphine Complexes*; Springer US, 1983. https://doi.org/10.1007/978-1-4613-3623-5.
(25) Montelatici, S.; Van Der Ent, A.; Osborn, J. A.; Wilkinson, G. Further Studies on the Homogeneous Hydrogenation of Olefins by Use of Tris (Tertiary Phosphine)Chlororhodium(I) Complexes. *Journal of the Chemical Society A: Inorganic, Physical, Theoretical* **1968**, No. 0, 1054–1058. https://doi.org/10.1039/J19680001054.
(26) Chen, L.; Ren, P.; Carrow, B. P. Tri(1-Adamantyl)Phosphine: Expanding the Boundary of Electron-Releasing Character Available to Organophosphorus Compounds. *Journal of the American Chemical Society* **2016**, *138* (20), 6392–6395. https://doi.org/10.1021/jacs.6b03215.
(27) Farina, V.; Krishnan, B. Large Rate Accelerations in the Stille Reaction with Tri-2-Furylphosphine and Triphenylarsine as Palladium Ligands: Mechanistic and Synthetic Implications. *Journal of the American Chemical Society* **1991**, *113* (25), 9585–9595. https://doi.org/10.1021/ja00025a025.

(28) McAuliffe, C. A. Phosphorus, Arsenic, Antimony and Bismuth Ligands. In *Comprehensive Coordination Chemistry*; Wilkinson, G., Gillard, R., McCleverty, J. A., Eds.; Pergamon Press, 1987; pp 989–1066.
(29) Levason, W.; Matthews, M. L.; Reid, G.; Webster, M. Synthesis and Properties of New Ditertiary Stibines Based upon o -, m - or p -Xylyl and m - or p -Phenylene Backbones and Their Complexes with Tungsten, Iron and Nickel Carbonyls. *Dalton Transactions* **2004**, *0* (1), 51–58. https://doi.org/10.1039/B312083J.
(30) Okamoto, H. Ni-P (Nickel-Phosphorus). *Journal of Phase Equilibria and Diffusion* **2010**, *31* (2), 200–201. https://doi.org/10.1007/s11669-010-9664-1.
(31) Singleton, M.; Nash, P. The As−Ni (Arsenic-Nickel) System. *Journal of Phase Equilibria* **1987**, *8* (5), 419. https://doi.org/10.1007/BF02893150.
(32) Okamoto, H. Ni-Sb (Nickel-Antimony). *Journal of Phase Equilibria and Diffusion* **2009**, *30* (3), 301–302. https://doi.org/10.1007/s11669-009-9513-2.
(33) Chow, K. K.; Levason, W.; McAuliffe, C. A. Transition Metal Complexes Containing Phosphine Ligands. In *Transition Metal Complexes of Phosphorus, Arsenic and Antimony Ligands*; Macmillan Education UK: London, 1973; pp 33–204. https://doi.org/10.1007/978-1-349-81558-6_2.
(34) Thananatthanachon, T.; Lecklider, M. R. Synthesis of Dichlorophosphinenickel(II) Compounds and Their Catalytic Activity in Suzuki Cross-Coupling Reactions: A Simple Air-Free Experiment for Inorganic Chemistry Laboratory. *Journal of Chemical Education* **2017**, *94* (6), 786–789. https://doi.org/10.1021/acs.jchemed.6b00273.
(35) Leemans, J.; Dümbgen, K. C.; Minjauw, M. M.; Zhao, Q.; Vantomme, A.; Infante, I.; Detavernier, C.; Hens, Z. Acid–Base Mediated Ligand Exchange on Near-Infrared Absorbing, Indium-Based III–V Colloidal Quantum Dots. *Journal of the American Chemical Society* **2021**, *143* (11), 4290–4301. https://doi.org/10.1021/jacs.0c12871.
(36) Parvizian, M.; Duràn Balsa, A.; Pokratath, R.; Kalha, C.; Lee, S.; Van den Eynden, D.; Ibáñez, M.; Regoutz, A.; De Roo, J. The Chemistry of Cu3N and Cu3PdN Nanocrystals. *Angewandte Chemie International Edition* **2022**, *61* (31), e202207013. https://doi.org/10.1002/anie.202207013.
(37) De Roo, J. Chemical Considerations for Colloidal Nanocrystal Synthesis. *Chemistry of Materials* **2022**, *34* (13), 5766–5779. https://doi.org/10.1021/acs.chemmater.2c01058.
(38) Mathiesen, J. K.; Ashberry, H. M.; Pokratath, R.; Gamler, J. T. L.; Wang, B.; Kirsch, A.; Kjær, E. T. S.; Banerjee, S.; Jensen, K. M. Ø.; Skrabalak, S. E. Why Colloidal Syntheses of Bimetallic Nanoparticles Cannot Be Generalized. *ACS Nano* **2024**, *18* (39), 26937–26947. https://doi.org/10.1021/acsnano.4c08835.
(39) Quasdorf, K. W.; Garg, N. K. Bis(Tricyclohexylphosphine)Dichloronickel. In *Encyclopedia of Reagents for Organic Synthesis*; John Wiley & Sons, Ltd: Chichester, UK, 2010. https://doi.org/10.1002/047084289X.rn01201.
(40) Luh, T.-Y.; Yuan, T.-M.; Kobayashi, Y. Dichlorobis(Triphenylphosphine)Nickel(II). In *Encyclopedia of Reagents for Organic Synthesis*; John Wiley & Sons, Ltd: Chichester, UK, 2009. https://doi.org/10.1002/047084289X.rd100.pub2.
(41) Leineweber, A.; Jacobs, H. Preparation and Crystal Structures of Ni(NH3)2Cl2 and of Two Modifications of Ni(NH3)2Br2 and Ni(NH3)2I2. *Journal of Solid State Chemistry* **2000**, *152* (2), 381–387. https://doi.org/10.1006/jssc.2000.8666.
(42) Marcoux, D.; Charette, A. B. Palladium-Catalyzed Synthesis of Functionalized Tetraarylphosphonium Salts. *The Journal of Organic Chemistry* **2008**, *73* (2), 590–593. https://doi.org/10.1021/jo702355c.
(43) Till, M.; Streitferdt, V.; Scott, D. J.; Mende, M.; Gschwind, R. M.; Wolf, R. Photochemical Transformation of Chlorobenzenes and White Phosphorus into

Arylphosphines and Phosphonium Salts. *Chemical Communications* **2022**, *58* (8), 1100–1103. https://doi.org/10.1039/D1CC05691C.
(44) Marcoux, D.; Charette, A. B. Nickel-Catalyzed Synthesis of Phosphonium Salts from Aryl Halides and Triphenylphosphine. *Advanced Synthesis & Catalysis* **2008**, *350* (18), 2967–2974. https://doi.org/10.1002/adsc.200800542.
